# Deep phenotyping of the lipidomic response in COVID‐19 and non‐COVID‐19 sepsis

**DOI:** 10.1002/ctm2.1440

**Published:** 2023-11-10

**Authors:** Hu Meng, Arjun Sengupta, Emanuela Ricciotti, Antonijo Mrčela, Divij Mathew, Liudmila L. Mazaleuskaya, Soumita Ghosh, Thomas G. Brooks, Alexandra P. Turner, Alessa Soares Schanoski, Nicholas F. Lahens, Ai Wen Tan, Ashley Woolfork, Greg Grant, Katalin Susztak, Andrew G. Letizia, Stuart C. Sealfon, E. John Wherry, Krzysztof Laudanski, Aalim M. Weljie, Nuala J. Meyer, Garret A. FitzGerald

**Affiliations:** ^1^ Institute for Translational Medicine and Therapeutics Perelman School of Medicine University of Pennsylvania Philadelphia Pennsylvania USA; ^2^ Department of Systems Pharmacology and Translational Therapeutics Perelman School of Medicine University of Pennsylvania Philadelphia Pennsylvania USA; ^3^ Institute for Immunology and Immune Health Perelman School of Medicine University of Pennsylvania Philadelphia Pennsylvania USA; ^4^ Department of Medicine Perelman School of Medicine University of Pennsylvania Philadelphia Pennsylvania USA; ^5^ Department of Neurology Icahn School of Medicine at Mount Sinai New York New York USA; ^6^ Department of Genetics Perelman School of Medicine University of Pennsylvania Philadelphia Pennsylvania USA; ^7^ Naval Medical Research Center Silver Spring Maryland USA; ^8^ Naval Medical Research Unit TWO Singapore Singapore; ^9^ Department of Anesthesiology and Critical Care Perelman School of Medicine University of Pennsylvania Philadelphia Pennsylvania USA

## Abstract

**Background:**

Lipids may influence cellular penetrance by viral pathogens and the immune response that they evoke. We deeply phenotyped the lipidomic response to SARs‐CoV‐2 and compared that with infection with other pathogens in patients admitted with acute respiratory distress syndrome to an intensive care unit (ICU).

**Methods:**

Mass spectrometry was used to characterise lipids and relate them to proteins, peripheral cell immunotypes and disease severity.

**Results:**

Circulating phospholipases (sPLA2, cPLA2 (PLA2G4A) and PLA2G2D) were elevated on admission in all ICU groups. Cyclooxygenase, lipoxygenase and epoxygenase products of arachidonic acid (AA) were elevated in all ICU groups compared with controls. sPLA2 predicted severity in COVID‐19 and correlated with TxA2, LTE4 and the isoprostane, iPF2α‐III, while PLA2G2D correlated with LTE4. The elevation in PGD2, like PGI2 and 12‐HETE, exhibited relative specificity for COVID‐19 and correlated with sPLA2 and the interleukin‐13 receptor to drive lymphopenia, a marker of disease severity. Pro‐inflammatory eicosanoids remained correlated with severity in COVID‐19 28 days after admission. Amongst non‐COVID ICU patients, elevations in 5‐ and 15‐HETE and 9‐ and 13‐HODE reflected viral rather than bacterial disease. Linoleic acid (LA) binds directly to SARS‐CoV‐2 and both LA and its di‐HOME products reflected disease severity in COVID‐19. In healthy marines, these lipids rose with seroconversion. Eicosanoids linked variably to the peripheral cellular immune response. PGE2, TxA2 and LTE4 correlated with T cell activation, as did PGD2 with non‐B non‐T cell activation. In COVID‐19, LPS stimulated peripheral blood mononuclear cell PGF2α correlated with memory T cells, dendritic and NK cells while LA and DiHOMEs correlated with exhausted T cells. Three high abundance lipids – ChoE 18:3, LPC‐O‐16:0 and PC‐O‐30:0 – were altered specifically in COVID. LPC‐O‐16:0 was strongly correlated with T helper follicular cell activation and all three negatively correlated with multi‐omic inflammatory pathways and disease severity.

**Conclusions:**

A broad based lipidomic storm is a predictor of poor prognosis in ARDS. Alterations in sPLA2, PGD2 and 12‐HETE and the high abundance lipids, ChoE 18:3, LPC‐O‐16:0 and PC‐O‐30:0 exhibit relative specificity for COVID‐19 amongst such patients and correlate with the inflammatory response to link to disease severity.

## Graphical abstract

Sepsis is accompanied with eicosanoid storm. Secretory phospholipase A2 (sPLA2) serves as an immunolipidomic hub regulating COVID‐19 induced sepsis. sPLA2‐driven selective PGD2 elevation was observed in COVID‐19 subjects. sPLA2‐driven depletion of ether lipids may function as a restraint on the inflammatory response.

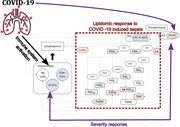

## 1 INTRODUCTION

The biological importance of membrane lipids has long been recognised, both as barriers to infection and, once cleaved, as circulating or locally acting modulators of the immune response to pathogens.^1^, ^2^, ^3^ While perturbation of the serum lipidome by infections is well documented,^4^, ^5^, ^6^, ^7^, ^8^ attention has focused on high abundance lipids such as phospholipids, ceramides and sphingomyelins. This has been true also in COVID‐19 where the high abundance lipidome has been implicated in prognosis,^9^ pathogenesis^10^ and disease severity.^11^, ^12^, ^13^, ^14^, ^15^, ^16^, ^17^, ^18^, ^19^, ^20^

Relatively few reports have focused on eicosanoids, less abundant oxylipins that act locally, but are nonetheless powerful modulators of the immune response.^4^ Elevated levels of eicosanoids have been reported in plasma,^21^ tracheal aspirate,^22^ urine^23^ and in stimulated peripheral blood monocytes^24^ of patients with severe COVID‐19. Snider et al.^25^ proposed the eicosanoid generating enzyme, group IIA secretory (s) phospholipase (PL)A_2_, as a marker of disease severity in COVID‐19. However, many published studies, including this last, compare the lipid response in patients with that in healthy volunteers, so it is difficult to know the specificity of the response for infection with SARS‐CoV‐2 compared with other infections.

Both statins^26^, ^27^ and drugs modulating eicosanoid formation or action may be of benefit in preventing or treating COVID‐19.^28^, ^29^, ^30^ However, none, to date, including aspirin, has been validated by randomised control trials.^31^ Despite early concerns, treatment with other non‐steroidal anti‐inflammatory drugs does not appear to affect adversely the clinical course of COVID‐19.^32^

Here, we compare the impact of severe COVID‐19 on lipids in plasma, urine and peripheral blood mononuclear cells (PBMCs) with that in both healthy volunteers and patients with sepsis caused by other viruses or bacteria at unprecedented scale. We integrate this response with clinical parameters, the plasma proteome and peripheral immune cells to reveal immunoregulatory hubs driven by elements of the lipidome that predict disease severity and patient prognosis. This unique comprehensive integrative approach defines the relative specificity of these responses. We also addressed the hypothesis that a milder lipidomic signal of inflammation might accompany symptomatic seroconversion in healthy individuals infected with SARS‐CoV‐2. Finally, we provide a network analysis tool to generate novel mechanistic and therapeutic hypotheses.

## 2 METHODS

### 2.1 Human populations and clinical data preparation

Patients were recruited into two research programs at the University of Pennsylvania. All protocols were approved by the Institutional Review Board and were in accordance with the Declaration of Helsinki. Clinical data on the enrolled participants were collected from the electronic medical records (EMRs). Sample sizes were based on availability of biological samples rather than a prespecified effect size.

The first, the COVID‐19 Immune Prediction for Lung Recovery study (PICOBS; #IRB843311), was part of the COVID‐19 BioBank Collection (IRB#813913) and recruited prospectively from patients admitted to the Penn Presbyterian Medical Center between April 2020 through September 2020. All patients were hospitalised and had a positive SARS‐CoV‐2 test by reverse transcription polymerase chain reaction (RT‐PCR). Vulnerable populations, such as children or pregnant women were excluded. After securing consent the patient's blood and urine was drawn at enrolment and then 2, 7 and 90 days thereafter, according to the patient's availability in either in‐ or outpatients’ settings.

The larger research program involved patients recruited to one of three prospectively enrolling cohort studies. Hospitalised inpatients admitted either to the Hospital of the University of Pennsylvania or the Penn Presbyterian Medical Center with acute illness due to SARS‐CoV‐2 were eligible for the Molecular Epidemiology of SepsiS in the intensive care (MESSI‐COVID, IRB# 808542) study if they were confirmed to have a positive SARS‐CoV‐2 by RT‐PCR and the reason for hospitalisation was adjudicated by physician investigators as related to the infection.^33^, ^34^, ^35^, ^36^ Prior to March 2020, subjects were eligible for the same cohort (MESSI) if they were admitted to the medical intensive care unit (ICU) with sepsis and acute organ failure.^35^, ^37^, ^38^ Most MESSI subjects had confirmed bacterial infection.^38^ Subjects were excluded if they were admitted to the hospital from a long‐term acute care hospital signifying chronic critical illness; if they desired exclusively palliative measures on admission; or if the subject or their proxies were unwilling or unable to provide informed consent prior to discharge or within 3 days of admission. Study personnel screened all hospitalised patients with positive SARS‐CoV‐2 tests daily, or prior to 2020, screened all patients admitted to the ICU for sepsis and performed informed consent discussions by phone or in person with the subject if he or she were capable of informed consent discussions or with the patient's proxy by phone if the subject was incapacitated due to illness.

Subjects who provided informed consent had blood drawn at enrolment (time 0) which was within 72 h of admission and repeated on days 2 and 7. Urine was also collected at these timepoints. Clinical data were abstracted from the EMR into standardised case report forms which included demographic detail, comorbidities and medications. Clinical laboratory data were obtained from the EMR for the date closest to the time of blood draw. COVID‐19 severity was categorised using a scale adapted for clinical trials from the World Health Organization ordinal scale as described.^37^ Severity of illness was assessed using the Acute Physiology and Chronic Health Evaluation III (APACHE III)^39^ score based on physiologic data at the time of enrolment. Thus, the distinction between moderate and severe cases was based on an ordinal scale (1, not hospitalised, capable of resuming normal activities; 2, not hospitalised but unable to resume normal activities; 3, hospitalised, not requiring O_2_ supplementation; 4, hospitalised and requiring O_2_ therapy; 5, hospitalised and requiring high flow nasal O_2_ therapy, non‐invasive mechanical ventilation (MV), or both; 6, ICU hospitalisation, requiring invasive MV or extracorporeal membrane oxygenation, or both; 7, death), with scores 1−4 designating mild or moderate cases and scores 5−7 severe cases. Acute kidney injury was defined according to the Kidney Disease Improving Global Outcomes creatinine criteria^40^ and defining baseline creatinine as the average of outpatient or hospital discharge creatinine values from days 365 days before to 7 days before hospital admission.^41^, ^42^ If prior creatinine values were missing, baseline creatinine was defined as the lowest value prior to enrolment.^42^ Survival status was assessed at 30 and 90 days.

Plasma and serum were collected after spinning the tube at 1000×*g*, 10 min, at 4°C. Aliquot plasma and serum samples were stored at −80°C. Urine was collected in the morning and aliquots were stored at −80°C.

Plasma, PBMC and urine samples were also collected from the healthy volunteers (IRB#826459).

Serum samples were also obtained from healthy US Marines who participated in a study conducted by the Icahn School of Medicine at Mount Sinai and the Naval Medical Research Center under a protocol approved by the institutional IRBs.^43^ The study protocol was approved by the Naval Medical Research Center Institutional Review board (protocol number NMRC.2020.0006) in compliance with all applicable Federal regulations governing the protection of human subjects. The volunteers underwent a 2‐week quarantine at home followed by a second supervised 2‐week quarantine at a closed college campus that involved mask wearing, social distancing and daily temperature and symptom monitoring. Study volunteers were tested for SARS‐CoV‐2 by RT‐PCR assay of nares swab specimens obtained between the time of arrival and the second day of supervised quarantine and on days 7 and 14. Serum specimens were obtained at enrolment, the day when participants converted positive by RT‐PCR during infection and post infections. Aliquot serum samples were stored at −80°C. A subset of these serum samples was transferred to the University of Pennsylvania for quantitative measurement of eicosanoids.

Demographic data on the clinical studies are provided in Table 1.

**TABLE 1:** Demographics of samples used in different assays presented in this study.

				Age			BMI			
Study	Assay	Subject	*N*	Q1	Med	Q3	Q1	Med	Q3	%Male
MESSI	Flow cytometry	COVID	127	48	60	68.5	25.44	28.65	35.64	51.18
		Non‐COVID	2	63	63	63	29.27	32.97	36.66	0
	Mass cytometry	COVID	12	48.25	58.5	71.25	24.51	28.8	34.38	41.67
	Plasma Eicosanoids	COVID	164	48.75	60	69	25.46	28.9	37.15	54.27
		Control	16	47.25	56.5	61.25	23.7	26.5	28.03	50
		Non‐COVID	251	52	61.5	71.18	22.79	26.03	30.52	57.37
	Plasma ECs	COVID	14	55.5	59.5	69	22.01	27.28	39.42	42.86
		Control	16	47.25	56.5	61.25	23.7	26.5	28.03	50
		Non‐COVID	48	52	60.1	71.25	23.43	25.48	29.95	56.25
	Plasma proteins	COVID	171	48.5	60	69	25.43	28.85	36.8	54.39
		Control	16	47.25	56.5	61.25	23.7	26.5	28.03	50
		Non‐COVID	148	53.53	61.68	70.53	23.46	26.19	30.63	56.08
	Plasma lipids	COVID	75	49.5	60	69.5	26.49	30.34	40.22	56
		Control	16	47.25	56.5	61.25	23.7	26.5	28.03	50
		Non‐COVID	66	52	60.36	67.19	22.81	26.19	29.55	68.18
	Urine eicosanoids	COVID	176	42	57	70	25.67	30.71	38.08	53.15
		Control	26	54.25	61	69	24.75	27.11	27.88	50
		Non‐COVID	11	54.75	71	75	23.9	25.37	33	50
PICOBS	PBMC eicosanoids	COVID	10	52.75	59.5	62.5	22.95	29.39	34.65	40
		Control	13	45	55	61	23.96	26.59	28.97	38.46
	PBMC flow cytometry	COVID	11	54.5	60	62	23.32	28.22	34.64	45.45
		Control	16	47.25	56.5	61.25	23.7	26.5	28.03	50

### 2.2 Eicosanoids

#### 2.2.1 Chemicals and reagents

The following standard compounds and their deuterated analogues were purchased from Cayman Chemicals: Creatinine, d3‐Creatinine, PGEM (11α‐hydroxy‐9,15‐dioxo‐2,3,4,5‐tetranor‐prostane‐1,20‐dioic acid), PGDM (11,15‐dioxo‐9‐hydroxy‐2,3,4,5‐tetranorprostane‐1,20‐dioic acid), PGIM (2,3‐dinor‐6‐keto‐PGF_1α_), TxM (11deHydro‐TxB_2_), 8,12‐*iso*‐iPF_2α_‐VI, iPF_2α_‐III, d6‐PGEM, d6‐PGDM, d3‐PGIM, d4‐TxM, d11‐8,12‐*iso*‐iPF_2α_‐VI, d4‐iPF_2α_‐III, 14(15)‐dihydroxy epoxyeicosatrienoic acid (DHET), 11(12)‐DHET, 8(9)‐DHET, 5(6)‐DHET, 14(15)‐EET, 11(12)‐EET, 8(9)‐EET, 15‐hydroxy eicosatetraenoic acid (HETE), 12‐HETE, 5‐HETE, LTB_4_, LTE_4_, 20‐OH‐LTB_4_, 13‐hydroxy‐octadecenoate (HODE), 9‐HODE, 12(13)‐dihydroxyoctadecenoic acid (DiHOME), 9(10)‐DiHOME, d11‐14(15)‐DHET, d11‐11(12)‐DHET, d11‐8(9)‐DHET, d11‐14(15)‐EET, d11‐11(12)‐EET, d11‐8(9)‐EET, d4‐LTB_4_, d5‐LTE_4_, d8‐5‐HETE, d8‐12‐HETE, d8‐15‐HETE, d4‐12(13)‐DiHOME, d4‐13‐HODE, d4‐12(13)‐EpOME, PGE_2_, PGD_2_, PGF_2α_, 6‐keto‐PGF_1α_, TxB_2_, linoleic acid (LA), arachidonic acid (AA), eicosapentaenoic acid (EPA), docosahexanoic acid (DHA), lipoxin (Lx)A4, LxB4, resolvin (Rv)D1, RvD2, RvD3, RvE1, RvD5, 18‐hydroxyeicosapentaenoic acid (HEPE), d4‐PGE_2_, d4‐PGD_2_, d4‐PGF_2α_, d4‐6‐keto‐PGF_1α_, TxB_2_.

#### 2.2.2 Sample collection

Plasma for eicosanoid analysis was separated from whole blood samples by centrifugation at 4°C, snap‐frozen and stored at −80°C. Spot urine samples were also collected within 48 h of admission and stored at −80°C.

The COVID‐19 group included 153 plasma and 176 urine samples drawn from the programs above. The healthy volunteer group included 16 plasma and 26 urine samples, and the non‐COVID‐19 ICU patient group included 249 plasma and 11 urine samples.

Aliquots of plasma and urine samples were thawed at room temperature (RT) just before sample preparation for liquid chromatography‐mass spectrometry (LC–MS) analysis of eicosanoids.

### 2.3 LC–MS analysis of eicosanoid metabolites in urine

Two aliquots of 500 μL urine were processed separately. The first aliquot (A) was for the analysis of PGEM, PGDM, PGIM, TxM, 8,12‐*iso*‐iPF_2α_‐VI and iPF_2α_‐III. Stable isotope‐labelled internal standards were added to 0.5 mL of urine. The internal standards used were d6‐PGEM (25 ng), d6‐PGDM (25 ng), d3‐PGIM (5 ng), d4‐TxM (5 ng), d11‐8,12‐*iso*‐iPF_2α_‐VI (5 ng) and d4‐iPF_2α_‐III (5 ng) in 50 μL of acetonitrile. Methoxyamine (MO) HCl solution (250 μL of 100 g MO HCl solid in 100 mL water) was added, and the sample was allowed to equilibrate for 30 min. The sample solution was brought to a total volume of 1 mL by adding 200 μL of water. The second aliquot (B) was for the measurement of 14(15)‐DHET, 11(12)‐DHET, 8(9)‐DHET, 5(6)‐DHET, 14(15)‐EET, 11(12)‐EET, 8(9)‐EET, 15‐HETE, 12‐HETE, 5‐HETE, LTB_4_, LTE_4_, 20‐OH‐LTB_4_, 13‐HODE, 9‐HODE, 12(13)‐DiHOME and 9(10)‐DiHOME, an internal standard solution containing 5 ng of deuterated standards of d11‐14(15)‐DHET, d11‐11(12)‐DHET, d11‐8(9)‐DHET, d11‐14(15)‐EET, d11‐11(12)‐EET, d11‐8(9)‐EET, d4‐LTB_4_, d5‐LTE_4_, d8‐5‐HETE, d8‐12‐HETE, d8‐15‐HETE, d4‐12(13)‐DiHOME, d4‐13‐HODE and d4‐12(13)‐EpOME in 50 μL acetonitrile was added to 0.5 mL of each urine sample. Two aliquots of urine were purified by solid phase extraction (SPE) using Strata‐X 33 μm polymeric reversed phase cartridges (Phenomenex, 8B‐S100‐TAK). The eluate was collected and dried using an Eppendorf vacufuge, and the residue was reconstituted in 100 μL of 50% methanol in water before injection into the HPLC–MS/MS system. For Aliquot A, separation was carried out using a Waters ACQUITY UPLC system with a ultra‐performance liquid chromatography (UPLC) column, 2.1 × 150 mm with 1.7 μm particles (Waters ACQUITY UPLC CSH C18) and a mobile phase consisting of water with 0.5% ammonium acetate at pH 5.7 (mobile phase A) and acetonitrile–methanol mixture at a ratio of 95:5 (mobile phase B). The flow rate was 350 μL/min, and a linear solvent gradient from 5 to 45% mobile phase B over 30 min was used for separation. Aliquot B will be analysed by the same HPLC conditions as plasma and serum samples. Quantification was performed using a single point calibration with a standard mix. Our multiple reaction monitoring (MRM) LC–MS method has a linear range of 4 magnitudes, and the one‐point calibration we utilised was chosen and all analyte's concentration levels in our samples were below the concentration where the calibration curve starts to bend. We also reported values below the lowest level of a typical calibration curve, but above the limit of detection (LOD).

To normalise the urinary metabolite concentrations, urinary creatinine was quantified by LC–MS. A stable isotope‐labelled internal standard (10 μg/mL d3‐creatinine in 3% H₂O/acetonitrile) was added to 10 μL of each urine sample, and the mixture was diluted with 200 μL acetonitrile. Separation was carried out with a UPLC column, 2.1 × 50 mm with 2.5 μm particles (Waters XBridge BEH HILIC) and a mobile phase consisting of 100% acetonitrile (mobile phase A) and 5 mM ammonium formate water solution (pH = 3.98) (mobile phase B) with a flow rate of 350 μL/min.

### 2.4 LC–MS analysis of eicosanoid metabolites in plasma and serum

For the analysis of eicosanoid metabolites in plasma (including PGE_2_, PGD_2_, PGF_2α_, 6‐keto‐PGF_1α_, TxB_2_, 8,12‐*iso*‐iPF_2α_‐VI, iPF_2α_‐III, 14(15)‐DHET, 11(12)‐DHET, 8(9)‐DHET, 5(6)‐DHET, 14(15)‐EET, 11(12)‐EET, 8(9)‐EET, 15‐HETE, 12‐HETE, 5‐HETE, LTB_4_, LTE_4_, 20‐OH‐LTB_4_, 13‐HODE, 9‐HODE, 12(13)‐DiHOME, 9(10)‐DiHOME, LA, AA), a 50 μL sample was mixed with 300 μL of acetonitrile internal standard solution containing 5 ng of deuterated standards for each metabolite (except 1000 ng for d8‐AA). Proteins in the plasma or serum sample were then precipitated out with the organic solvent and the sample was centrifuged at 21,000×*g* for 2 min. The supernatant was transferred to a Phree Phospholipid Removal cartridge (Phenomenex; 8B‐S133‐TAK) and the eluate was collected and dried in an Eppendorf vacufuge. The resulting residue was reconstituted in 100 μL of 50% methanol in water, transferred to an autosampler vial, and 30 μL of the sample was injected into the HPLC–MS/MS system. The chromatography was carried out using a Waters ACQUITY UPLC system with a UPLC column, 2.1 × 150 mm with 1.7 μm particles (Waters ACQUITY UPLC BEH C18) with a mobile phase A prepared from water and mobile phase B prepared from 95:5 (v/v) acetonitrile:methanol, both containing 0.1% formic acid. The flow rate was 350 μL/min, and separations were carried out with various linear solvent gradients. Quantitation was done by a single point calibration with a standard mix and processed with each batch of samples.

### 2.5 LC–MS analysis of specialised pro‐resolving mediators

We adopted LC–MS methods^44^ using MRM to analyse specialised pro‐resolving mediators (SPMs) and two related fatty acids. To create a standard solution for single point calibration, we prepared a solution of 100 ng/mL of EPA, DHA, LxA_4_, LxB_4_, RvD1, RvD2, RvD3, RvE1, RvD5 and 18‐HEPE. We followed the same procedures used for eicosanoid measurement in plasma, serum, urine, PBMC cell culture media and endobronchial washings to process the standard solution. Subsets of patient groups were tested for detection of SPMs. Assays in plasma and serum were performed in five healthy subjects; urine samples were tested in five healthy volunteers and five COVID‐19 patients with severe disease; endobronchial washing samples from 10 patients with severe COVID‐19 and 10 samples from patients with non‐COVID‐19 sepsis; and all PBMC cell culture media samples were tested. We used the same internal standards as in the corresponding eicosanoid measurements, with the deuterated lipid compound closest to each SPM peak serving as the internal standard for that compound. The LOD was determined using a signal‐to‐noise (S/N) ratio approach, calculated as three times the S/N ratio. In the case of EPA and DHA, the LODs were established at 0.3 ng/mL for urine assays and 3 ng/mL for other types of samples. For SPMs, the LODs were determined to be 3 × 10^−4^ ng/mL for urine samples and 3 × 10^−3^ ng/mL for other sample types. In no case did we find evidence for SPM formation within these limits, so we did not proceed to further analyses in the larger clinical cohorts.

### 2.6 Endocannabinoid analysis: anandamide and 2‐arachidonoylglycerol in plasma

Endocannabinoids (ECs) were extracted from 50 μL plasma, based on a previously described protocol.^45^ Briefly, plasma was added to 500 μL of chilled methanol/Tris buffer [50 mmol/L, pH8] containing internal standards. To this mixture was added 500 μL of Tris buffer and 1.5 mL of (2:1) methanol–chloroform. This mixture was centrifuged 500×*g* for 2 min, and the chloroform phase was removed to a glass tube. This extraction was repeated twice, and the combined mixture was dried. The dried residue was reconstituted in chloroform. Further, 2 mL of acetone was added to it and the solution was centrifuged at 16,000×*g* for 5 min. The clear supernatant was collected, dried and reconstituted in 100 μL methanol. ECs were analysed by LC‐MS/MS. MS/MS analysis was performed using Waters Xevo TQ‐S instrument equipped with electrospray ionisation. Mobile phase A consisted of water/B (95/5) with 0.1% formic acid and mobile phase B consisted of (95/5) acetonitrile and methanol with 0.1% formic acid. A gradient used in the run is as follows: 0 min 30% B; 5 min 50% B; 15 min 100% B; 17 min 100% B; 17.5 min 30% B; 20.5 min 30% B at a flow rate of 0.25 mL/min with a total run time of 20.5 min. Positive electrospray ionisation data were acquired using MRM. The mass transitions, collision energy, cone used were: anandamide (AEA) (348.2/287.3, 14, 30), d4 AEA (352.1/287.3, 14, 30), 2‐arachidonoylglycerol (2‐AG): (379.4/287.2, 14, 30); d5 2‐AG: (384.4/287.4, 14, 30).

### 2.7 DESI‐MS analysis of high abundance lipids in blood plasma

Metabolites were extracted from blood plasma samples (50 μL) using a modified Bligh‐dyer biphasic extraction protocol described elsewhere.^46^ The nonpolar fraction of the extract was used for DESI‐MS based lipidomic analysis. 1 μL samples were spotted onto a PTFE‐coated slide and analysed using high‐throughput screening DESI‐MS. Slides that were not immediately analysed were stored at −80°C and warmed up to RT for analysis.

DESI‐MS experiments were completed using a XevoG2‐XS QToF mass spectrometer (Waters) with a two‐dimensional DESI stage source (Prosolia) with optimised parameters. A red sharpie and black Staedtler marker were used to assess calibration. The solvent system consisted of 98% UPLC–MS grade methanol (Fisher Scientific) and 2% MilliQ water (Millipore Sigma) with 50 pg/μL leucine‐enkephalin (used as a lock spray reference; Waters) for negative mode. The same composition was used for positive mode with the addition of 0.1% formic acid (Fisher Scientific) to help with ionisation efficiency. Experiments were completed using a flow rate of 2 μL/min with a Harvard syringe pump.

Raw data were exported and analysed using RStudio (version 4.12 Posit; PBC) and the MSI.EAGLE package developed in house. This package provides a graphical user interface via R Shiny and uses the Cardinal package (version 2.10) for mass spectral analysis.^47^, ^49^ Data were processed using 10% of randomly sampled pixels for peak picking using median absolute deviation method. Minimum peak frequency was 0.01. For peak binning, 15 ppm tolerance was allowed. Segmentation UMAP analyses^48^ were used to isolate each sample spot. Targeted data analysis was performed by mapping the picked peaks on a recently identified set of serum lipids.^49^

### 2.8 Analysis of Peripheral Blood Mononuclear Cells (PBMCs)

PBMCs were collected from 11 PICOBS COVID‐19 patients (six of them were admitted to the ICU) from the PICOBS cohort and 16 healthy controls (Table 1).

Frozen PBMCs were thawed and resuspended in RPMI 1640 media supplemented with 10% FBS. After centrifugation, the cell pellet was resuspended in RPMI 1640 media supplemented with 2% FBS and split in two aliquots. One aliquot of PBMC was used for flow‐cytometry analysis and another aliquot was added in one well of a U‐bottom 12‐well non‐treated plate (Thermo Fisher) and incubated at 37°C and 5% CO_2_ for 18 h. After incubation, the 12‐well plate was centrifuged. The cell culture medium was collected for the lipidomic analysis.

*Flow‐cytometry analysis of PBMCs*. High‐dimensional flow cytometry analysis was performed after thawing frozen PBMCs from COVID‐19 patients and healthy controls. The flow‐cytometry panel allowed for simultaneous detection of the T‐lymphocytes (naïve, cytotoxic, central memory, effector memory and EMRA phenotypes of CD8+ T‐cells and CD4+ T cells, exhausted like CD8+ T‐cells; CD4+ follicular helper T cells); B‐lymphocytes; monocytes (classical and non‐classical); natural killer cells and dendritic cells populations as previously described.^35^ Cells were stained for viability exclusion using Live/Dead Ghost Dye in PBS for 10 min with Fc Block at RT. Cells were then washed and stained for 30 min at RT with the surface antibody master mix containing antibodies (Table S1) in fluorescence‐activated cell sorting buffer (PBS containing 2% foetal bovine serum) and Brilliant stain buffer (BD Biosciences). After RT incubation, cells were washed and fixed with 1% PFA prior to data acquisition on a BD LSR cytometer II (BD Biosciences). Data were analysed using FlowJo software (version 10.6.2; Tree Star).

*Data and materials availability*: All compensated flow cytometry files are publicly available at https://hpap.pmacs.upenn.edu/explore/download?otherdonor

*Lipidomic analysis of PBMC cell culture samples*. Oxylipins were measured by LC–MS as described above. Briefly, 800 uL of the PBMCs medium was used for SPE. A cocktail of stable isotope‐labelled internal standards was added to each sample in 50 μL of acetonitrile prior to SPE. SPE was conducted using Strata X, 30 mg, 1 mL tubes (Phenomenex). The cartridge was conditioned with 1 mL acetonitrile followed by 0.25 mL water. Samples were loaded to the cartridge followed by washing with 1 mL solvent constituting 5% (methanol/water). The cartridges were dried by vacuum for 15 mins and samples were eluted with 0.1% formic acid in 1 mL methanol. The extracts were dried under a stream of nitrogen and reconstituted in 120 μL solvent constituting 50:50 (v/v) methanol and water. The samples were transferred to the autosampler tube, 30 μL of samples were injected to UPLC–MS/MS. A Waters Acquity UPLC BEH column (2.1 × 150 mm) was used for the separation of the compounds. Mobile phase A consisted of H_2_O/B (95/5) + 0.1% formic acid. Mobile phase B consisted of acetonitrile/methanol (95/5) + 0.1% formic acid. Following gradient was run: 0 min 20% B; 5 min 2% B; 6 min 10% B; 12 min 60% B; 15 min 2% B; at a flow rate of 0.35 mL min^−1^. A Waters Xevo TQ‐S instrument was run in negative ionisation mode for detection of the compounds.

### 2.9 Measurement of phospholipases in plasma

Plasma Group 11A secretory phospholipaseA_2_ (sPLA_2_‐IIA) concentrations were determined by ELISA (Cayman Chemical). Plasma samples were diluted (1:40−1:2560) and were subjected to the instructions provided by the manufacturer. Concentrations of sPLA_2_‐IIA in plasma were calculated using standard curves.

Plasma Group IID secretory phospholipase A_2_ (PLA_2_G2D) was also measured in plasma by ELISA (My Biosource). Plasma samples were diluted fourfold using PBS (pH = 7.0) and assayed as per manufacturer's instruction. Concentrations were calculated using standard curves.

Plasma cytosolic (c)PLA_2_ (PLA_2_G4A) was measured as part of the O‐link proteomic analysis, a relative quantification from Ct values.

### 2.10 Proteomic analysis in plasma

Proteomic analysis was performed using the O‐link platform, as previously described.^35^, ^50^ Briefly, whole blood was spun within 2 h of blood collection (3000 rpm, 15 min), and plasma was collected, aliquoted and frozen at −80°C until assay. Plasma was not immunodepleted. We used the O‐link Proximity Extension Assay to measure 713 unique proteins. In this assay, oligonucleotide‐labelled monoclonal or polyclonal antibodies are used to bind each protein target in a pairwise manner upon which the paired oligonucleotides hybridise. The unique hybridisation product is amplified by PCR, and multiplex detection occurs in a high throughput fluidic chip system. On each plate, a common interplate control of pooled 'healthy' plasma acquired and processed at O‐link facilities was used for normalisation resulting in a semiquantitative measurement for each protein on log2‐transformed scale referred to as the normalised protein expression.

### 2.11 Mass cytometry analysis

Patients admitted to the Hospital of the University of Pennsylvania with a positive SARS‐CoV‐2 PCR test were screened and approached for informed consent within 3 days of hospitalisation. Peripheral blood was collected into sodium heparin tubes (BD; catalog no. 367874) and 270 μL of blood was transferred to MDIPA tube which contained a lyophilised panel of 30 markers (StandardBio; catalog no. 201334). Cells were stained for 20 min at RT in the dark followed by red blood lysis. Cells were washed and resuspended in Maxpar Fix and Perm buffer containing 125 nM of Cell‐ID Intercalator‐Ir and kept overnight at 2−4C until sample acquisition. All mass cytometry data were collected on a CyTOF Helios (Standard Biotool).

### 2.12 Statistical analysis

High abundance lipid data were analysed in Rstudio running on R 4.1.2. Non‐parametric statistical analyses (Kruskal–Wallis and Mann–Whitney tests) were performed using base R stats package and in‐house scripts. Statistical significance was reported after correction for multiple testing correction using the Benjamini Hochberg method. All plots were generated using ggplot2 (v3.4.1), pheatmap (v1.0.12) packages and in‐house scripts. High abundance lipid data was analysed in Rstudio running on R 4.1.2. Non‐parametric statistical analysis (Kruskal–Wallis and Mann–Whitney tests) was performed using base R stats package and in house scripts. All plots were generated using ggplot2, pheatmap packages and in house scripts. Multivariate analysis was performed using Simca‐P 17 (Startorius Stedim).

To evaluate the relationship between eicosanoid concentrations and both the disease severity score and the frequency of immune cell subtypes, we computed the Spearman correlation coefficient based on the rank of these variations. We used a *t*‐distribution with *n−*2 degrees of freedom, where *n* is the sample size, to calculate the associated *p* value for the Spearman correlation coefficient. To assess differences in eicosanoid concentrations between different patient groups and the healthy control group, we used the Kruskal–Wallis test, calculated using Prism 9 software.

To assess differences in serum LA metabolite concentrations at different time points (before infection, during infection and after infection) in the US Marines, we used a one‐way analysis of variance (ANOVA) with subsequent pairwise comparisons as appropriate, all calculated using Prism 9 software.

### 2.13 Integrative correlation analysis

The data used in the integration analysis were obtained from non‐COVID ICU patients with sepsis and COVID‐19 patients as described above. Non‐COVID patients were more critically ill than COVID‐19 patients, as reflected by their APACHE scores.

The data comprise measurements of clinical features, proteins, metabolites and cell populations as described above. Table 2 summarises the numbers of the various features used in the integrative analysis. Not all measurements were available across all samples. The sample sizes for several cohorts of interest are shown in Table S2.

**TABLE 2:** Sizes of feature sets used in integrative analysis. Plasma protein features include sPLA_2_ and PLA_2_G2D. Some proteins were measured in multiple Olink panels, and such proteins are represented by two or more, usually highly correlated, features.

	Number of features used in the integrative analysis
Plasma proteins	730
Plasma eicosanoids	18
Urine eicosanoids	18
Positive mode plasma lipids	393
Negative mode plasma lipids	205
Flow cytometry data	216
Mass cytometry data	71
Endocannabinoids	2
Clinical and demographic data	100

Four correlation matrices were obtained by estimating Spearman's correlation coefficients using available measurements from all four cohorts of interest: COVID‐19, non‐COVID ICU, moderate COVID‐19 and severe COVID‐19.

The correlation matrix for the COVID‐19 cohort was transformed into a distance matrix by substituting each correlation coefficient estimate *ρ* with 1−*ρ*.^2^ This distance matrix was provided as an input to UMAP to visualise the correlation network of measured features. Certain pairs of features had insufficient sample size to calculate an estimate of their correlation. The distance between such feature pairs was set to 1 (maximum), and consequently, they were not included as edges of the target simplicial set, making UMAP rely on other feature pairs, for which correlation estimates were feasible, when positioning features in the plane.

To visualise and explore the differences between the correlation network of features in COVID‐19 and non‐COVID ICU cohorts, we calculated the differential correlation matrix using the formula ½ (*ρ*_COVID‐19_ − *ρ*_non‐COVID_) and provided the corresponding distance matrix to UMAP. We also constructed an undirected and unweighted differential correlation graph in which nodes correspond to measured features and the existence of an edge between a pair of features indicates significant (*p* < .05) change between correlation coefficients *ρ*_non‐COVID_ and *ρ*_COVID‐19_ for the pair of features. The *p* values for differential correlation were based on statistics (*Z*_non‐COVID_ − *Z*_COVID‐19_)/((*N*_non‐COVID_ – 3)^−1^ – (*N*_COVID‐19_ – 3)^−1^)^1/2^, where *Z*_non‐COVID_ and *Z*_COVID‐19_ denote the Fisher's *Z*‐transformation of coefficients *ρ*_non‐COVID_ and *ρ*_COVID‐19_, respectively. Features with highest degree centrality and betweenness centrality exhibit the largest differences in how they correlate with other features in COVID‐19 versus non‐COVID‐19 cohorts and these were singled out. A similar analysis was performed to compare moderate COVID‐19 and severe COVID‐19 correlation structures, but it did not produce as many significant results, most likely due to reduced sample sizes (Table S2). Differences were apparent in individual data sets (e.g., urinary eicosanoids) where sample sizes were greater.

The data are presented as an interactive feature browser for further hypothesis generation at sepsis‐multiomics.itmat.org

The analysis was performed using Python 3.10.9 with additional packages SciPy (version 1.10.0), UMAP‐learn (version 0.5.3) and NetworkX (version 2.8.8). STRING^51^ version 11.5 was used for enrichment analyses.

## 3 RESULTS

### 3.1 Phospholipases

sPLA2‐IIA concentrations were measured in plasma from 181 subjects. Figure S1A shows the distribution of sPLA_2_‐IIA in the healthy volunteer, ICU non‐COVID‐19 and ICU COVID‐19 groups. The median (interquartile range) concentration of sPLA_2_‐IIA is elevated significantly in COVID‐19 patients with median values of 53.9 ng/mL (23.7 ng/mL, 173.5 ng/mL; *p* < .0001) and also in non‐COVID patients with sepsis with median values of 123.3 ng/mL (36.06 ng/mL, 356.47 ng/mL; *p* < .0001) with respect to control subjects 9.8 ng/mL (7.4 ng/mL, 22.2 ng/mL).

sPLA_2_‐IIA levels reflected disease severity in COVID‐19 patients and was positively correlated with the associated ordinal score 7 days after admission (Spearman's *ρ* = 0.31, *p* = .01) while also correlating with other indices of disease severity, such as platelet to lymphocyte ratio (PLR), neutrophil to lymphocyte ratio (NLR) and C‐reactive protein (CRP), all of which differed significantly between patients with severe and moderate COVID‐19 (Spearman's *ρ* = 0.38, 0.38, 0.67, *p* < .01). These results are consistent with the suggestion that sPLA_2_ reflects severity in COVID‐19.^25^

sPLA_2_ was positively correlated with diverse biochemical measures which reflected and/or predicted disease severity (Table S3). These included urinary TxM, LPC‐O‐16:0 (see below) and the proteins such as the interleukin 13 receptor subunit alpha 1, disulfide isomerase (P4HB) and E3 ubiquitin protein ligase, indices of lymphocyte and monocyte chemotaxis, such as MCP‐1, MCP‐3, CXCL10, IL‐6 and S100A12 and immune markers of CD4+ and CD8+ T cell activation like HLADR+ and CD38+ in the central memory CD4+ T cells. These observations implicate sPLA_2_ as the likely source of the eicosanoid storm. Positioning of sPLA_2_ within the inflammatory connectome is discussed further below.

In our cohort, plasma concentrations of cPLA_2_ did not discriminate between the COVID‐19 versus non‐COVID‐19 sepsis patients, while PLA_2_G2D was lower in the COVID‐19 patients than in both the non‐COVID sepsis group and the healthy controls (Figures S1B and C). Plasma concentrations of PGE_2_ and 5(6)‐DHET were correlated with cPLA_2_, while plasma 12‐HETE was correlated with PLA_2_G2D.

Interestingly, PLA_2_G2D (negatively) and sPLA_2_ (positively) correlated with blood glucose—the latter relationship as previously described.^52^ PLA_2_G2D was also positively correlated with expression of proteins relevant to lipid metabolism (Perilipin), cellular adhesion (myocillin) and inflammation (C‐type lectin domain family 7 member A : CLEC7A) as previously characterised using the O‐link platform in a subset of the MESSI cohort.^35^

In summary, sPLA_2_ relates to disease severity in COVID‐19 but is also elevated in patients with other forms of sepsis in the ICU. It serves as a source of eicosanoid generation in patients with sepsis and these downstream products themselves reflect and/or predict the course of the disease and correlate with distinct immune features and proteins as described below. This is consistent with their importance in shaping the immune response and clinical outcome in COVID‐19.

### 3.2 Eicosanoids

#### 3.2.1 Urine and plasma eicosanoids in ICU patients with sepsis compared with healthy controls

**FIGURE 1:**
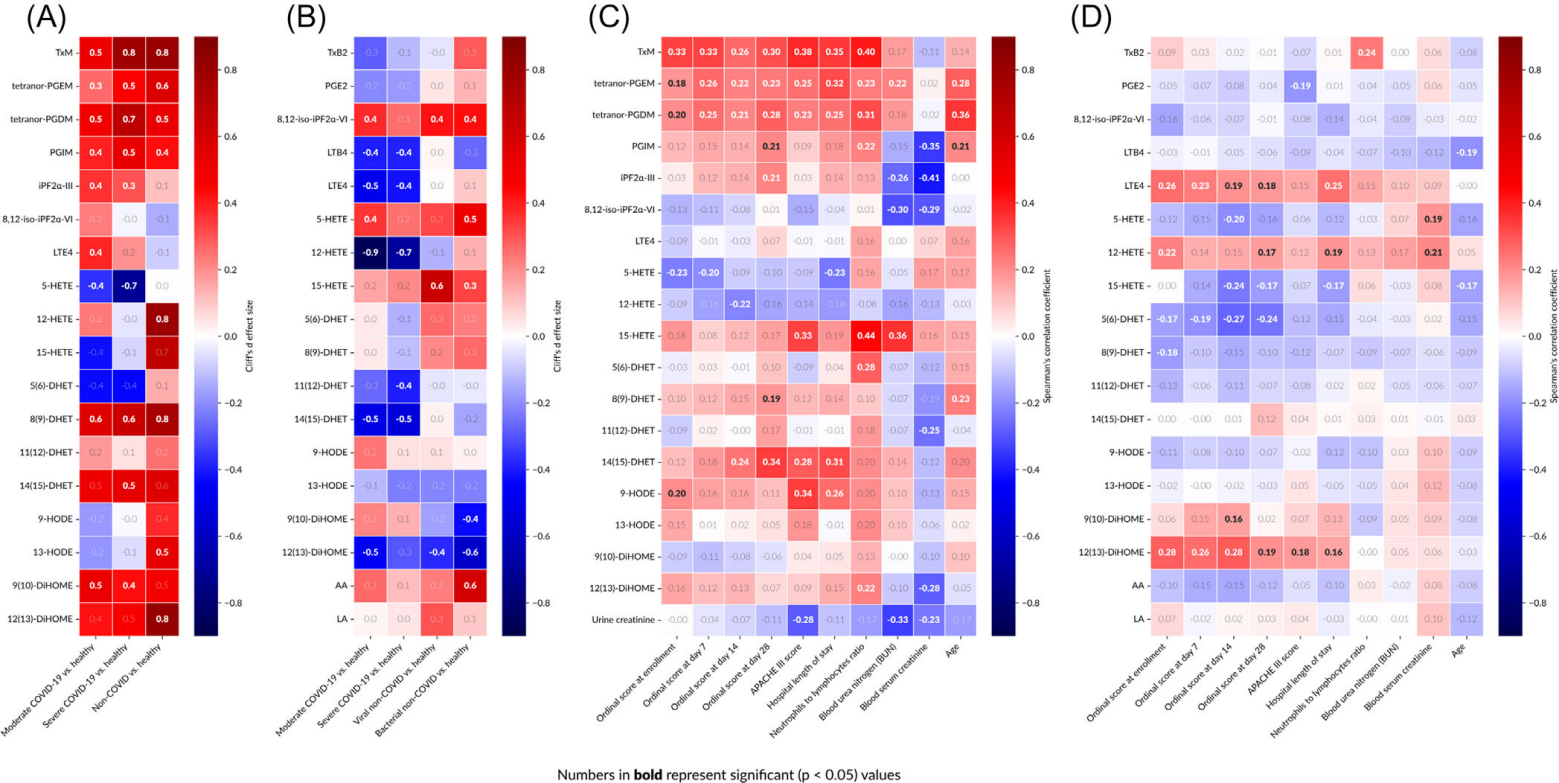
Eicosanoids and disease severity. Comparison of eicosanoid profiles and Spearman correlation with severity scores and organ damage indicators between COVID‐19 patients, non‐COVID patients and healthy controls. (A) Heat maps illustrate the effect size of mean differences of eicosanoids and linoleic acid metabolites in urine, and (B) plasma, when comparing each patient group with healthy controls. The values within each box represent the Cliff's delta, indicating the magnitude of the differences. The significance of these differences was determined using non‐parametric Mann–Whitney tests and significant (*p* < .05) differences are indicated in bold. Additionally, the Spearman correlation coefficient (*ρ*) between eicosanoids and different severity scores and organ damage indicators in (C) urine and (D) plasma are presented. The colour scale represents the strength and direction of the correlation. Warmer colours indicate positive correlations and cooler colours indicate negative correlations. The *p* values were calculated and significant (*p* < .05) correlations are indicated in bold.

The effect size of mean differences of measured eicosanoids and their metabolites between patient groups and healthy controls are shown in Figures 1A and B. The values within each box represent the Cliff's delta, indicating the magnitude of the differences. Cliff's delta is a measure of how often the values in one distribution are larger than the values in a second distribution. Urinary PGEM, PGDM, PGIM and TxM were all significantly elevated in moderate and severe COVID‐19 patients, as well as in non‐COVID ICU patients, when compared with controls (Figure 1A). These patterns were also exhibited by the urinary 8, (9)‐DHET, the isoprostane iPF_2α_‐III and the LA metabolite 9,10‐Di‐HOME (Figure 1A). While the relative elevations of these compounds usually reflected the more severe disease evident in non‐COVID‐19 versus COVID‐19 patients (e.g., PGEM, 5‐ and 15‐HETE, DHETs and DiHOMEs), the elevation in urinary PGDM was more pronounced in COVID patients, despite them having less severe disease.

In plasma, we also observed increased eicosanoid generation in COVID‐19 and non‐COVID ICU patients, including 8,12‐iso‐iPF_2α_, 5‐HETE and 15‐HETE. While the corresponding elevation in AA was evident, this was most pronounced in patients with non‐viral sepsis. In plasma, LTB_4_, LTE_4_, 12‐HETE and 14(15)‐DHET were depressed in both moderate and severe COVID‐19 patients compared with non‐COVID patients with viral and non‐viral infections.

We tested a subset of urine, plasma, serum samples and endobronchial washings but did not observe peaks for LxA4, LxB4, RvD1, RvD2, RvD3, RvE1 or RvD5.

In conclusion, our findings indicate the presence of an eicosanoid storm in patients admitted to the ICU with sepsis caused by COVID‐19 or other pathogens. Notably, urinary PGDM appears to be disproportionately elevated in COVID‐19 patients compared with other sepsis patients, despite their greater disease severity.

#### 3.2.2 Eicosanoids as predictors of disease severity

**FIGURE 2:**
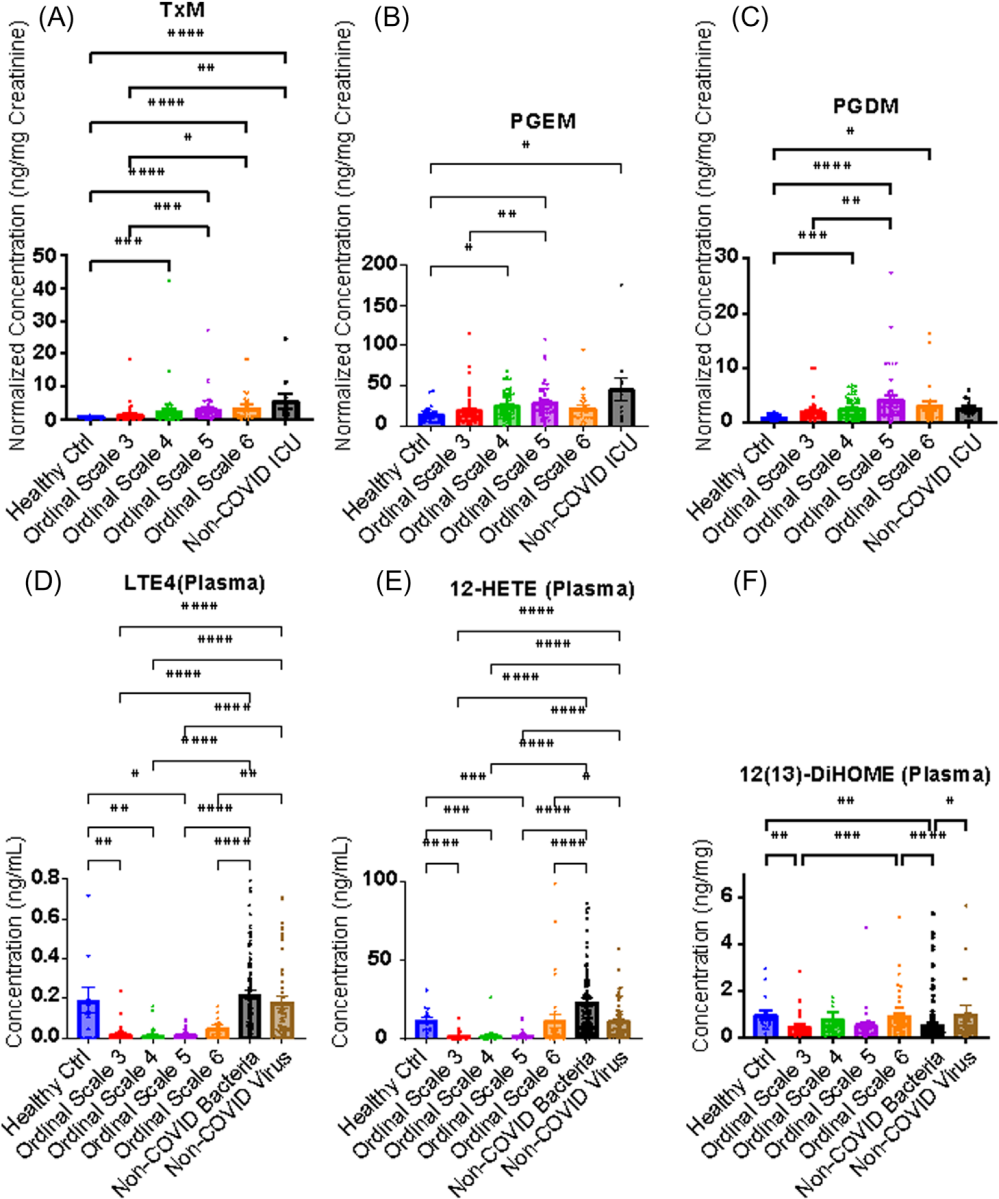
Selected eicosanoids in each severity group. The figure shows the normalised concentrations of urinary TxM (A), PGEM (B), PGDM (C) and concentrations of plasma LTE_4_ (D), 12‐HETE (E), 12^13^‐DiHOME (F) in each severity group, with error bars indicating standard error of the mean. The statistical significance was determined using Kruskal–Wallis test and marked as follows: *p < .05, **p < .01, ***p < .001, ****p < .0001.

Next, we calculated the Spearman correlation between the eicosanoids and different clinical severity scores and indices of organ damage. As expected, metabolites of major pro‐inflammatory eicosanoids, TxM, PGEM and PGDM in urine reflected disease severity correlating with the COVID‐19 ordinal scale described above (Figure 1C). Less pronounced relationships were evident for 14^15^‐DHET, 9‐ and 13‐HODEs, the isoprostanes and 12(13)‐DiHOME. Interestingly, while plasma LTE_4_, 12‐HETE and DiHOMEs showed lower levels in COVID patients compared with healthy controls, within the COVID patient group, they demonstrated significant correlations with the NIH ordinal scale (Figure 1D). Further analysis (Figure 2) showed that levels of urinary TxM, PGEM and PGDM and plasma LTE_4_, 12‐HETE and DiHOMEs increased with disease severity. Thus, more severely ill patients had a more intense inflammatory response and higher levels of metabolites from the COX pathway in urine. Although the levels of LTE_4_, 12‐HETE and DiHOMEs in plasma were not significantly higher than those in healthy controls, these metabolites also increased with disease severity in patients with COVID‐19. These lipids were also correlated with other indicators of disease severity, such as the APACHE III scores, and time in hospital (Figure 1C). A well‐accepted index of COVID‐19 severity, the NLR, correlated positively with urinary TxM, PGEM and PGDM and with LTE_4_ in plasma. Urinary PGEM and PGDM were also positively correlated with age, another marker of disease severity in COVID‐19.

Pro‐inflammatory eicosanoids – reflected by TxM, PGEM, PGDM, PGIM, the isoprostane, iPF_2α_‐III, and 14(15)‐DHET in urine and LTE_4_, 12‐HETE, 12(13)‐DiHOME in plasma – remained correlated with the ordinal scale of disease severity 28 days after admission (Figures 1C and D). Thus, elevation of these pro‐inflammatory lipids at admission is associated with persistent pneumonia severity at day 28.

The ECs‐AEA and 2‐AG ‐ were elevated in COVID‐19 (Figures S1D and E). 2‐AG was significantly correlated with a history of pulmonary disease and disease severity in sepsis patients. It was also correlated with other plasma lipids linked to disease severity (TxM, PGE_2_, LTB_4_ and LPC‐O‐16:0). Similarly, AEA correlated with hospital mortality and 12(13)‐DiHOME.

Bacterial infection triggered a broader eicosanoid response than observed in viral sepsis (Figure S2). In both viral sepsis cohorts, lipid species belonging to the same pathway tended to correlate positively amongst themselves and showed no significant associations with those from different pathways (Figure S2A). In contrast, bacterial sepsis resulted in widespread positive cross correlations amongst products of different eicosanoid pathways (Figure S2B). Here also, eicosanoids strongly correlated with disease severity. Thus, 15‐HETE, 11(12)‐DHET and 14(15)‐DHET positively correlated with the APACHE III disease severity score, while PGE_2_, Tx and 12‐HETE correlated with the NLR and monocyte‐to‐lymphocyte ratio (Figure S2C).

In some cases, the deterioration of renal function – blood urea nitrogen (BUN) and plasma creatinine correlated with COVID‐19 severity – may have limited clearance of proinflammatory markers into urine and undermined their relationship to disease severity. For example, 8,12‐*iso*‐iPF_2α_‐VI, 5‐HETE and 12‐HETE in urine were negatively correlated with BUN, creatinine and disease severity.

#### 3.2.3 Eicosanoid response to COVID‐19 seroconversion in healthy marines

Here, we addressed the hypothesis that healthy individuals undergoing seroconversion after infection with COVID‐19 might express a milder lipidomic signature of inflammation compared with that observed in patients with sepsis and that this might be reflected by mild inflammatory symptomatology. Having segregated patients based on symptomatology, we were then surprised to find that LA increased significantly after infection only in those who seroconverted asymptomatically but not in the symptomatic group (Figure S3A). Consistently, its 9(10)‐DiHOME and 11(12)‐DiHOME metabolites also increased only in the asymptomatic group and continued to increase even after patients tested negative (Figures S3B and C). Another two LA metabolites, 9‐HODE and 13‐HODE followed the same trend as the DiHOMEs, but the changes were not statistically significant (Figures S3D and E). Additionally, LA and 5(6)‐DHET were positively correlated with cycle threshold (CT) values of the qPCR test, indicating that concentrations of these two compounds were negatively correlated with viral load.

#### 3.2.4 Eicosanoids and immune cells

**FIGURE 3:**
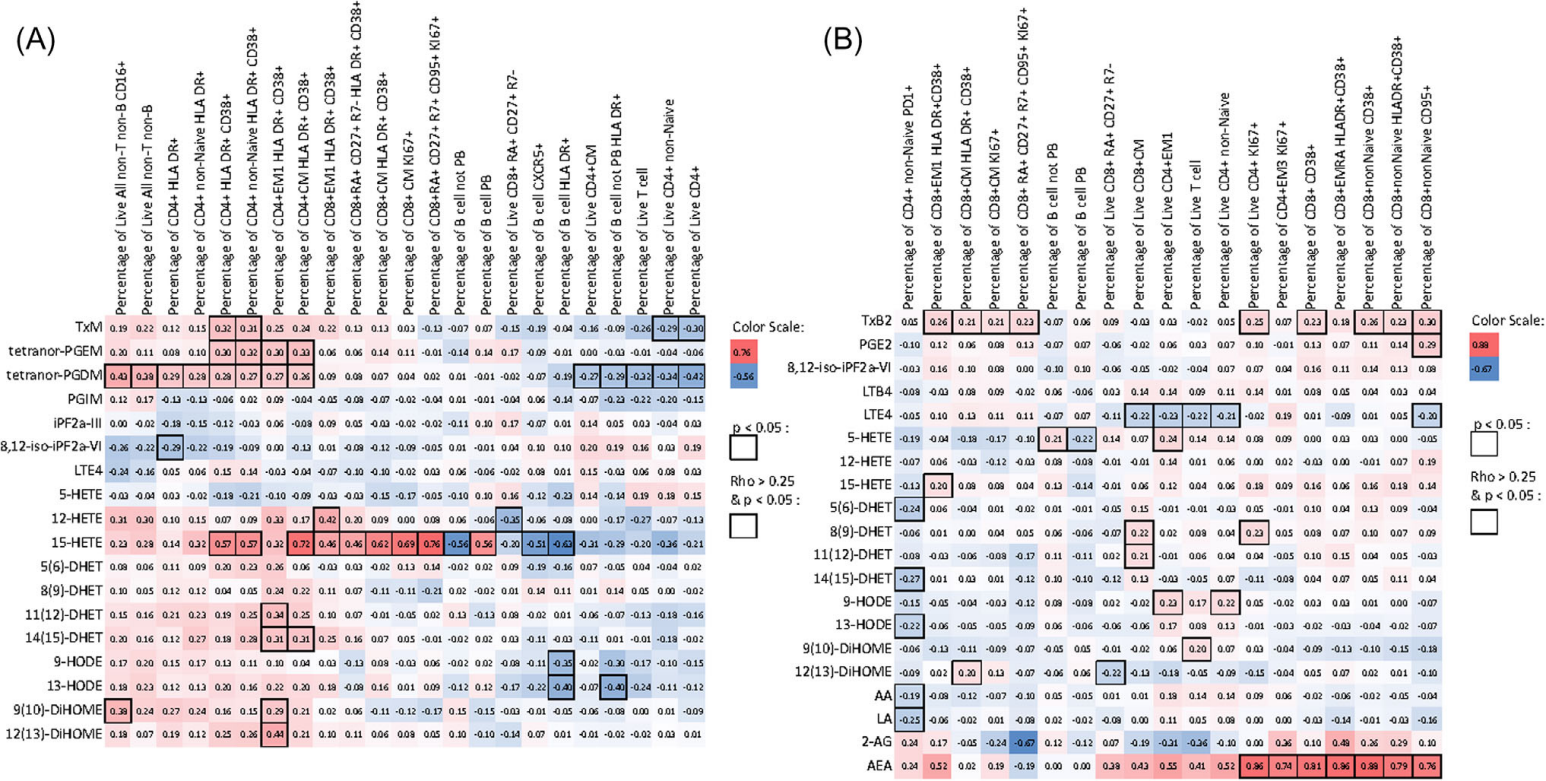
Correlation between eicosanoid concentration and the frequency of immune cell subtypes. Heat maps show the Spearman correlation coefficient (*ρ*) between eicosanoid concentrations and the frequency of immune cell subtypes in (A) urine and (B) plasma. The colour scale represents the strength and direction of the correlation, with warmer colours indicating positive correlations and cooler colours indicating negative correlations. The *p* value was calculated and marked in the heat maps.

Although lymphocytes have a low capacity to generate them, eicosanoids are potent modulators of the immune response to viral infection.^4^ The Spearman correlations (*ρ*) between immune cell types were significantly correlated with COVID‐19 severity (*q*‐value < 0.05) and eicosanoids (Figure 3). Urinary TxM, PGEM, PGDM and 15‐HETE correlated with CD4 T cells expressing CD38 and HLA‐DR markers of CD4+ T cells activation. Like urinary PGEM and TxM, urinary PGDM was correlated with CD4+ T cell activation. Its relationship with the frequency of non‐T non‐B cells was more pronounced than that of either PGEM or TxM. Urinary 15‐HETE was correlated with CD8 T cells expressing CD38, HLA‐DR and KI67. It was also positively correlated with plasmablasts and negatively correlated with non‐plasmablast B cells.

The EC AEA was correlated with activation and proliferation of T cells. For example, it correlated with activation of CD8 T cells, with the activated CD8 EMRA subset, with proliferating CD4 T cells and with expression of apoptotic markers such as Fas on non‐naïve CD8 T cells (Figure 3B).

In summary, these results are consistent with eicosanoids – specifically, PGE_2_ TxA_2_ and LTE_4_ and to a lesser extent, PGD_2_ promoting the activation of CD4+ T cells in patients with severe COVID‐19. PGD_2_, an eicosanoid that exhibits some selectivity for sepsis due to COVID‐19, appears to exhibit a particular influence on non‐B non‐T cells and CD4+ T cells while the EC, AEA is linked to CD8 T cell activation.

#### 3.2.5 PBMCs

**FIGURE 4:**
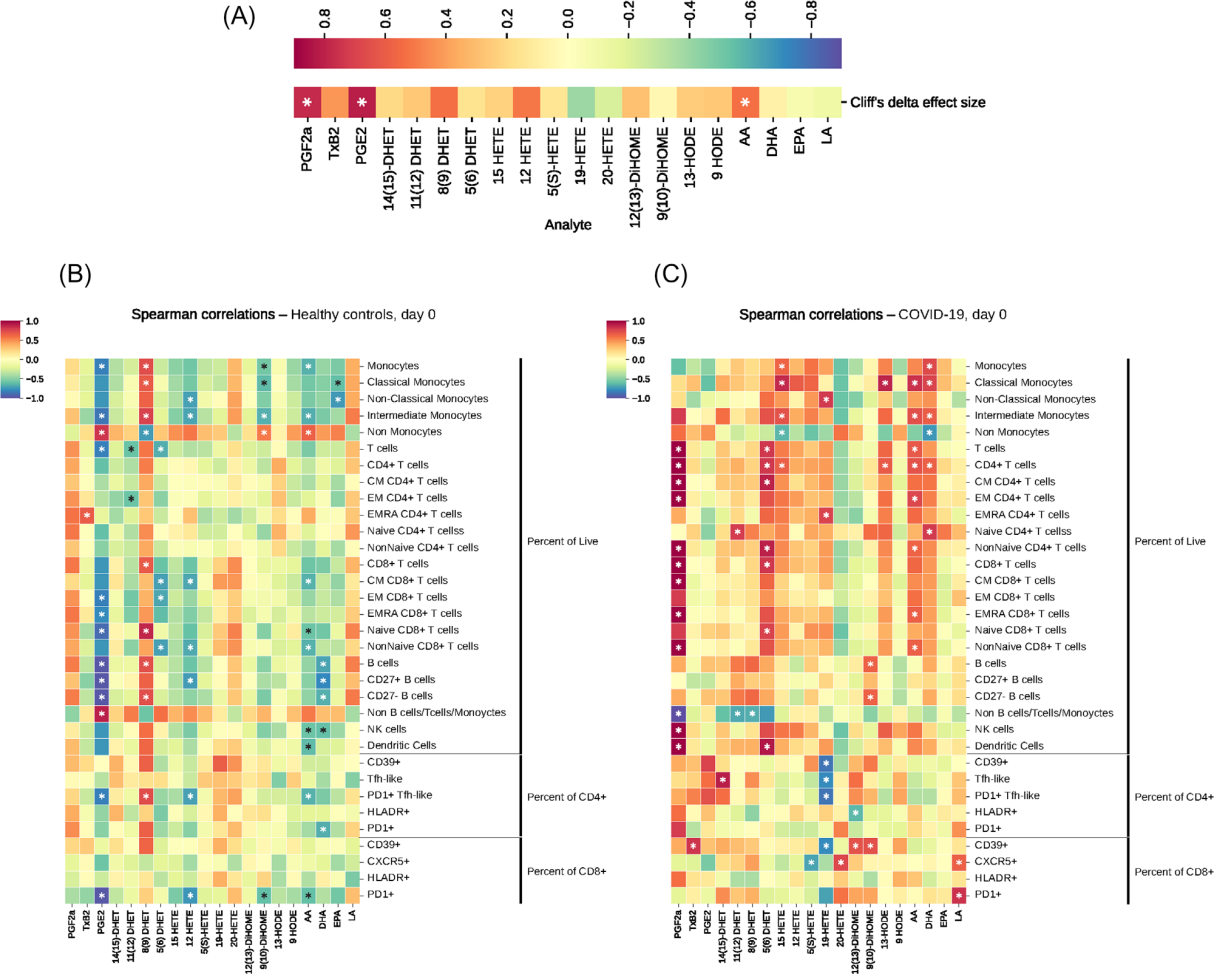
(A) Changes in the lipidomic profile of PBMC supernatant from COVID‐19 patients compared with healthy volunteers. The heat map shows the effect size of differences of levels of arachidonic acid and linoleic acid metabolites, corrected by cell number. The values within each box represent the Cliff's delta, indicating the magnitude of the differences. The significance of these differences was determined using non‐parametric Mann–Whitney tests and significant (*p* < .05) differences are marked with * in the heat map. (B and C) Correlation between eicosanoid concentrations and the frequency of immune cell subtypes in PBMCs are shown. The heat map shows Spearman's correlation coefficient (*ρ*) between lipid metabolites and the frequency of immune cell subtypes in PBMCs from healthy controls (B) and COVID‐19 patients (C). *p* Values were calculated and significant (*p* < .05) correlations are marked with * in the heat maps. The colour scale reports the Spearman's correlation coefficient *ρ*. Warmer colours indicate positive correlations, while cooler colours indicate negative correlations.

The relative differences of average concentrations, corrected by cell number, of measured metabolites between COVID‐19 patients and healthy controls are shown in Figure 4A. PBMC PGE_2_ was significantly elevated in COVID‐19 patients, consistent with our results above in urine and plasma and with other studies.^21^, ^47^ In addition, PBMC release of PGF_2α_ and AA were also modestly but significantly increased in the COVID‐19 group compared with the healthy controls.

*Correlation between lipid profile and immune features*. The Spearman ρ of the correlations between cell types and lipid metabolites are depicted in Figures 4B and C. As expected, there was a striking difference in the relationships observed between the two groups. In PBMCs from healthy controls, PGE_2_ was negatively correlated with monocytes, B cells and some subpopulations of memory T cells; 8,(9)‐DHET was positively correlated with classical monocytes, CD8+ T‐cells, follicular T helper cells and B‐cells, while it was negatively correlated with non‐monocytes (Figure 4B). In contrast, in PBMCs from COVID‐19 patients, PGF_2α_ was positively correlated with different sub‐populations of memory CD4+ and CD8+ T‐cells, dendritic and NK cells. AA and 5(6)‐DHET were positively correlated with subpopulation of CD4+ T cells. TxB_2_ and, as previously described, 9(10)‐DiHOME and 12(13)‐DiHOME^52^ were positively correlated with CD39+ CD8+T‐cells.^53^ LA was positively correlated with PD1 and CXCR5 expression in CD4+ and CD8+ T cells, suggesting a shared module in T cells associated with LA. 15‐HETE, AA, DHA and 13‐HODE were positively correlated with classical monocytes. 19‐HETE was positively correlated with non‐classical monocytes, while it was negatively correlated with Tfh cells and with CD39+ expression on CD4+ and CD8+ T‐cells (Figure 4C).

In summary, PGE_2_ and PGF_2α_ production are elevated in PBMCs from patients with COVID‐19. PGF_2α_ release correlates with broad populations of immune cells in PBMCs from patients with COVID‐19.

### 3.3 High abundance lipids

#### 3.3.1 Ether phospholipids and ChoE‐18:3 are related to severity of COVID‐19

High abundance lipidomics data were obtained from the plasma of 67 COVID‐19 patients, 66 non‐COVID patients and 16 age‐matched healthy controls at the day of enrolment (day 0) and after a week (day 7). Positive (1139 features) and negative (985 features) ionisation mode data were analysed to obtain untargeted lipid data. A supervised orthogonal partial least square discriminant analysis (OPLS‐DA) model from both datasets yielded distinct clustering of the subject groups (Figure S4). The subject groups showed similar clustering at both time‐points. We sought to determine if the lipidomic response differed between the two time‐points using a shared and unique structure analysis of the OPLS‐DA models of respective days (Figure S5). This analysis demonstrates that the differential lipid responses in positive mode lipids across the three classes are similar between the two timepoints, suggesting a week of treatment/ICU intervention was not enough to induce discernible changes in this subset of the lipidome.

Interestingly, several negative mode lipid features showed opposing trends between day 0 and day 7. We analysed the day 0 samples further and used a Kruskal–Wallis one‐way ANOVA to identify specific features that are differentially expressed across the three groups of subjects. A large fraction (∼42 and ∼26% for positive and negative modes, respectively) of the data was found to show significant variance (false discovery rate [FDR] < .2). However, major clusters of variance were associated with an increase/decrease of features in non‐COVID subjects, highlighting the importance of considering non‐COVID infected patients as one group of controls. We did observe, however, small but distinct clusters of lipids with COVID‐19 specific variation in both datasets (Figure S6).

**FIGURE 5:**
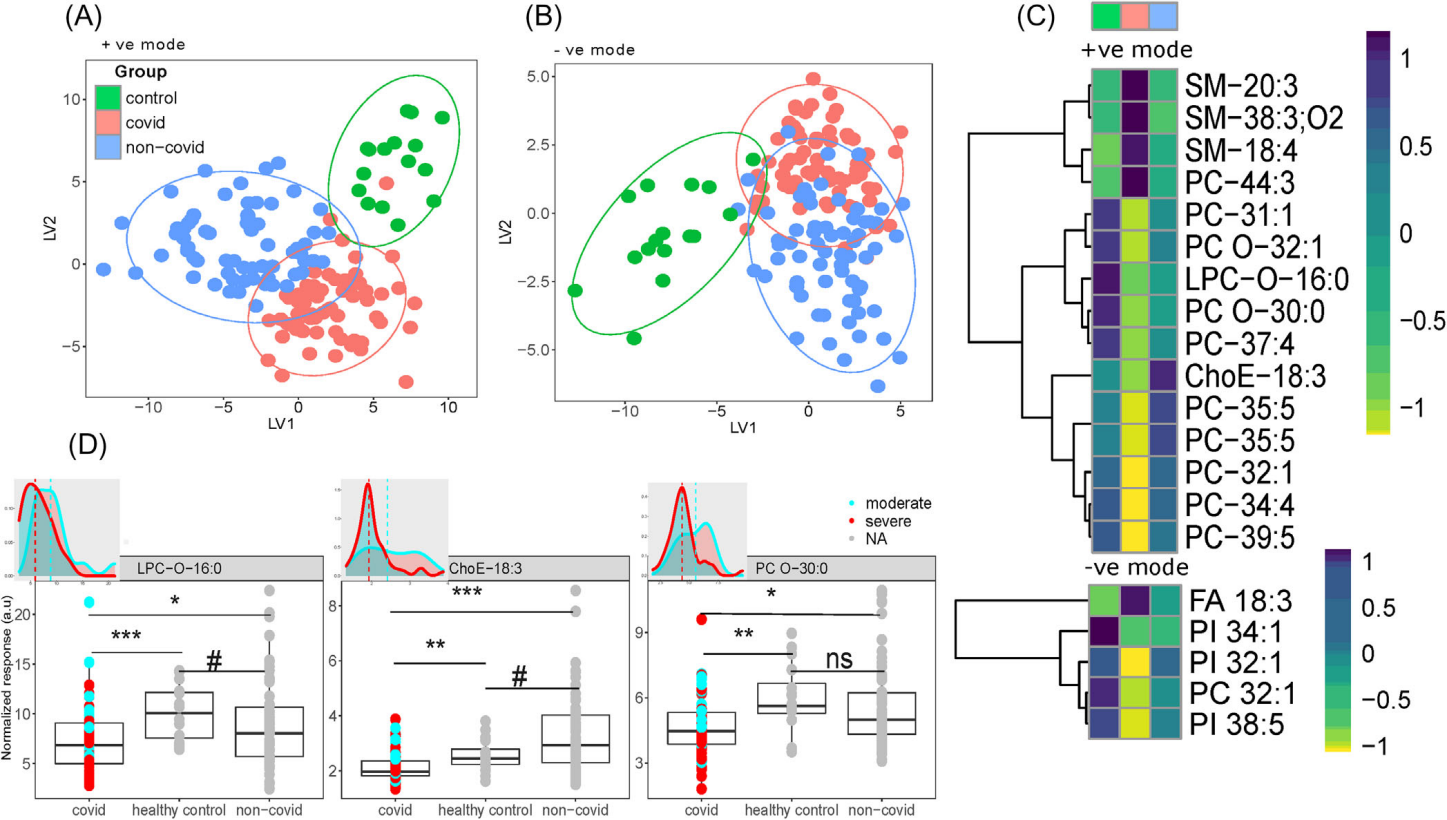
High abundance lipidomic response to COVID‐19 infection. Targeted positive and negative mode lipidomics data were assessed to evaluate signatures of COVID‐19 specific alterations. OPLS‐DA scores plot from (A) positive mode and (B) negative mode lipid data. (C) Lipids specific to COVID‐19 assessed using Kruskal–Wallis test and pairwise Mann–Whitney test (Kruskal–Wallis FDR < .2, *p* (Mann–Whitney) < .05 between COVID/non‐COVID and COVID/control; >.05 between non‐COVID/control. Median value of each lipid for each group is presented as heatmap. (D) Boxplots showing three lipids that are COVID specific as well as significantly different (FDR < 0.2) between moderate (ordinal score > 4) and severe (ordinal score < 4) COVID subjects (#*p* < .1, *p < .05, **p < .01, ***p < .001, ns = not significant). Inset density plots depict the distribution of respective lipids across the moderate and severe COVID groups. Dotted line represents the median values.

We next mapped our untargeted feature sets onto known literature reported lipids by mass/charge (*m*/*z*) values^54^, ^55^ and created targeted lipid datasets. We assigned 393 and 205 lipids from positive and negative mode datasets, respectively (Table S4). We first used OPLS‐DA analysis to identify multivariate clustering between the three groups of subjects. Both positive and negative mode data yielded significant models (*Q*2(cum) = 0.45, 0.26 for positive and negative mode data, respectively; CV‐ANOVA *p* < .0001 for both) and showed significant clustering between the three groups (Figures 5A and B). Using a Kruskal–Wallis test, we found 15 positive mode lipids that were significantly altered in COVID‐19 patients (FDR < 0.05, pairwise *p* values – COVID‐19 vs. non‐COVID‐19 sepsis patients < 0.05, COVID‐19 vs. healthy control < 0.05, healthy control vs. non‐COVID‐19 sepsis patients > 0.05; Figures 5C and S7). Using the same FDR and *p* value cutoffs for the negative mode data, we found six features belonging to five lipids that were specifically altered in COVID subjects (Figures 5C and S8, two features were annotated as FA 18:3). Sphingomyelin levels were elevated in COVID‐19 patients with sepsis compared with both non‐COVID‐19 sepsis patients and healthy controls, in agreement with previous observations,^18^ while most phospholipids (phosphatidylcholines, phosphatidylinositols, ether phospholipids) were decreased. Interestingly, cholesteryl ester 18:3 (ChoE‐18:3) was decreased in COVID‐19 patients while the corresponding fatty acid was elevated. We then sought to determine which of these lipid signatures was also a function of COVID‐19 severity by mapping ordinal scores to subjects as severe (ordinal score 5−7) and moderate (<1–4) groups and compared the lipid levels using nonparametric tests. Three lipids, ChoE‐18:3, LPC‐O‐16:0 and PC‐O‐30:0 significantly varied between the moderate and severe COVID‐19 groups (FDR < .2; Figure 5D).

#### 3.3.2 Ether lipids are related to inflammatory pathways

To investigate the pathophysiological relevance of the observed lipids in the context of COVID‐19 infection, we extracted the immunotypes, proteins, eicosanoids and clinical parameters that significantly correlated (Spearman's rho < −0.4 or >0.4, *p* < .05) with the level of these three lipid species in the COVID‐19 patients (Table S5). LPC‐O‐16:0 was significantly correlated with measures of inflammation (PCT, CRP) and clinical severity of COVID‐19 (ordinal score at day 7; the ordinal score at enrolment and day 7). Interestingly, sPLA_2_ was also significantly negatively correlated with LPC‐O‐16:0. In addition, several (17 each) immune response features and proteins were associated with the circulatory levels of LPC‐O‐16:0. For example, this lipid is significantly negatively correlated with the previously described immunotype 2 in COVID‐19.^35^

**FIGURE 6:**
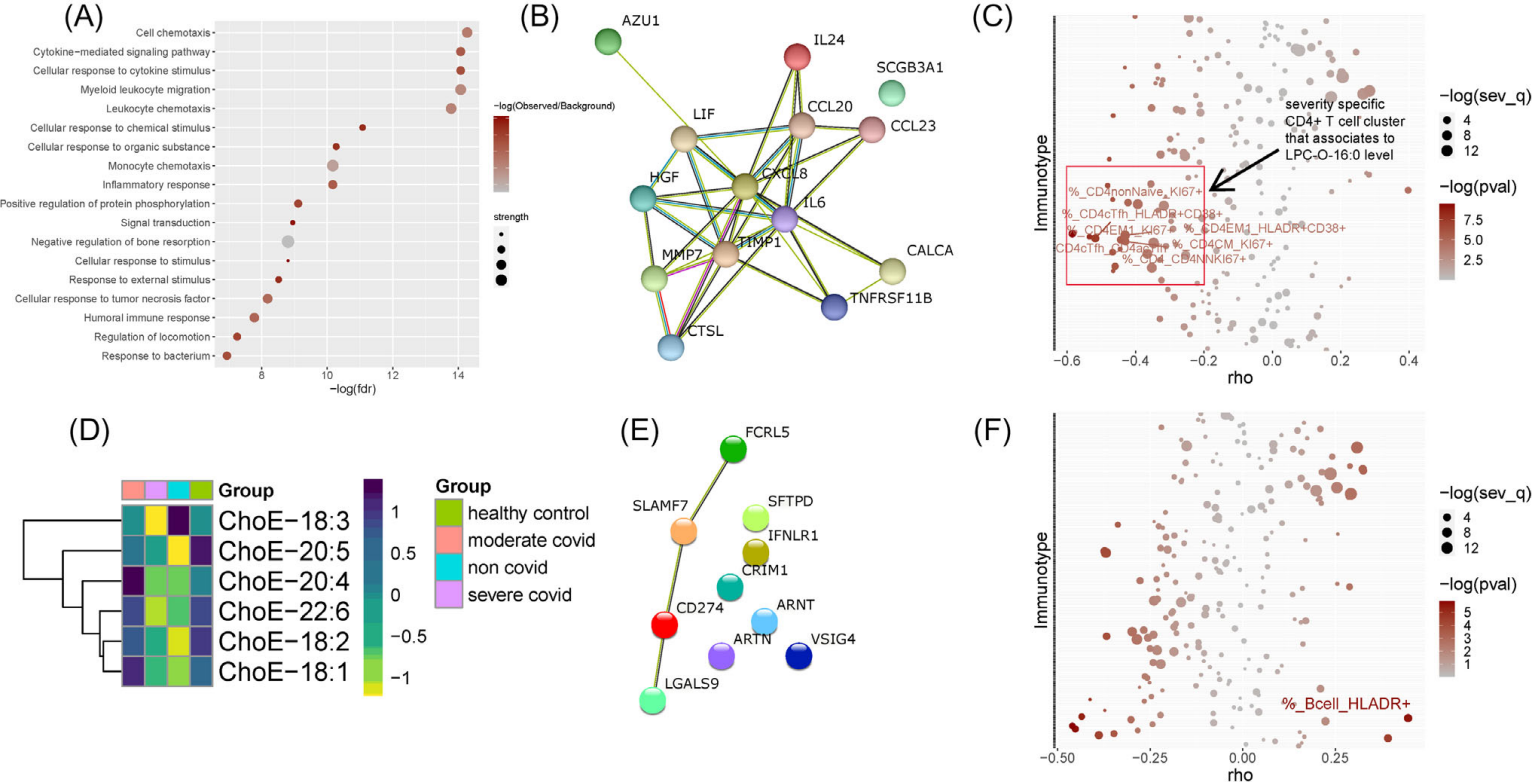
Highly abundant lipids restrain inflammation. LPC‐O‐16:0 is associated with the inflammatory response (A–C) and ChoE‐18:3 is specific to severe COVID (D–F). Levels of LPC‐O‐16:0 from COVID subjects were used for correlation analysis with protein and immunotype data to identify biological pathways associated with the lipid. (A) Top functionally enriched pathways (FDR < 0.001, for all significantly enriched pathways (FDR < 0.05), see Table S6) associated with the protein sets that are significantly associated with LPC‐O‐16:0 (Spearman's rho < −0.4 and *p* < .05) and vary with COVID severity (FDR < 0.05). The proteins are listed in Table S5. (B) Functional enrichment network of proteins (described in Table S5) significantly associated to LPC‐O‐16:0 (Spearman's rho < −0.4 and *p* < .05). (C) Immune cell types significantly correlated to LPC‐O‐16:0 (Spearman's rho < −0.4 or >0.4 and *p* < .05). The points are coloured by −log (*p* value of correlation) and sized by −log (*q* value between moderate and severe COVID (*q*_sev)). (D) ChoE‐18:3 is the only cholesteryl ester molecule that is depleted in the severe COVID group compared with moderate COVID, but elevated in the non‐COVID subjects. (E) Functional enrichment network of proteins significantly associated (Spearman's rho < −0.4 or >0.4 and *p* < .05) to ChoE‐18:3. (F) ChoE‐18:3 is significantly, and exclusively, associated to various B cell populations (Spearman's rho < −.4 or >.4 and *p* < .05).

PC‐O‐30:0 was associated with 15 proteins and 11 immunotypes, but only to one clinical parameter, CRP (Table S5). It is significantly negatively correlated with the previously described immunotype 1 in COVID‐19.^35^ Comparison of the proteins associated with the two lipids revealed largely orthogonal sets, suggesting that they are likely associated with different biological pathways. We performed functional protein enrichment analysis using string‐db (Table S6 and Figure S9) to interrogate further the degree of overlap. Several enriched pathways were significantly associated with LPC‐O‐16:0 (FDR < .001; Figures 6A and B) resulting in a strongly interconnected network, centred around the protein CXCL8 (Figure 6B). Such a well‐defined network was not found from the proteins associated with PC‐O‐30:0 (Figure S10). Thus, amongst the two ether lipids, LPC‐O‐16:0 is more closely related to the known disease pathophysiology.

Among the top five enriched pathways, two broad categories were apparent – cellular chemotaxis and cytokine signalling pathways. To identify the most probable tissue locations enriched by the proteins associated with LPC‐O‐16:0, we investigated the tissue expression enrichment of the string‐db output (Figure S11 and Table S7). Nine tissues were found to be enriched significantly (FDR < 0.05), with neutrophils being the most prominent. Given that the proteins are all negatively associated with LPC‐O‐16:0 that decreases with severity, it is plausible that this lipid is related to the inflammatory response towards the infection that is mediated by leucocyte chemotaxis and associated cytokine stimulation.

We identified features of CD4+ T cells (Figure 6C) that are both significantly associated with LPC‐O‐16:0 and significantly differ as a function of disease severity (FDR < 0.05). Activation or proliferation of CD4+ T cell subsets, including memory and T helper follicular cells inversely correlate with LPC‐O‐16:0, again consistent with a role as a constraint on the inflammatory response to a severe COVID‐19 infection, as previously suggested.^55^ Moreover, this species and ether‐LPCs in general have been shown by others to be downregulated in sepsis, not just in severe COVID‐19.^25^ Correspondingly, we found LPC‐O‐16:0 to be negatively correlated with sPLA_2_.

#### 3.3.3 ChoE‐18:3 is specifically related to severe COVID‐19

Cholesteryl ester‐18:3 (ChoE‐18:3) was strikingly depleted in severe COVID‐19 infection (Figure 6D). It was decreased compared with controls and as a function of severity in COVID‐19; however, unlike the ether lipids, it is elevated in the non‐COVID‐19 sepsis patients, compared with COVID‐19 patients with severe disease. Interestingly, none of the other ChoE molecules that we profiled showed a similar pattern (Figures S12 and 6D). We examined associated proteins, peripheral immune cell responses and clinical parameters to identify the biological and pathophysiological relevance context of this lipid (Spearman's *ρ* < −.4 or >.4, *p* < .05; Table S5). ChoE‐18:3 was significantly associated with only 10 proteins (Table S5) and, by implication, a limited number of pathways (Figure 6E). Functional enrichment analysis, however, revealed several significant pathways (Table S6), the majority of which was related to regulation of immune response, with negative regulation of T cell proliferation being the most significant.

We next sought cellular immune responses associated with the cholesteryl esters. Interestingly, only five B cell populations were correlated with ChoE‐18:3 (Table S5; Spearman's rho < −.4 or >.4 and *p* < .05). Such exclusive association with B cells was not observed with the other cholesteryl ester species. HLADR+ B cells were positively correlated, while CD39/CD138/KI67 positive B cells were negatively correlated with ChoE‐18:3 (Table S5). Among the B cell species significantly associated to ChoE‐18:3, HLADR+ cells were also significantly different between moderate and severe COVID‐19 patients (Figure 6F). As such, HLADR+ B cells showed positive association with two cholesteryl ester species – ChoE‐18:3 and ChoE‐22:6 (Figure S13), each arising from fatty acids (FA 18:3–LA and FA 22:6–DHA) that bear precursor‐product relationships. HLADR+ B cells are reduced in patients with severe COVID‐19^56^ compared with healthy controls and patients with mild COVID independent of dexamethasone treatment. ChoE‐18:3 is a potential source of the elevated ALA (alpha‐linolenic acid) and its downstream products, EPA and DHA that we observed in COVID patients and was also negatively correlated with PLA_2_G2D (Figure S14; Spearman's *ρ* = −.55, *p* < .05), a source of inflammatory eicosanoids as described above.

### 3.4 Integrative correlation analysis

**FIGURE 7:**
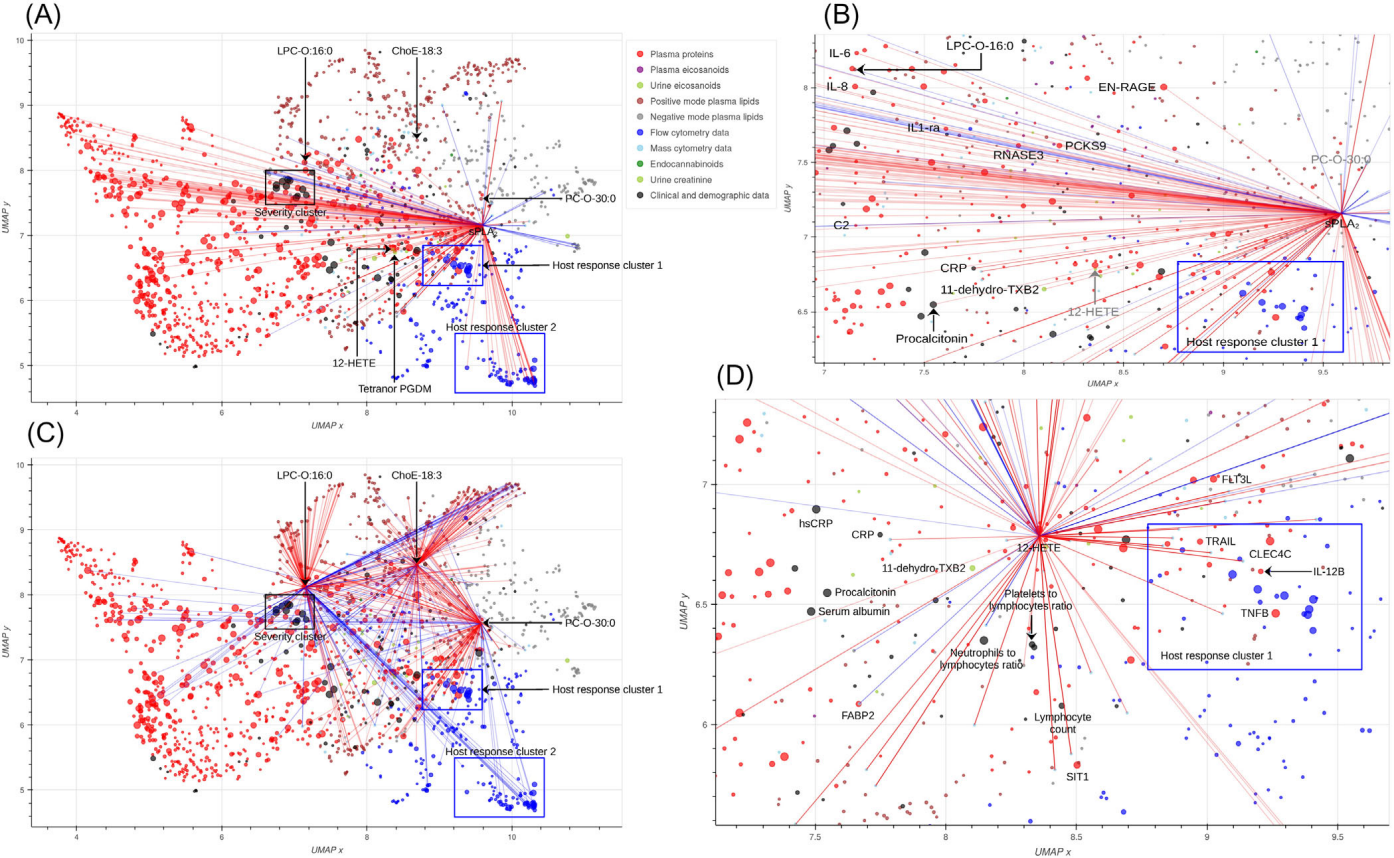
Visualisation of COVID‐19 correlation network. (A) UMAP projection of features measured in COVID‐19 cohort. Each feature is represented by a circle whose size is negatively proportional to log10 of the *p* value testing difference of the feature in moderate versus severe COVID‐19. Hence, bigger circles correspond to features that differ more significantly between moderate and severe cases of COVID‐19. Correlations of sPLA2 with other features are represented by lines, red lines correspond to positive correlations, with Spearman's correlation coefficient > 0.4, blue lines correspond to negative correlations, with Spearman's correlation coefficient < −0.4. Named features are further discussed in this paper. (B) Region in close proximity to host response cluster 1 containing sPLA_2_. Features printed in black show significant (*p* < .05) correlation with sPLA2, either with Spearman's correlation coefficient > 0.4 (red lines) or <−0.4 (blue lines). (C) Correlation subnetwork for three high abundance lipids of interest. Red lines denote pairs of features with Spearman's correlation coefficient >0.4, blue lines denote pairs of features with Spearman's correlation coefficient < −0.4. (D) Region in close proximity to host response cluster 1 containing urinary 12‐HETE and several other urinary eicosanoids. Correlations of 12‐HETE with other features are represented by red (Spearman's correlation coefficient > 0.4) or blue (Spearman's correlation coefficient < −0.4) lines. Compared with sPLA2, 12‐HETE exhibits lower number of strong correlations with other features. However, the positioning of 12‐HETE in the network is based on all correlations among all pairs of features, so its proximity to other features, such as IL‐12B, TNFB and SIT1 could be of interest.

*COVID‐19 severity correlation network*. The UMAP projection of COVID‐19 correlation network is shown in Figure 7A. Host response cluster 1 in this analysis contains the feature defining immunotype 1, associated with disease severity and mortality as previously described in COVID‐19.^35^ Most features in our host response cluster 2 are highly positively correlated with the feature defining immunotype 2 in that publication.^35^ The actual defining feature is in the upper left corner of the boundary rectangle of the cluster. Our severity cluster includes severity scores such as APACHE III and the COVID‐19 severity ordinal scale scores (which were evaluated four times throughout the month after hospitalisation), length of hospital stay and indication of survival within 1 and 3 months after hospital admission.

Amongst the lipids mentioned previously, PC‐O‐30:0 is negatively correlated with several features in host response cluster 2 and is positioned relatively close to host response cluster 1 (Figures 7A and C). Like PC‐O‐30:0, LPC‐O‐16:0 is negatively correlated with host response cluster 1, while ChoE‐18:3 is negatively correlated with a third, B cell related, cluster of immune response related features positioned left of the second one (Figure 7C). Urinary 12‐HETE is located close to host response cluster 1 (Figures 7A and D) and so are several immune response related proteins, many of which differ significantly in their abundance in moderate versus severe COVID‐19 (such features are represented by larger circles in the figure), including IL‐12B, CLEC4C, FLT3L, TRAIL and TNFB. 12‐HETE is also close to SIT1, which interacts with the angiotensin‐converting enzyme 2 (ACE2) receptor for SARS‐CoV‐2.^57^ Lymphocyte count, NLR and PLR are clinical immune related features located below 12‐HETE in the region shown in Figure 7D. Many immune response related proteins correlate significantly with sPLA_2_ (Figure 7B).

*Non‐COVID sepsis control versus COVID‐19 differential correlation network*. The UMAP projection of non‐COVID control versus COVID‐19 differential correlation network is shown in Figure S15A. 12‐HETE in plasma exhibited changes in correlation with multiple features (Figure S15A). STRING enrichment analysis^51^ of 87 proteins differentially correlated with 12‐HETE was performed (Figure S15B). The top two KEGG pathways, as ranked by FDR, were *Viral Protein Interaction with Cytokines and Cytokine Receptor* (FDR adjusted *p* value 8.57 × 10^−12^) and *Cytokine‐Cytokine Receptor Interaction* (FDR adjusted *p* value 2.12 × 10^−18^). These 87 proteins generally exhibited loss of correlation with 12‐HETE in the COVID‐19 cohort (Figure S15C). Analysis of the undirected differential correlation graph placed 12‐HETE in the top 5% of features in terms of degree centrality and betweenness centrality. Proteins in the top 5% of features ranked by degree centrality (52 proteins in total) were enriched in the Neutrophil Degranulation Reactome Pathway (FDR adjusted *p* value .00025).

## 4 DISCUSSION

Viruses perturb the cellular lipidome, which is, in turn, crucial to their replication and propagation.^1^, ^2^, ^5^, ^57^, ^59^, ^60^ Aside from these direct effects, lipids modulate powerfully the immune response to viral infection that restricts its consequences or, if unrestrained, may amplify the insult to the infected host.^4^ Here, we interrogated this interaction across the spectrum of severity of infection with COVID‐19 with a particular focus on the less abundant, but biologically potent eicosanoid products of AA and LA. Indeed, the spike (S) glycoprotein on the surface of SARS‐CoV‐2 tightly binds LA, stabilising it and reducing its interaction with the host ACE2 receptor that facilitates viral cell entry.^60^

This study contrasts with other investigations of the lipidomic response to COVID‐19 (i) by including both high and low abundance lipids; (ii) by comparing patients with sepsis due to COVID‐19 with patients suffering from sepsis due to other viruses and to bacteria, as well as with healthy volunteers, (iii) by integrating lipidomics with the proteome and the peripheral cellular immune response, allowing for comparisons over time from hospital admission and with the severity of the clinical response, (iv) by tracking changes in the lipidome in response to mild infections resulting in seroconversion in healthy volunteers and (v) by providing a derived inflammatory connectome as a tool to the community for further hypothesis generation.

Eicosanoids are autacoids, not circulating hormones, but are detectable in plasma or urine, reflecting endogenous biosynthesis. They are cleared rapidly from plasma to urine where primary products or metabolites are acquired non‐invasively and in greater abundance than in plasma. The cellular capacity to form eicosanoids exceeds their endogenous production in vivo.^61^

Here we find that sepsis, of bacterial or viral origin, is accompanied by an eicosanoid storm with elevated levels of COX (PGE_2_, PGD_2_, TxA_2_), LOX (12‐HETE and LTE_4_) and EPOX products (11(12)–14(15)‐DHETs) of AA, the ECs AEA and 2‐AG, along with the LA‐derived DiHOMEs. Furthermore, elements of this storm reflect disease severity and are prognostic indicators in patients with sepsis due to COVID‐19. This storm is accompanied by an increase in sPLA_2_ that metabolises the AA and LA substrates to generate these bioactive lipids. Indeed, sPLA_2_ acts as a prominent immune‐lipidomic hub, discriminates between moderate and severe sepsis in COVID‐19 and links eicosanoids to multiple inflammatory proteins and to clinical markers of disease severity. While sPLA_2_ has been previously reported as a biomarker of sepsis,^25^, ^62^ here we expand this observation mechanistically to link sPLA_2_ to the breadth of its discrete eicosanoid products, the immune cellular responses that they influence and the consequent indices of disease severity. Interestingly, we have previously described sPLA_2_ catalysed transcellular eicosanoid metabolism in the setting of platelet activation^63^ and this may also be pertinent to COVID‐19.^64^, ^65^

In our study, the patients with sepsis due to causes other than COVID‐19 had, on average, more severe disease than those with COVID‐19. In the main, eicosanoids (TxA_2_, PGE_2_, AEA, 2‐AG, DHETs and DiHOMEs) reflected comparative disease severity, but elevations in PGD_2_ were more striking in COVID‐19 despite less severe disease, reflecting a relative selectivity for SARS‐CoV‐2. Interestingly, bacterial, rather than viral sepsis evoked the more integrative eicosanoid response across the COX, LOX and EPOX pathways.

PGD_2_ is a potent immune modulator, acting via the D Prostanoid receptors, DPr1 and DPr2 . DPr1 signalling delays migration of DCs to the lung and lymph nodes via downregulation of the chemokine CCR7.^66^ PGD_2_ also contributes to the pathogenesis of respiratory syncytial virus (RSV) bronchiolitis and susceptibility to asthma via DPr2 signalling.^66^ In a neonatal model of severe RSV bronchiolitis, treatment with a DPr2 inhibitor decreased viral load and improved morbidity via upregulation of IFN‐λ. This effect was recapitulated by treatment with a DPr1 agonist, suggesting that these two receptors for PGD_2_ have opposing roles in the regulation of the antiviral response.^67^ Strikingly, middle‐aged mice lacking expression of DPr1 or PLA_2_G2D are protected from severe disease in a model of SARS‐CoV‐2 infection and a DPr1 antagonist, asapiprant, protected aged mice from lethal infection.^68^ Here, PLA2G2D was not elevated in patients with sepsis compared with healthy controls, suggesting that sPLA_2_ is the dominant driver of PGD_2_ formation in humans with COVID‐19.

Increased biosynthesis of PGE_2_ is a feature of sepsis and its role as a pro‐inflammatory mediator is well established.^4^ It can activate any of 4 EPrs that differentially regulate platelet and immune cell function. Elevated levels of PGE_2_ formation have been reported by others in patients with sepsis from COVID‐19 compared with healthy controls, but here we see no distinction from the increase observed in patients with sepsis from other causes. As previously observed, PGE_2_ production by PBMCs from COVID‐19 patients is elevated compared with healthy controls. In addition, PGF_2α_ was significantly increased in the supernatant of PBMCs from COVID‐19 patients and its level correlated with broad populations of immune cells. This is of interest as deletion of the FPr restrains the pulmonary fibrotic response to bleomycin in mice^69^ and increased biosynthesis of PGF_2α_ might contribute to the pulmonary fibrosis that can complicate long COVID‐19.^70^ Interestingly, Bohnacker et al.^24^ reported that PGF_2α_ levels remain elevated in monocyte‐derived macrophages from COVID‐19 patients up to 3−5 months post infection. We observed a range of other eicosanoid – immune cell relationships in PBMCs from patients with COVID‐19.

Our extensive analysis of highly abundant lipids revealed three ether lipids that were differentially altered in the sepsis patients due to COVID‐19. All three – ChoE‐18:3, LPC‐O‐16:0 and PC‐O‐30:0 – were significantly lower in patients with sepsis from COVID‐19 than sepsis from other causes and, indeed, from healthy controls. All three discriminated moderate from severe COVID‐19. LPC‐O‐16:0 is also depleted in patients with non‐COVID‐19 sepsis relative to healthy controls. Negative correlation of these lipids with PLA_2_s and other lipid and protein markers of the inflammatory response infer their importance as restraints on inflammation.

Previous immune profiling of patients from the MESSI cohort with COVID‐19 has identified three distinct immunotypes.^35^ The first was associated with disease severity and showed robustly activated CD4 T cells, a paucity of circulating follicular helper cells, activated CD8 'EMRAs', hyperactivated or exhausted CD8 T cells and plasmablasts. Immunotype 2 was characterised by less CD4 T cell activation, Tbet+ effector CD4 and CD8 T cells, and proliferating memory B cells and was not associated with disease severity. Immunotype 3, which negatively correlated with disease severity and lacked obvious activated T and B cell responses, was also identified. Here, interrogation of the connectome reveals significant correlations between both PC O:30‐0 and LPC‐O‐16:0 with host response cluster 2 and between ChoE‐18:3 and the third host response cluster.

We anticipated that a milder lipidomic inflammatory phenotype might accompany seroconversion after infection by SARS‐CoV‐2 in healthy marines, perhaps most evident in those with accompanying symptoms. Such a mild lipidomic response was, indeed, evident. There was an elevation of LA and its DiHOME metabolites (but not of AA‐derived eicosanoids) as we had seen in patients with sepsis, but surprisingly, only in the marines who seroconverted asymptomatically.

These studies deepen our understanding of how the lipidome drives the immune response across the spectrum of severity of sepsis; the key findings are summarised in the graphical abstract. Besides expanding the breath of the eicosanoid storm that characterises sepsis, we identify immuno‐lipidic hubs that promote inflammation – sPLA_2_, PGD_2_ and 12‐HETE – and exhibit relative specificity for COVID‐19. Additionally, we identify hubs amongst the more abundant lipids, ChoE‐18:3, LPC‐O‐16:0 and PC‐O‐30:0, that restrain inflammation. These abundant lipids are depressed in patients with sepsis due to COVID‐19 compared with those with sepsis from other causes and with healthy controls. Finally, we provide a network analysis tool that displays the topical relationship of these hubs with previously described COVID‐19 immunotypes and allows for interrogation by the community to generate novel mechanistic and therapeutic hypotheses.

## CONFLICT OF INTEREST STATEMENT

N. J. M. reports funding to her institution for unrelated work from Quantum Leap Healthcare Collaborative and BioMarck, Inc, and consulting fees from AstraZeneca Inc and Endpoint Health Inc. G. A. F. is the McNeil Professor of Translational Medicine and Therapeutics and held a Merit Award from the American Heart Association. He is a senior advisor to Calico Laboratories and serves on the scientific advisory boards of Bicycle Therapeutics and Kira Pharmaceuticals. A. G. L. is a military service member. This work was prepared as part of his official duties. Title 17, US Code §105 provides that copyright protection under this title is not available for any work of the US Government. Title 17, US code §101 defines a US Government work as a work prepared by a military service member or employee of the US Government as part of that person's official duties. The views expressed in the article are those of the authors and do not necessarily express the official policy and position of the US Navy, the Department of Defense, the US Government or the institutions affiliated with the authors. S. C. S. serves as Chief Scientific Officer for and holds equity in GNOMX Corp and has been a paid speaker for Illumina.

## FUNDING INFORMATION

This work was supported by grants from the NIH U54TR001878 (G. A. F.), AI105343, AI082630, AI108545, AI155577, AI149680, U19AI082630 (E. J. W.), HL142981 and NR018836‐01 (A. M. W.) and HL161196, HL155804 (N. J. M.). Additional support was provided from Defense Advanced Research Projects Agency contract number N6600119C4022 (S. C. S.) and Defense Health Agency grant 9700130 through the Naval Medical Research Center (A. G. L.). D. M. and E. J. W. are supported by the Parker Institute for Immunotherapy. E. J. W. is also supported by the Perelman School of Medicine COVID Fund.

## Supporting information

Supporting Information

Supporting Information

Supporting Information

Supporting Information

Supporting Information

Supporting Information

Supporting Information

Supporting Information

Supporting Information

Supporting Information

Supporting Information

Supporting Information

Supporting Information

Supporting Information

Supporting Information

Supporting Information

## Data Availability

Data are available on request from corresponding author upon reasonable request.
