# Supplementary figures and images for: Deep phenotyping of the lipidomic response in COVID‐19 and non‐COVID‐19 sepsis
Source: Clin Transl Med. 2023 Nov 10;13(11):e1440. doi: 10.1002/ctm2.1440 (PMC10637636; doi:10.1002/ctm2.1440)

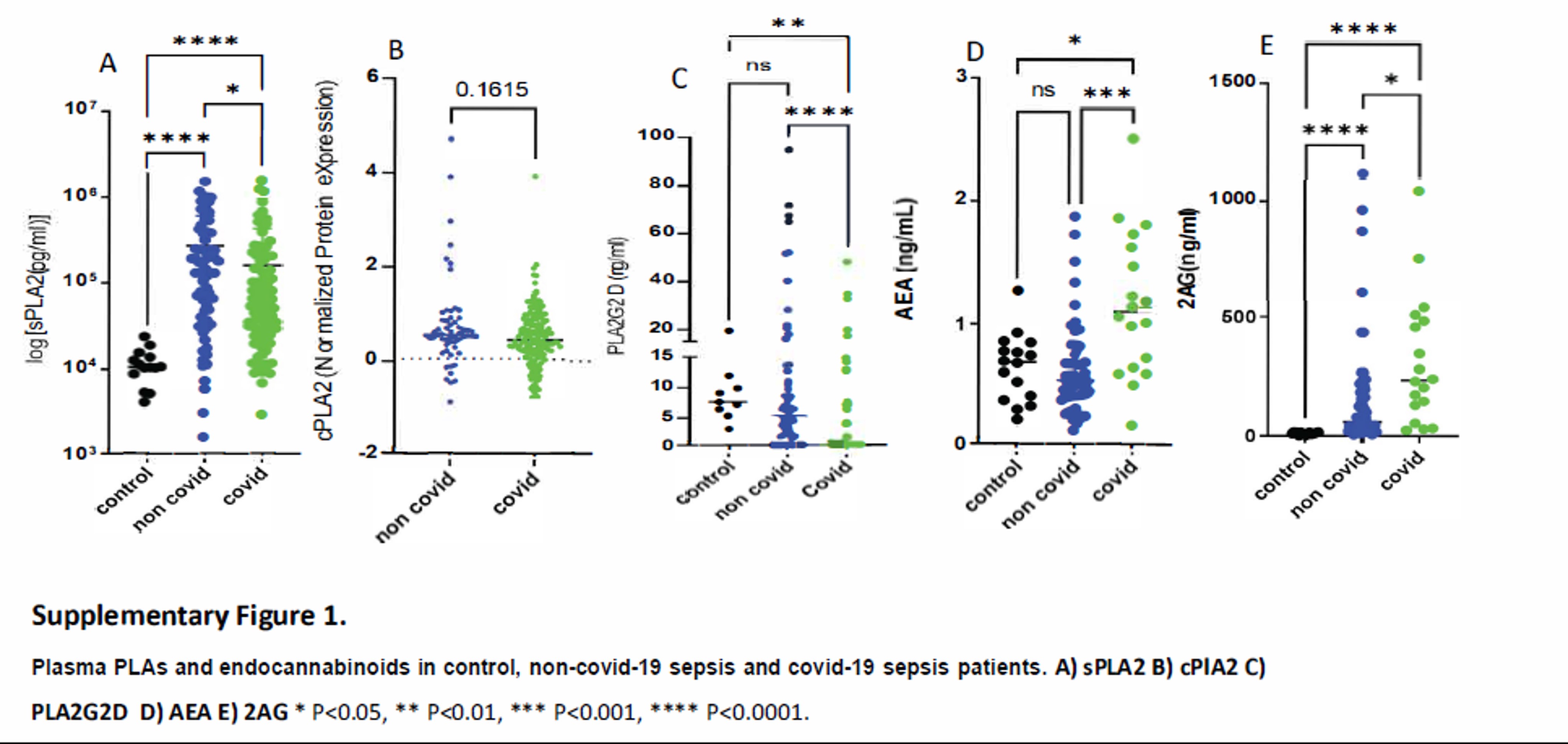

Supplement: Supplementary file 1 — Supporting Information [file CTM2-13-e1440-s015.jpg]

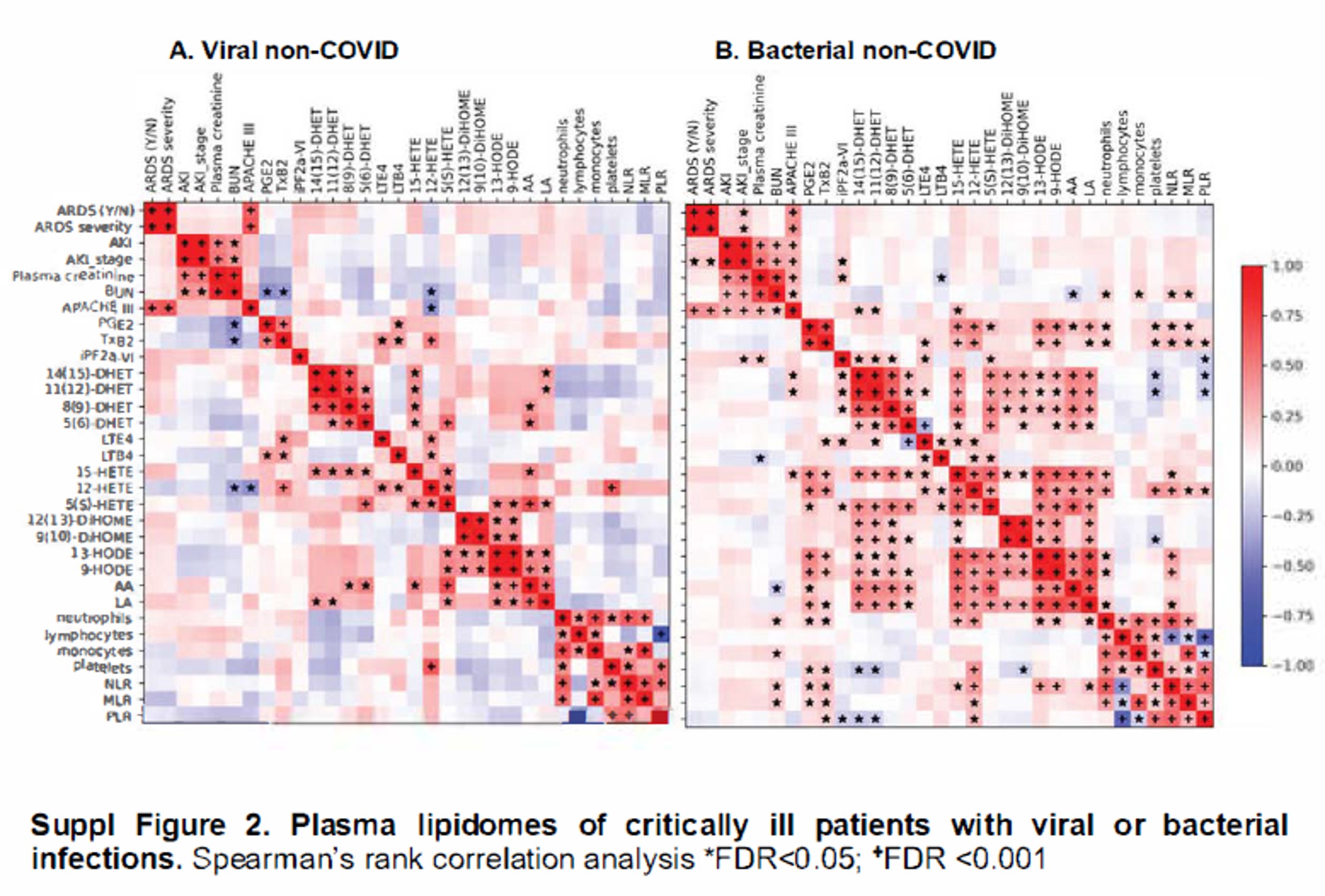

Supplement: Supplementary file 2 — Supporting Information [file CTM2-13-e1440-s012.jpg]

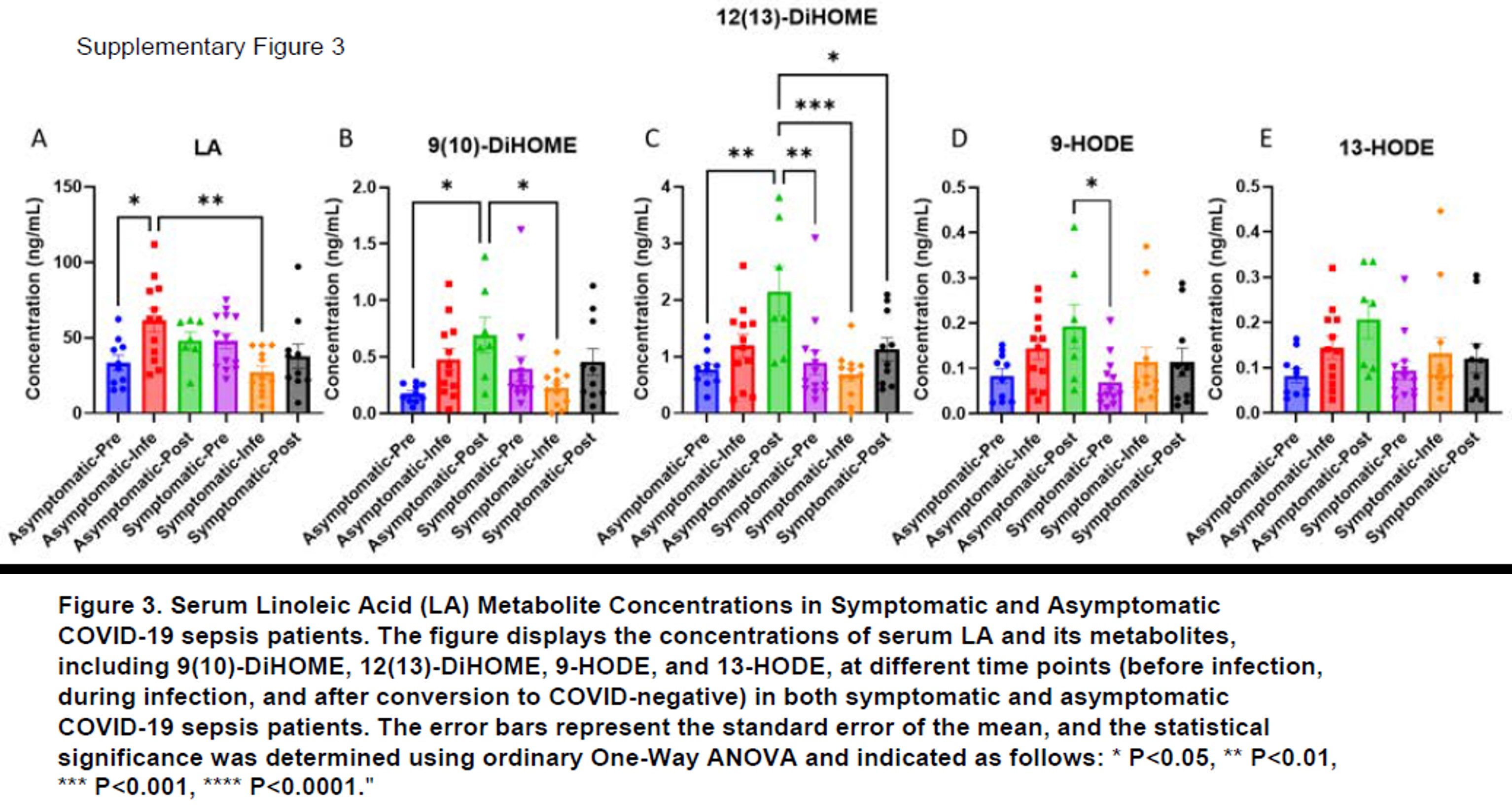

Supplement: Supplementary file 3 — Supporting Information [file CTM2-13-e1440-s016.jpg]

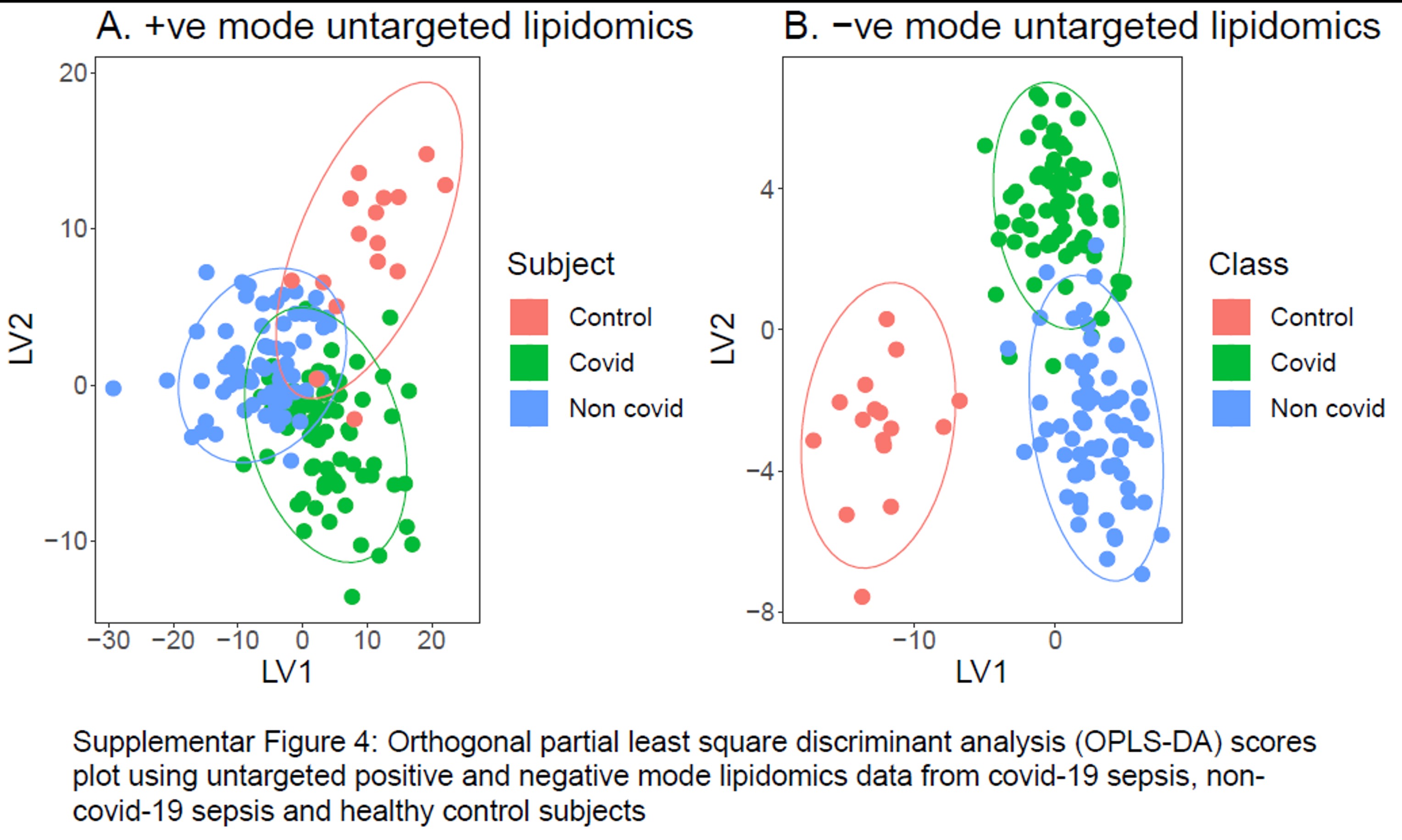

Supplement: Supplementary file 4 — Supporting Information [file CTM2-13-e1440-s010.jpg]

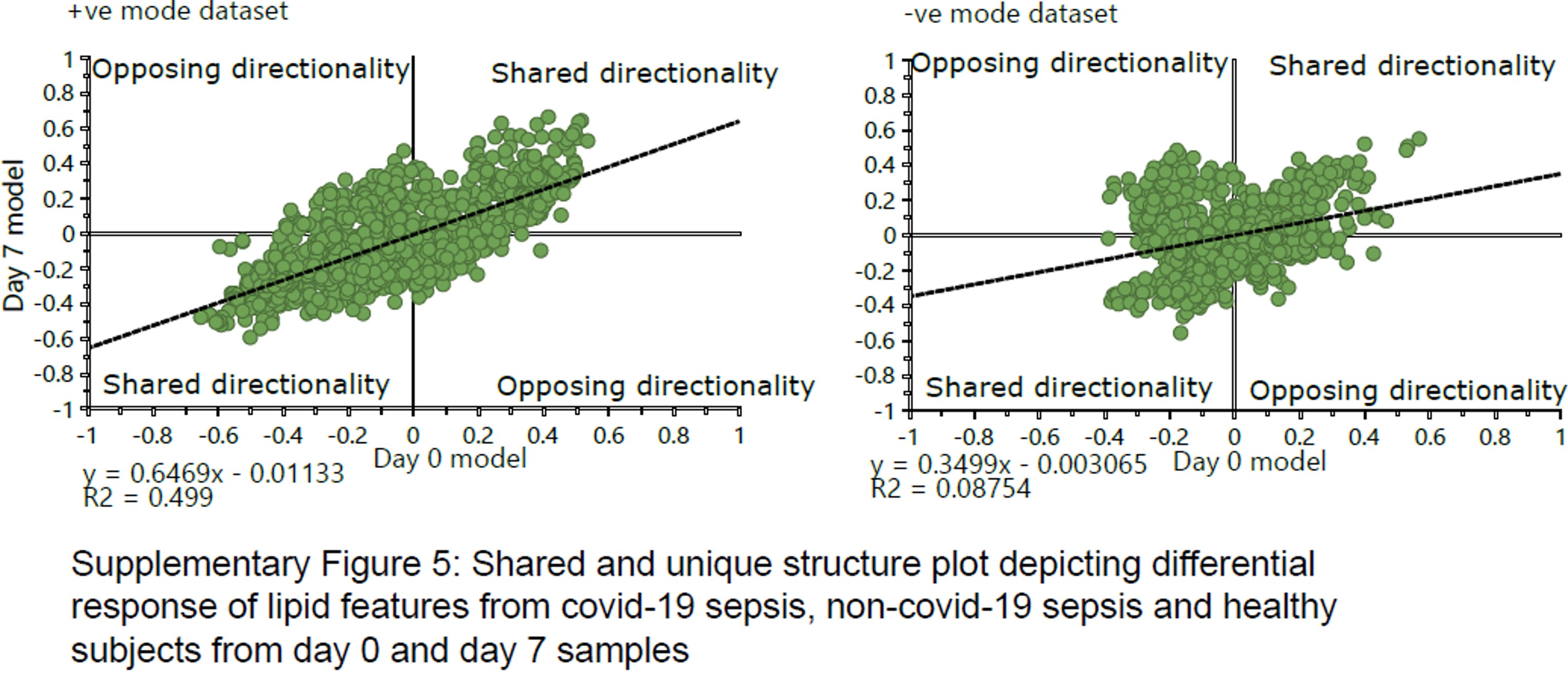

Supplement: Supplementary file 5 — Supporting Information [file CTM2-13-e1440-s013.jpg]

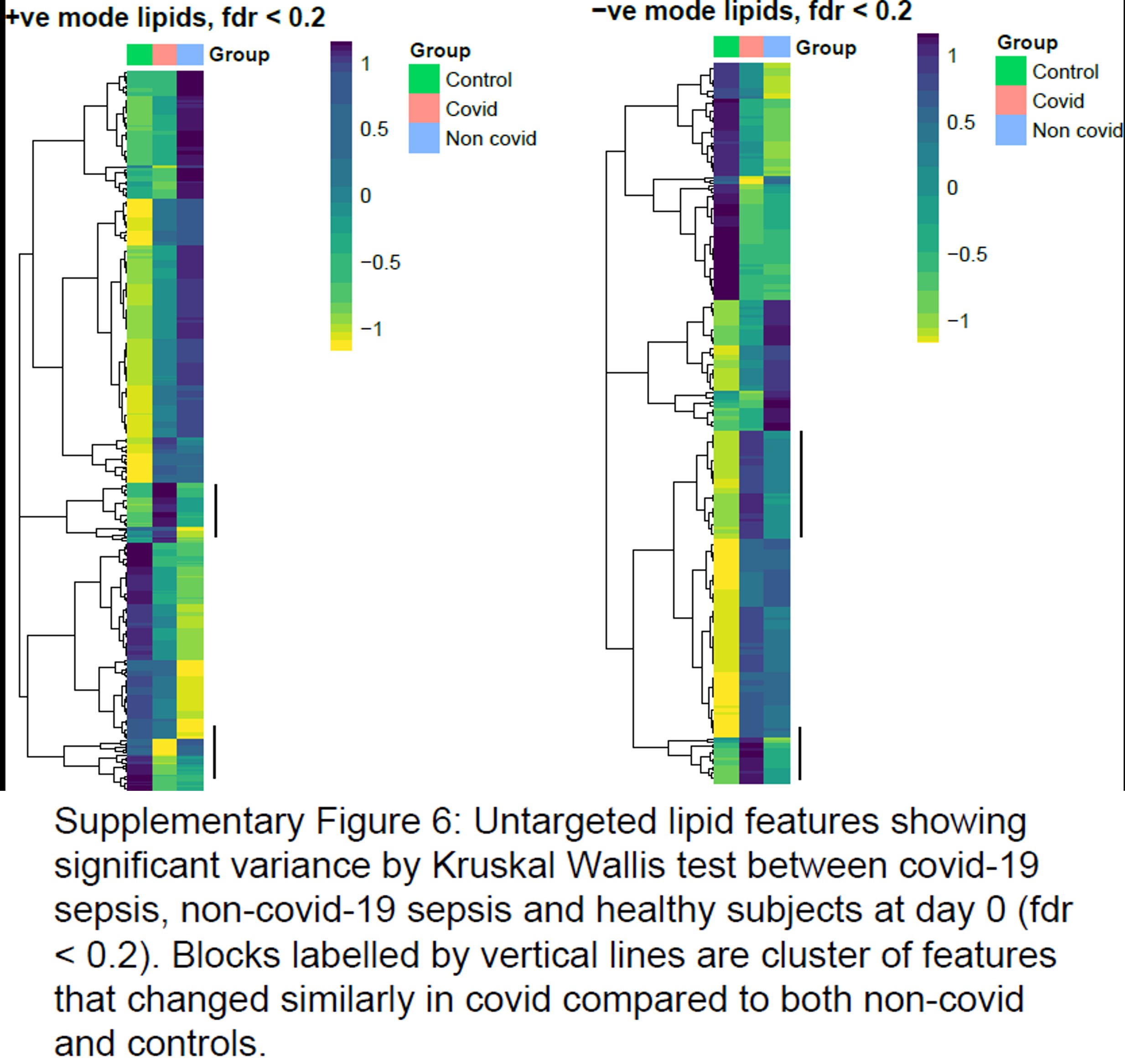

Supplement: Supplementary file 6 — Supporting Information [file CTM2-13-e1440-s003.jpg]

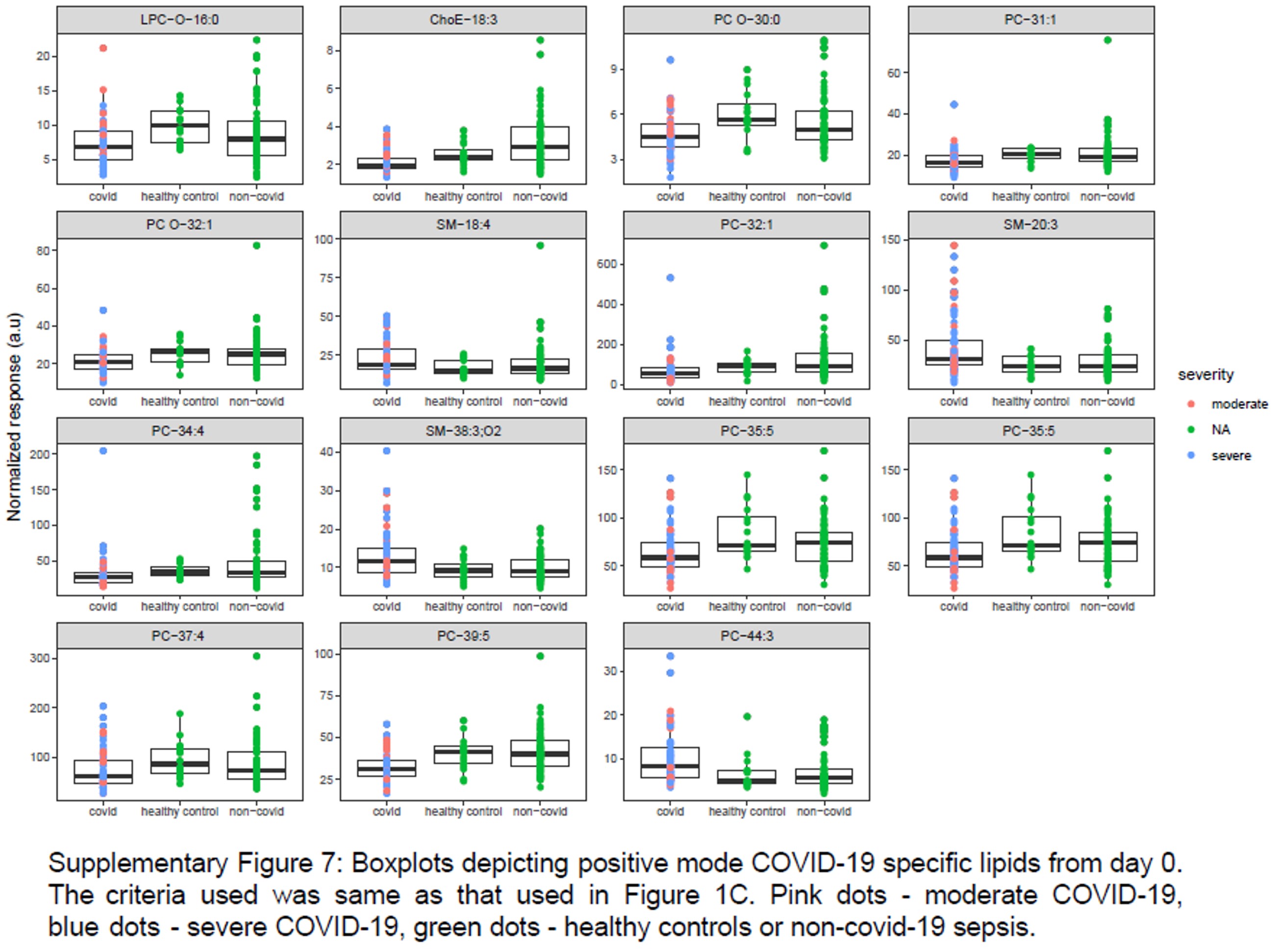

Supplement: Supplementary file 7 — Supporting Information [file CTM2-13-e1440-s008.jpg]

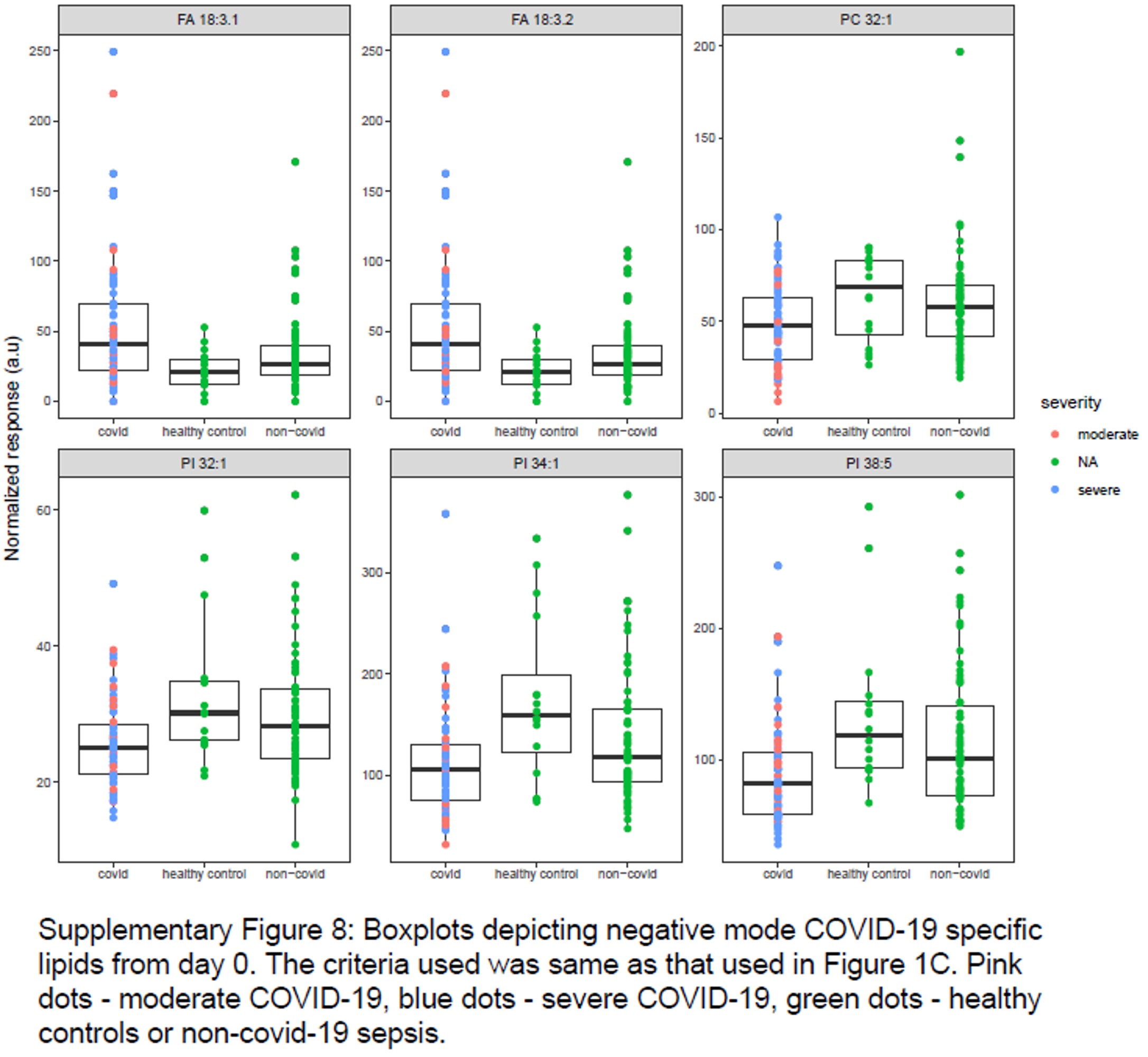

Supplement: Supplementary file 8 — Supporting Information [file CTM2-13-e1440-s004.jpg]

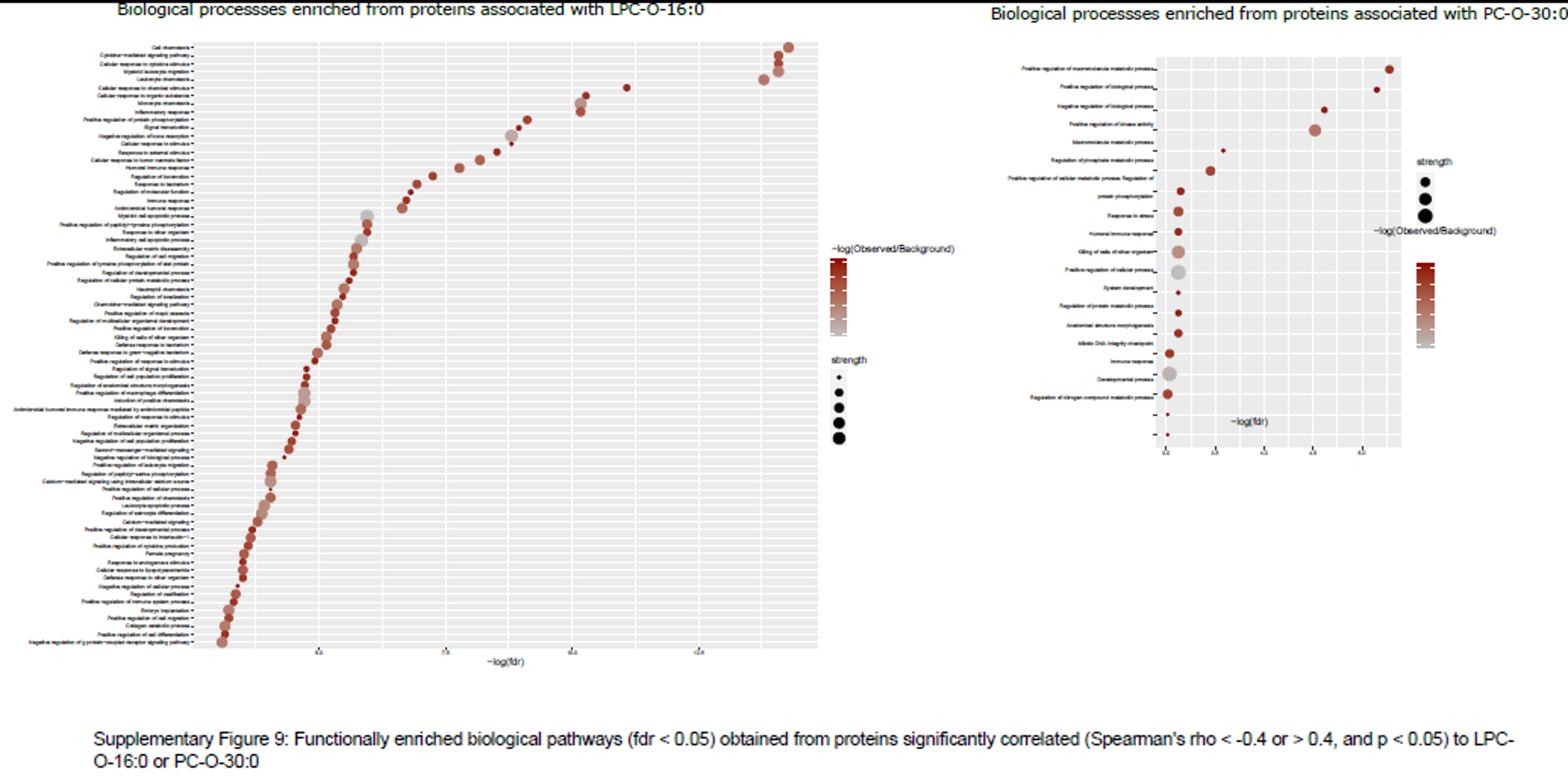

Supplement: Supplementary file 9 — Supporting Information [file CTM2-13-e1440-s001.jpg]

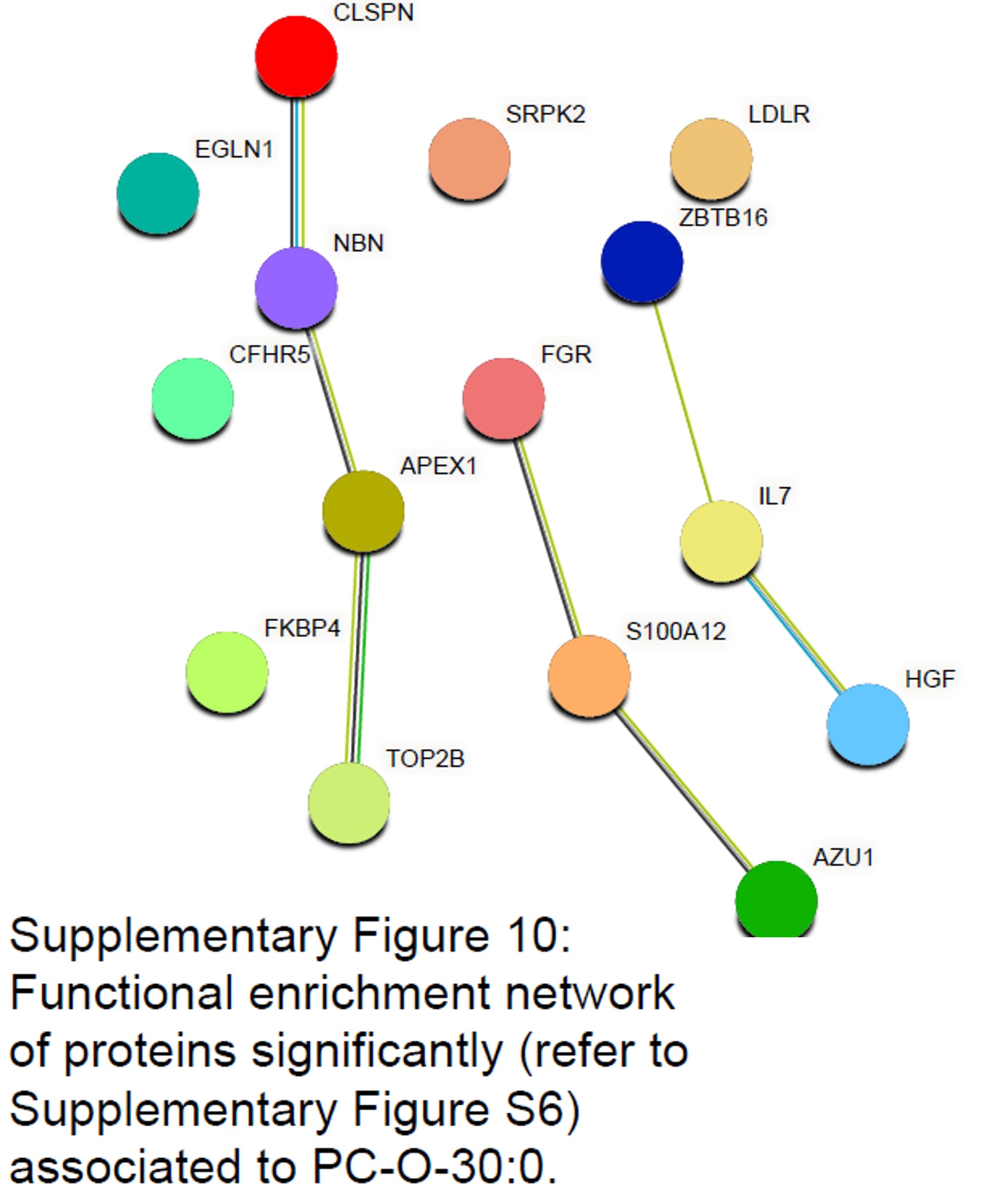

Supplement: Supplementary file 10 — Supporting Information [file CTM2-13-e1440-s009.jpg]

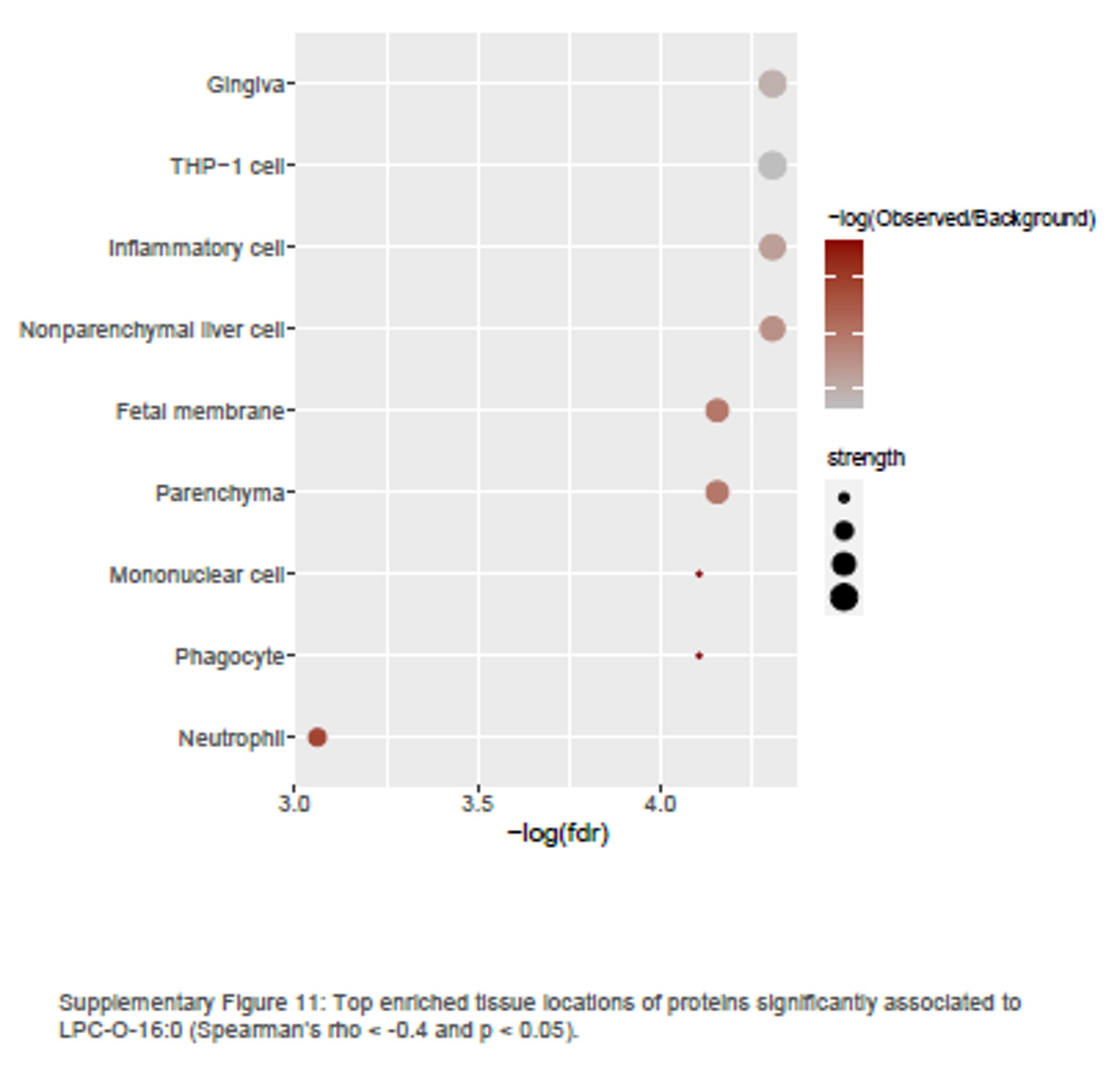

Supplement: Supplementary file 11 — Supporting Information [file CTM2-13-e1440-s014.jpg]

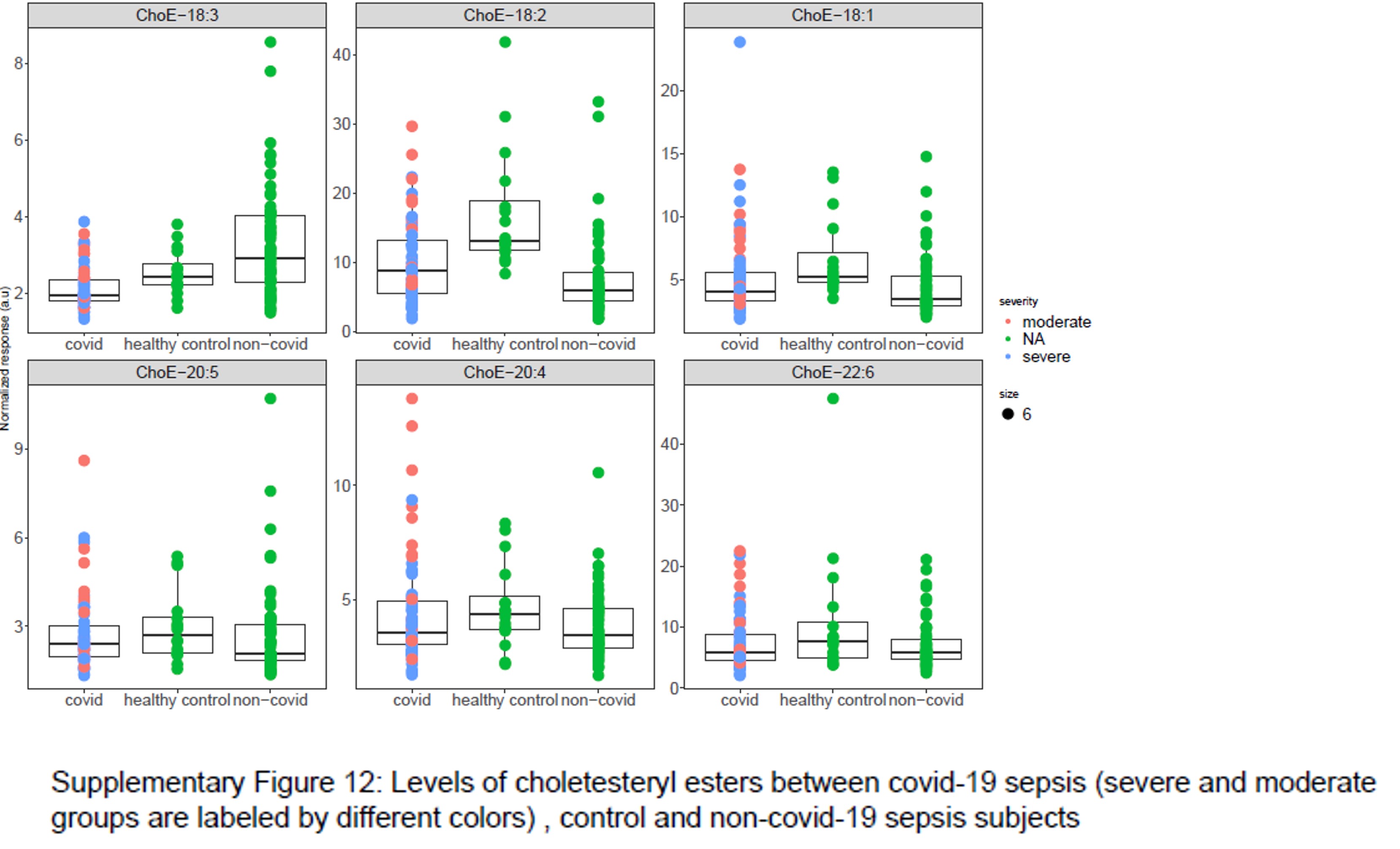

Supplement: Supplementary file 12 — Supporting Information [file CTM2-13-e1440-s005.jpg]

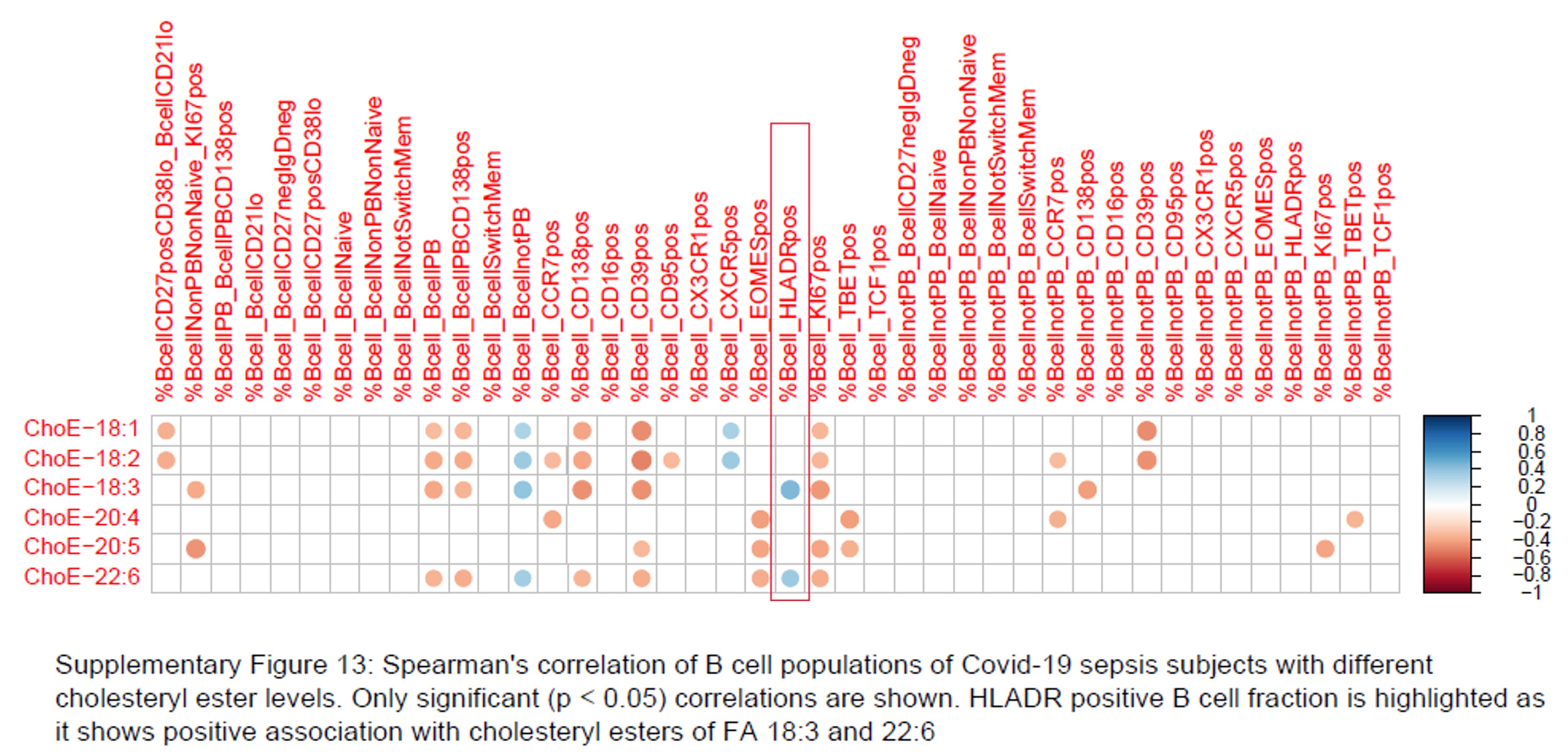

Supplement: Supplementary file 13 — Supporting Information [file CTM2-13-e1440-s011.jpg]

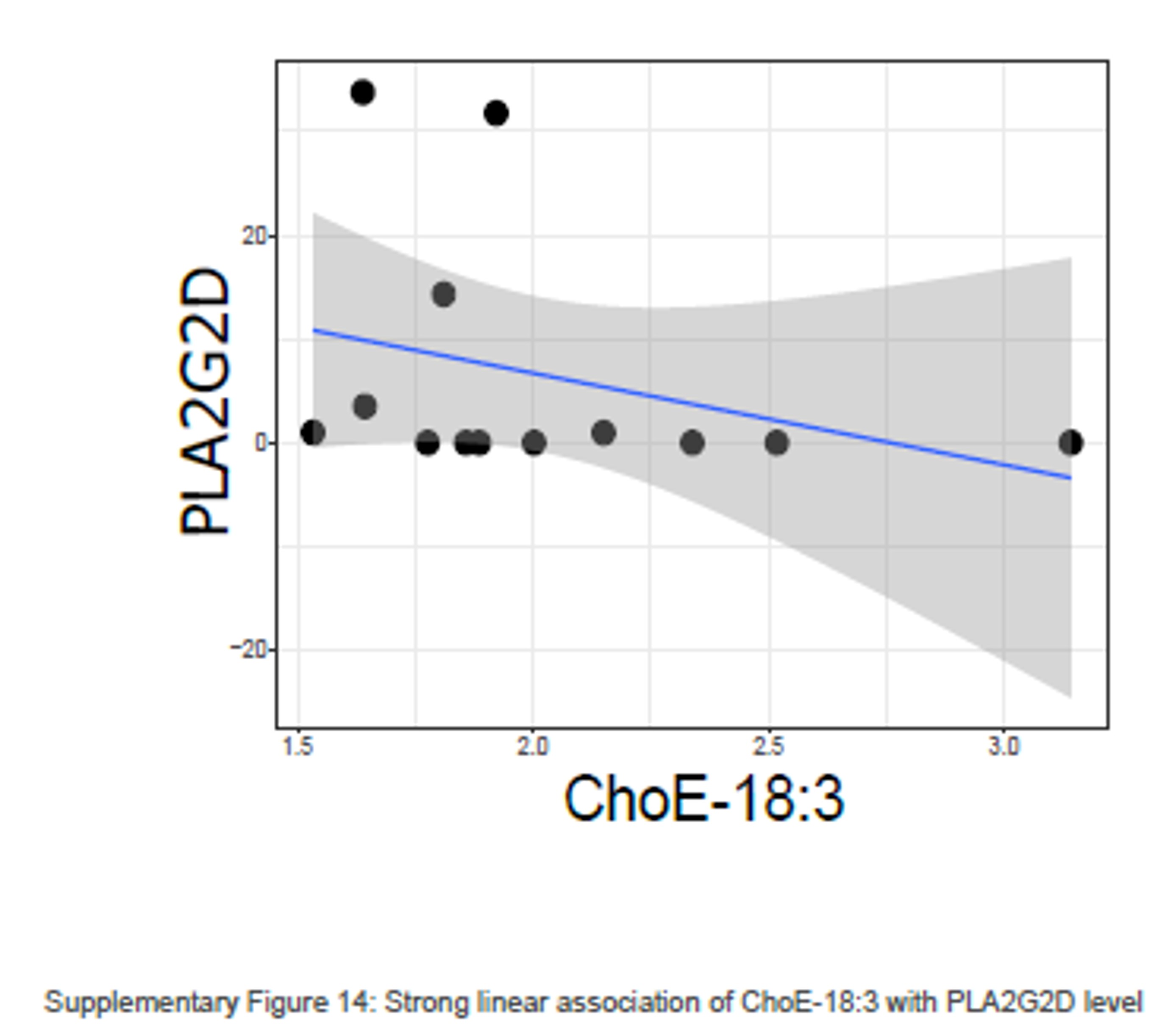

Supplement: Supplementary file 14 — Supporting Information [file CTM2-13-e1440-s007.jpg]

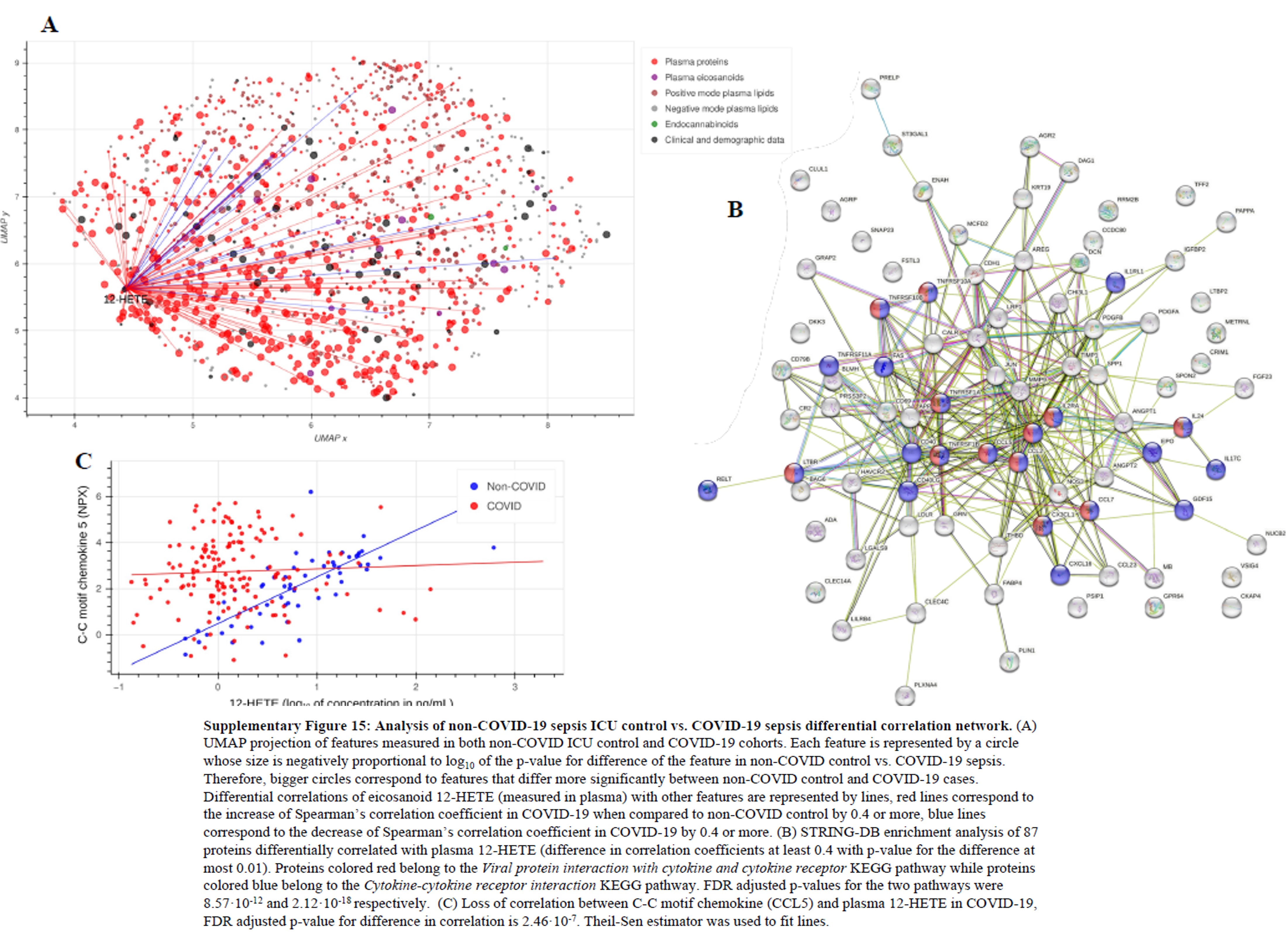

Supplement: Supplementary file 15 — Supporting Information [file CTM2-13-e1440-s006.jpg]
