# Supplementary material for: Deep phenotyping of the lipidomic response in COVID‐19 and non‐COVID‐19 sepsis
Source: Clin Transl Med. 2023 Nov 10;13(11):e1440. doi: 10.1002/ctm2.1440 (PMC10637636; doi:10.1002/ctm2.1440)
Supplement: Supplementary file 16 — Supporting Information [file CTM2-13-e1440-s002.docx]

Supplementary Table 1: Surface Antibody Staining Cocktail for PBMCs

| **Antibody** | **Company** | **Clone** | **Catalog** |
| --- | --- | --- | --- |
| Brilliant Violet 421 anti-human PD1 | Biolegend | EH12.2H7 | 329920 |
| GhostDye Violet 510 | Tonbo |  | 13-0870-T500 |
| Brilliant Violet 570 anti-human CD16 | Biolegend | 3G8 | 302036 |
| Super Bright 600 anti-human CD56 | ThermoFisher | TULY56 | 63-0566-42 |
| Brilliant Violet 650 anti-human CD45RA | Biolegend | HI100 | 304136 |
| Super Bright 702 anti-human CD8 | ThermoFisher | OKT8 | 67-0086-42 |
| Brilliant Violet 785 anti-human CD4 | Biolegend | OKT4 | 317442 |
| FITC anti-human CD88 | Biolegend | S5/1 | 344305 |
| FITC anti-human CD89 | Biolegend | A59 | 354114 |
| BB700 Mouse Anti-Human CD19 | BD | SJ25C1 | 566396 |
| PE anti-human CD39 | ThermoFisher | eBioA1 | 12-0399-41 |
| PE/Cyanine5 anti-human HLA-DR | Biolegend | L243 | 307608 |
| PE/Cy7 anti-human CD27 | BD | M-T271 | 560609 |
| Alexa Fluor647 Anti-Human CXCR5 | BD | RF8B2 | 558113 |
| AF700 anti-human CD3 | Biolegend | HIT3a | 300324 |
| APC-H7 Mouse anti-Human CD14 | BD | MφP9 | 560180 |
| Brilliant Stain Buffer | BD |  | 566349 |
| Human TruStain FcX | Biolegend |  | 422302 |

|  | Healthy control | Mild COVID-19 | Severe COVID-19 | All COVID- 19 | Non-COVID  control |
| --- | --- | --- | --- | --- | --- |
| Plasma proteins | 0 | 76 | 91 | 167 | 57 |
| Plasma eicosanoids | 16 | 67 | 86 | 153 | 249 |
| Urine eicosanoids | 26 | 105 | 71 | 176 | 11 |
| Plasma lipids | 16 | 16 | 51 | 67 | 66 |
| Flow cytometry data | 0 | 60 | 60 | 120 | 2 |
| Mass cytometry data | 0 | 5 | 7 | 12 | 0 |
| Endocannabinoids | 16 | 6 | 8 | 14 | 48 |
| **Total samples** | **26** | **142** | **127** | **269** | **263** |

**Supplementary Table 2: Sample sizes for various analyses.** Each cell represents the number of assayed samples in a specific cohort. Urine eicosanoids were measured on 11 samples from non-COVID cohort, but no other measurements were performed on these samples, making them unsuitable for integration analysis.

**Supplementary Table 3**: Features signficantly correlated to sPLA2 levels in covid subjects

| Data type | Correlated feature | Spearma ns's rho | p | n |
| --- | --- | --- | --- | --- |
| Clinical and | ards_ever | 0.2544016 | 0.039 | 66 |
| demographic data |  | 74 | 27171 |  |
|  |  |  | 6 |  |
|  | ast | 0.3354939 | 0.013 | 54 |
|  |  | 72 | 13747 |  |
|  |  |  | 1 |  |
|  | bmi | 0.2612879 | 0.034 | 66 |
|  |  | 66 | 08035 |  |
|  |  |  | 7 |  |
|  | crp | 0.6765505 | 0.000 | 23 |
|  |  | 52 | 3931 |  |
|  | daysaliveventfree_in28 | - | 0.005 | 51 |
|  |  | 0.3818415 | 69387 |  |
|  |  | 73 |  |  |
|  | ino_yn | 0.2875681 | 0.024 | 61 |
|  |  | 87 | 62641 |  |
|  |  |  | 2 |  |
|  | lymphocytes | - | 0.005 | 57 |
|  |  | 0.3620813 | 64565 |  |
|  |  | 87 | 4 |  |
|  | monocytes | - | 0.048 | 57 |
|  |  | 0.2623062 | 70571 |  |
|  |  | 22 |  |  |
|  | neutrophils_to_lymphocytes_ratio | 0.3852831 | 0.003 | 56 |
|  |  | 61 | 36475 |  |
|  | ordinal_d7 | 0.3111604 | 0.010 | 66 |
|  |  | 81 | 98888 |  |
|  |  |  | 8 |  |
|  | pct | 0.4114407 | 0.010 | 38 |
|  |  | 65 | 27750 |  |
|  |  |  | 5 |  |
|  | platelets_to_lymphocytes_ratio | 0.3798359 | 0.003 | 56 |
|  |  | 54 | 88450 |  |
|  |  |  | 6 |  |
|  | weight | 0.2835669 | 0.021 | 66 |
|  |  | 79 | 03957 |  |
|  |  |  | 7 |  |
| Flow cytometry data | percOf_Bcell_BcellPB | 0.2976249 | 0.049 | 44 |
|  |  | 21 | 75043 |  |
|  |  |  | 8 |  |

|  | percOf_Bcell_CD39pos | 0.2968392  12 | 0.050  38552  8 | 44 |
| --- | --- | --- | --- | --- |
|  | percOf_Bcell_CD95pos | 0.3030303  03 | 0.045  55457  9 | 44 |
|  | percOf_Bcell_EOMESpos | 0.3024067  09 | 0.046  02345  4 | 44 |
|  | percOf_Bcell_KI67pos | 0.3512580  18 | 0.019  38982  1 | 44 |
|  | percOf_Bcell_TBETpos | 0.3353183  69 | 0.026  07615  3 | 44 |
|  | percOf_Bcell_TCF1pos | 0.3401226  31 | 0.023  88428  8 | 44 |
|  | percOf_BcellnotPB_TBETpos | 0.3406624  38 | 0.023  64793 | 44 |
|  | percOf_CD4_CD38pos | 0.3880334  05 | 0.009  24968  1 | 44 |
|  | percOf_CD4_CD4NNHLADRposCD38pos | 0.4297998  35 | 0.003  59460  1 | 44 |
|  | percOf_CD4_CD4NNKI67pos | 0.3976039  46 | 0.007  52526  9 | 44 |
|  | percOf_CD4_HLADRposCD38pos | 0.4291201  24 | 0.003  65382  1 | 44 |
|  | percOf_CD4_KI67pos | 0.3930937  28 | 0.008  29979  6 | 44 |
|  | percOf_CD4_TBETpos | 0.3625088  09 | 0.015  59418  2 | 44 |
|  | percOf_CD4acTfh_HLADRposCD38pos | 0.4060749  15 | 0.006  23813  1 | 44 |
|  | percOf_CD4CM_HLADRposCD38pos | 0.4225103  96 | 0.004  27576 | 44 |
|  | percOf_CD4CM_KI67pos | 0.4050739  96 | 0.006  37951  9 | 44 |

|  | percOf_CD4cTfh_HLADRposCD38pos | 0.4125440  45 | 0.005  38817  8 | 44 |
| --- | --- | --- | --- | --- |
|  | percOf_CD4cTfh_KI67pos | 0.3691331  92 | 0.013  66924  4 | 44 |
|  | percOf_CD4EM1_HLADRposCD38pos | 0.4453997  68 | 0.002  44773  6 | 44 |
|  | percOf_CD4EM1_KI67pos | 0.4925473  06 | 0.000  68277  8 | 44 |
|  | percOf_CD4EM2_HLADRposCD38pos | 0.3509513  74 | 0.019  50329  3 | 44 |
|  | percOf_CD4EM2_KI67pos | 0.3141649  05 | 0.037  81545  2 | 44 |
|  | percOf_CD4EM3_HLADRposCD38pos | 0.4868217  05 | 0.000  80518  5 | 44 |
|  | percOf_CD4EM3_KI67pos | 0.3471458  77 | 0.020  95863  5 | 44 |
|  | percOf_CD4nonNaive_CD38pos | 0.4806201  55 | 0.000  95956  8 | 44 |
|  | percOf_CD4nonNaive_HLADRposCD38pos | 0.4375066  07 | 0.002  97971  2 | 44 |
|  | percOf_CD4nonNaive_KI67pos | 0.4131078  22 | 0.005  31912  2 | 44 |
|  | percOf_CD4nonNaive_TBETpos | 0.3558264  92 | 0.017  76402  3 | 44 |
|  | percOf_CD8_CD38pos | 0.4500510  94 | 0.002  17512  2 | 44 |
|  | percOf_CD8_CD95pos | 0.3315948  98 | 0.027  88797  8 | 44 |
|  | percOf_CD8_HLADRpos | 0.3406624  38 | 0.023  64793 | 44 |
|  | percOf_CD8_HLADRposCD38pos | 0.4141794  99 | 0.005  18997  6 | 44 |

|  | percOf_CD8_KI67pos | 0.4202959  83 | 0.004  50386  5 | 44 |
| --- | --- | --- | --- | --- |
|  | percOf_CD8_TBETpos | 0.3451726  57 | 0.021  74847  2 | 44 |
|  | percOf_CD8CM_HLADRposCD38pos | 0.4441155  74 | 0.002  52812  1 | 44 |
|  | percOf_CD8CM_KI67pos | 0.5208076  4 | 0.000  28964  5 | 44 |
|  | percOf_CD8EM1_HLADRposCD38pos | 0.3550387  6 | 0.018  03584  6 | 44 |
|  | percOf_CD8EM2_HLADRposCD38pos | 0.4290514  64 | 0.003  65985 | 44 |
|  | percOf_CD8EM3_HLADRposCD38pos | 0.3651867  51 | 0.014  79001  1 | 44 |
|  | percOf_CD8EMRA_HLADRposCD38pos | 0.3617463  62 | 0.015  82980  9 | 44 |
|  | percOf_CD8Ex_HLADRposCD38pos | 0.3757575  76 | 0.011  95036  2 | 44 |
|  | percOf_CD8Ex_KI67pos | 0.5167019  03 | 0.000  32960  9 | 44 |
|  | percOf_CD8nonNaive_CD38pos | 0.3897110  64 | 0.008  92490  6 | 44 |
|  | percOf_CD8nonNaive_CD95pos | 0.3873423  08 | 0.009  38639  6 | 44 |
|  | percOf_CD8nonNaive_HLADRposCD38pos | 0.3612403  1 | 0.015  98785  9 | 44 |
|  | percOf_CD8RAposCD27posR7neg_HLADRposCD 38pos | 0.4389161 | 0.002  87786 | 44 |
|  | percOf_CD8RAposCD27posR7neg_KI67pos | 0.3035663  97 | 0.045  15460  2 | 44 |
|  | percOf_CD8RAposCD27posR7posCD95pos_HLAD RposCD38pos | 0.3157968  92 | 0.036  77757  5 | 44 |

|  | percOf_CD8RAposCD27posR7posCD95pos_KI67p os | 0.3609584  21 | 0.016  07647  7 | 44 |
| --- | --- | --- | --- | --- |
|  | percOf_Live_CD4EM1 | - 0.3234672  3 | 0.032  20789  3 | 44 |
|  | percOf_Live_CD4HLADRposCD38pos | 0.4023543  63 | 0.006  77780  4 | 44 |
|  | percOf_Live_CD4NNHLADRposCD38pos | 0.4107696  69 | 0.005  61063  2 | 44 |
|  | percOf_Live_CD4nonNaive | - 0.3151626  2 | 0.037  17813  3 | 44 |
|  | percOf_Live_CD8CM | - 0.3389484  1 | 0.024  40525  2 | 44 |
|  | percOf_Live_CD8HLADRposCD38pos | 0.3040276  26 | 0.044  81276  8 | 44 |
|  | umap_component2 | 0.4331219  17 | 0.003  31710  1 | 44 |
| Mass cytometry data | %CM in CD8 | 0.9 | 0.037  38607  3 | 5 |
|  | %DCs in Intact | 0.9486832  98 | 0.013  84683  3 | 5 |
|  | %EM in CD8 | 0.9 | 0.037  38607  3 | 5 |
|  | %NaÃ¯ve in CD4 | -1 | 1.40E  -24 | 5 |
|  | %pDCs in DCs | 0.8944271  91 | 0.040  51932  6 | 5 |
|  | %Th1-like in CD4 | 0.9 | 0.037  38607  3 | 5 |
|  | Early NK | 0.9 | 0.037  38607  3 | 5 |
|  | mDC | 0.9 | 0.037  38607 | 5 |

|  |  |  | 3 |  |
| --- | --- | --- | --- | --- |
|  | pDC | 0.8944271  91 | 0.040  51932  6 | 5 |
|  | Th1-like | 0.9 | 0.037  38607  3 | 5 |
|  | Treg | 0.9 | 0.037  38607  3 | 5 |
| Negative mode plasma lipids | LPC 16:0.1 | - 0.4574002  57 | 0.005  03563  9 | 36 |
|  | LPC 16:0.2 | - 0.4574002  57 | 0.005  03563  9 | 36 |
|  | LPC 17:0 | - 0.4236808  24 | 0.010  02597  8 | 36 |
|  | LPC 18:0 | - 0.3752895  75 | 0.024  10700  4 | 36 |
|  | LPC 18:0 | - 0.3752895  75 | 0.024  10700  4 | 36 |
|  | LPC 18:1 | - 0.5907335  91 | 0.000  14880  7 | 36 |
|  | LPC 18:1 | - 0.5907335  91 | 0.000  14880  7 | 36 |
|  | LPC 18:2 | - 0.4097812  1 | 0.013  06481  6 | 36 |
|  | LPC 18:2 | - 0.4136422  14 | 0.012  15150  3 | 36 |
|  | LPC 22:5 .1 | - 0.3814671  81 | 0.021  69863  3 | 36 |
|  | LPC 22:5 .2 | - 0.3814671  81 | 0.021  69863  3 | 36 |

|  | LPE 18:0 .1 | - 0.4346035  16 | 0.008  08080  1 | 36 |
| --- | --- | --- | --- | --- |
|  | LPE 18:0 .2 | - 0.4346035  16 | 0.008  08080  1 | 36 |
|  | PC 36:3.2 | - 0.3299871  3 | 0.049  35705 | 36 |
|  | PC 37:6 | - 0.4610038  61 | 0.004  65919  7 | 36 |
|  | PC 39:6 | - 0.4996139 | 0.001  91986  6 | 36 |
|  | PC O-34:2.1 | - 0.3729729  73 | 0.025  06537  9 | 36 |
|  | PC O-34:2.2 | - 0.3729729  73 | 0.025  06537  9 | 36 |
|  | PC O-36:3.1 | - 0.4625482  63 | 0.004  50546  7 | 36 |
|  | PC O-36:3.2 | - 0.4625482  63 | 0.004  50546  7 | 36 |
|  | PC O-36:3.3 | - 0.4625482  63 | 0.004  50546  7 | 36 |
|  | PC O-38:6 | - 0.4373230  37 | 0.007  65005  3 | 36 |
|  | PC O-40:5 | - 0.3302445  3 | 0.049  16941  2 | 36 |
|  | PC O-40:6 | - 0.4550836  55 | 0.005  29125  8 | 36 |
|  | PE 40:5 | - 0.3492921  49 | 0.036  79182  8 | 36 |
|  | PE O-38:4 | - | 0.015 | 36 |

|  |  | 0.4020592  02 | 06609  4 |  |
| --- | --- | --- | --- | --- |
|  | PI 34:1 | - 0.3647361  65 | 0.028  73146  8 | 36 |
|  | PI 36:1 | - 0.4494208  49 | 0.005  96367  3 | 36 |
|  | PI 36:3 | - 0.4316602  32 | 0.008  57018  1 | 36 |
|  | PI 36:4 | - 0.4416988  42 | 0.006  99821 | 36 |
|  | PI 38:3 | - 0.4079794  08 | 0.013  51046  9 | 36 |
|  | PI 38:4 | - 0.3531531  53 | 0.034  62222  6 | 36 |
|  | PI 38:5 | - 0.3845559  85 | 0.020  57156  8 | 36 |
| Plasma eicosanoids | LTE4 | 0.3022038  77 | 0.013  65678  2 | 66 |
| Plasma proteins | CAM_O00533 Neural cell adhesion molecule L1- like protein (CHL1) | - 0.4118776  75 | 0.000  59026  1 | 66 |
|  | CAM_O15031 Plexin-B2 (PLXNB2) | 0.2634589  29 | 0.032  56762  6 | 66 |
|  | CAM_O95445 Apolipoprotein M (APOM) | - 0.3334724  98 | 0.006  21576  3 | 66 |
|  | CAM_P01033 Metalloproteinase inhibitor 1 (TIMP1) | 0.4055735  31 | 0.000  72913  8 | 66 |
|  | CAM_P05154 Plasma serine protease inhibitor (SERPINA5) | - 0.3746373  03 | 0.001  94092  5 | 66 |
|  | CAM_P06681 Complement C2 (C2) | 0.4017743  45 | 0.000  82652 | 66 |

|  | CAM_P07478 Trypsin-2 (PRSS2) | 0.3468740  22 | 0.004  32619  1 | 66 |
| --- | --- | --- | --- | --- |
|  | CAM_P10721 Mast/stem cell growth factor receptor Kit (KIT) | - 0.2425425  32 | 0.049  74118  6 | 66 |
|  | CAM_P14543 Nidogen-1 (NID1) | 0.5284834  57 | 5.090  45E-  06 | 66 |
|  | CAM_P15907 Beta-galactoside alpha-2,6- sialyltransferase 1 (ST6GAL1) | 0.5749086  73 | 4.45E  -07 | 66 |
|  | CAM_P20062 Transcobalamin-2 (TCN2) | 0.3470410  19 | 0.004  30627  6 | 66 |
|  | CAM_P22749 Granulysin (GNLY) | 0.4651497  76 | 8.316  45E-  05 | 66 |
|  | CAM_P23141 Liver carboxylesterase 1 (CES1) | 0.3498382  21 | 0.003  98453  8 | 66 |
|  | CAM_P42785 Lysosomal Pro-X carboxypeptidase (PRCP) | 0.2824966  08 | 0.021  55094  1 | 66 |
|  | CAM_Q14767 Latent-transforming growth factor beta-binding protein 2 (LTBP2) | 0.2936019  2 | 0.016  72716  6 | 66 |
|  | CAM_Q15582 Transforming growth factor-beta- induced protein ig-h3 (TGFBI) | 0.4120864  21 | 0.000  58610  4 | 66 |
|  | CAM_Q8N423 Leukocyte immunoglobulin-like receptor subfamily B member 2 (LILRB2) | 0.2753992  28 | 0.025  21603  4 | 66 |
|  | CAM_Q8NHL6 Leukocyte immunoglobulin-like receptor subfamily B member 1 (LILRB1) | 0.2982360  92 | 0.015  00669  9 | 66 |
|  | CAM_Q99650 Oncostatin-M-specific receptor subunit beta (OSMR) | 0.2712660  47 | 0.027  58359  4 | 66 |
|  | CAM_Q9BXJ1 Complement C1q tumor necrosis factor-related protein 1 (C1QTNF1) | 0.3268761  09 | 0.007  38787  3 | 66 |
|  | CAM_Q9BXR6 Complement factor H-related protein 5 (CFHR5) | 0.3570608  5 | 0.003  25017 | 66 |
|  | CAM_Q9Y5C1 Angiopoietin-related protein 3 (ANGPTL3) | 0.2946874  02 | 0.016  30970  4 | 66 |

|  | CVD2_O00182 Galectin-9 (Gal-9) | 0.2951883  94 | 0.016  12006  3 | 66 |
| --- | --- | --- | --- | --- |
|  | CVD2_O00220 Tumor necrosis factor receptor superfamily member 10A (TNFRSF10A) | 0.2533138  5 | 0.040  14884  5 | 66 |
|  | CVD2_P00797 Renin (REN) | 0.2794906  59 | 0.023  04384  5 | 66 |
|  | CVD2_P01730 T-cell surface glycoprotein CD4 (CD4) | 0.2829975  99 | 0.021  31029  5 | 66 |
|  | CVD2_P04792 Heat shock 27 kDa protein (HSP 27) | 0.3643669  76 | 0.002  63230  4 | 66 |
|  | CVD2_P05231 Interleukin-6 (IL6) | 0.3966809  31 | 0.000  97554  2 | 66 |
|  | CVD2_P07711 Cathepsin L1 (CTSL1) | 0.4170545  87 | 0.000  49469  5 | 66 |
|  | CVD2_P09341 C-X-C motif chemokine 1 (CXCL1) | 0.4558396  83 | 0.000  11993  7 | 66 |
|  | CVD2_P12931 Proto-oncogene tyrosine-protein kinase Src (SRC) | 0.3428660  89 | 0.004  82927  5 | 66 |
|  | CVD2_P18510 Interleukin-1 receptor antagonist protein (IL-1ra) | 0.4232752  32 | 0.000  39859  3 | 66 |
|  | CVD2_P19883 Follistatin (FS) | 0.2986535  85 | 0.014  85945  8 | 66 |
|  | CVD2_P21583 Stem cell factor (SCF) | - 0.3261246  22 | 0.007  53296  6 | 66 |
|  | CVD2_P26022 Pentraxin-related protein PTX3 (PTX3) | 0.3196534  81 | 0.008  88912  1 | 66 |
|  | CVD2_P35218 Carbonic anhydrase 5A, mitochondrial (CA5A) | 0.3877048  32 | 0.001  29831  7 | 66 |
|  | CVD2_Q13043 Serine/threonine-protein kinase 4 (STK4) | 0.2810771  32 | 0.022  24535  1 | 66 |
|  | CVD2_Q16698 2,4-dienoyl-CoA reductase, | 0.3301743 | 0.006  77962 | 66 |

|  | mitochondrial (DECR1) | 03 | 6 |  |
| --- | --- | --- | --- | --- |
|  | CVD2_Q99523 Sortilin (SORT1) | 0.2898862  33 | 0.018  22622  2 | 66 |
|  | CVD2_Q9BQR3 Serine protease 27 (PRSS27) | - 0.3740945  62 | 0.001  97291  6 | 66 |
|  | CVD2_Q9BUD6 Spondin-2 (SPON2) | 0.2750652  33 | 0.025  40075  1 | 66 |
|  | CVD2_Q9BWV1 Brother of CDO (Protein BOC) | - 0.2871307  8 | 0.019  41072  3 | 66 |
|  | CVD2_Q9UJM8 Hydroxyacid oxidase 1 (HAOX1) | 0.3497547  23 | 0.003  99382  5 | 66 |
|  | CVD2_Q9UKP3 Melusin (ITGB1BP2) | 0.4257801  9 | 0.000  36495  7 | 66 |
|  | CVD2_Q9Y6K9 NF-kappa-B essential modulator (NEMO) | 0.3705041  23 | 0.002  19672  2 | 66 |
|  | CVD3_O00300 Osteoprotegerin (OPG) | 0.2834353  15 | 0.022  14175 | 65 |
|  | CVD3_P00749 Urokinase-type plasminogen activator (uPA) | 0.4037150  35 | 0.000  85341  2 | 65 |
|  | CVD3_P01130 Low-density lipoprotein receptor (LDL receptor) | 0.2895541  96 | 0.019  30833  8 | 65 |
|  | CVD3_P04275 von Willebrand factor (vWF) | 0.2571241  26 | 0.038  66993  4 | 65 |
|  | CVD3_P05121 Plasminogen activator inhibitor 1 (PAI) | 0.3820367  13 | 0.001  68741  5 | 65 |
|  | CVD3_P05164 Myeloperoxidase (MPO) | 0.3772727  27 | 0.001  94829  3 | 65 |
|  | CVD3_P10451 Osteopontin (OPN) | 0.3648601  4 | 0.002  80560  2 | 65 |
|  | CVD3_P10646 Tissue factor pathway inhibitor (TFPI) | 0.2613636  36 | 0.035  46575  1 | 65 |
|  | CVD3_P13500 Monocyte chemotactic protein 1 | 0.4062062 | 0.000 | 65 |

|  | (MCP-1) | 94 | 78676  3 |  |
| --- | --- | --- | --- | --- |
|  | CVD3_P15085 Carboxypeptidase A1 (CPA1) | 0.3503496  5 | 0.004  22192 | 65 |
|  | CVD3_P20160 Azurocidin (AZU1 | 0.3085664  34 | 0.012  39050  2 | 65 |
|  | CVD3_P24158 Myeloblastin (PRTN3) | 0.4951486  01 | 2.750  43E-  05 | 65 |
|  | CVD3_P28799 Granulins (GRN) | 0.4473339  16 | 0.000  187 | 65 |
|  | CVD3_P42574 Caspase-3 (CASP-3) | 0.3360139  86 | 0.006  2092 | 65 |
|  | CVD3_Q12860 Contactin-1 (CNTN1) | -0.39375 | 0.001  17415  7 | 65 |
|  | CVD3_Q13867 Bleomycin hydrolase (BLM hydrolase) | 0.3898164  34 | 0.001  32818 | 65 |
|  | CVD3_Q15166 Paraoxonase (PON3) | - 0.2772290  21 | 0.025  36857  3 | 65 |
|  | CVD3_Q8NBP7 Proprotein convertase subtilisin/kexin type 9 (PCSK9) | 0.4212849  65 | 0.000  47445  9 | 65 |
|  | CVD3_Q92876 Kallikrein-6 (KLK6 ) | - 0.3840909  09 | 0.001  58494  6 | 65 |
|  | CVD3_Q99969 Retinoic acid receptor responder protein 2 (RARRES2) | 0.2482080  42 | 0.046  19516  4 | 65 |
|  | CVD3_Q99988 Growth/differentiation factor 15 (GDF-15) | 0.2952797  2 | 0.016  94311 | 65 |
|  | CVD3_Q9H2A7 C-X-C motif chemokine 16 (CXCL16) | 0.2985139  86 | 0.015  72013  2 | 65 |
|  | CVD3_Q9Y275 Tumor necrosis factor ligand superfamily member 13B (TNFSF13B) | 0.3793269  23 | 0.001  83167  1 | 65 |
|  | DEV_O00585 C-C motif chemokine 21 (CCL21) | 0.2818703  68 | 0.021  85499  4 | 66 |
|  | DEV_O14773 Tripeptidyl-peptidase 1 (TPP1) | 0.4223567  48 | 0.000  41161  7 | 66 |

|  | DEV_O43464 Serine protease HTRA2, mitochondrial (HTRA2) | 0.3566016  07 | 0.003  293 | 66 |
| --- | --- | --- | --- | --- |
|  | DEV_O95721 Synaptosomal-associated protein 29 (SNAP29) | 0.3970566  75 | 0.000  96377  1 | 66 |
|  | DEV_P00995 Serine protease inhibitor Kazal-type 1 (SPINK1) | 0.2991545  77 | 0.014  68441  1 | 66 |
|  | DEV_P04233 HLA class II histocompatibility antigen gamma chain (CD74) | 0.3147270  64 | 0.010  05978  5 | 66 |
|  | DEV_P07237 Protein disulfide-isomerase (P4HB) | 0.4219392  55 | 0.000  41766  5 | 66 |
|  | DEV_P14174 Macrophage migration inhibitory factor (MIF) | 0.3277110  95 | 0.007  22953  4 | 66 |
|  | DEV_P15289 Arylsulfatase A (ARSA) | 0.3869115  96 | 0.001  33102  2 | 66 |
|  | DEV_P15291 Beta-1,4-galactosyltransferase 1 (B4GALT1) | 0.4442333  79 | 0.000  18658  9 | 66 |
|  | DEV_P19256 Lymphocyte function-associated antigen 3 (CD58) | - 0.2870890  3 | 0.019  42916  3 | 66 |
|  | DEV_P23280 Carbonic anhydrase 6 (CA6) | - 0.3256653  79 | 0.007  62285  7 | 66 |
|  | DEV_P23284 Peptidyl-prolyl cis-trans isomerase B (PPIB) | 0.3841143  93 | 0.001  45235  4 | 66 |
|  | DEV_P30086 Phosphatidylethanolamine-binding protein 1 (PEBP1) | 0.2493476  67 | 0.043  48533 | 66 |
|  | DEV_P31948 Stress-induced-phosphoprotein 1 (STIP1) | 0.3230351  74 | 0.008  15598  8 | 66 |
|  | DEV_P50895 Basal cell adhesion molecule (BCAM) | 0.2938941  66 | 0.016  61388  1 | 66 |
|  | DEV_P55103 Inhibin beta C chain (INHBC) | 0.3202379  71 | 0.008  75844  3 | 66 |
|  | DEV_P63313 Thymosin beta-10 (TMSB10) | 0.2628744  39 | 0.032  96926  8 | 66 |

|  | DEV_P78552 Interleukin-13 receptor subunit alpha-1 (IL13RA1) | 0.4326688  24 | 0.000  28537  4 | 66 |
| --- | --- | --- | --- | --- |
|  | DEV_Q07108 Early activation antigen CD69 (CD69) | 0.3907107  82 | 0.001  18085  1 | 66 |
|  | DEV_Q11128,P21217 Galactoside 3(4)-L- fucosyltransferase,Alpha-(1,3)-fucosyltransferase 3/5 (FUT3/5) | 0.3323452  67 | 0.006  40365  7 | 66 |
|  | DEV_Q14112 Nidogen-2 (NID2) | 0.3011167  94 | 0.014  01578  6 | 66 |
|  | DEV_Q14118 Dystroglycan (DAG1) | 0.2880910  13 | 0.018  99069  8 | 66 |
|  | DEV_Q14696 LDLR chaperone MESD (MESDC2) | 0.3500887  17 | 0.003  95679  3 | 66 |
|  | DEV_Q4KMG0 Cell adhesion molecule- related/down-regulated by oncogenes (CDON) | - 0.3050829  77 | 0.012  74404 | 66 |
|  | DEV_Q8IWV2 Contactin-4 (CNTN4) | - 0.2543993  32 | 0.039  27358  7 | 66 |
|  | DEV_Q8TEU8 WAP, Kazal, immunoglobulin, Kunitz and NTR domain-containing protein 2 (WFIKKN2) | - 0.2792401  63 | 0.023  17211  8 | 66 |
|  | DEV_Q96QR1 Secretoglobin family 3A member 1 (SCGB3A1) | - 0.3595658  07 | 0.003  02515  5 | 66 |
|  | DEV_Q99497 Protein deglycase DJ-1 (PARK7) | 0.3104268  87 | 0.011  18895  9 | 66 |
|  | DEV_Q99538 Legumain (LGMN) | 0.2637094  25 | 0.032  39675 | 66 |
|  | DEV_Q9BY76 Angiopoietin-related protein 4 (ANGPTL4) | 0.2683018  47 | 0.029  39450  9 | 66 |
|  | DEV_Q9NQ38 Serine protease inhibitor Kazal-type 5 (SPINK5) | - 0.3009497  96 | 0.014  07165 | 66 |
|  | DEV_Q9NZV1 Cysteine-rich motor neuron 1 protein (CRIM1) | 0.3279615  91 | 0.007  18261  6 | 66 |
|  | DEV_Q9UBX1 Cathepsin F (CTSF) | - | 0.002 | 66 |

|  |  | 0.3657864  52 | 52522  3 |  |
| --- | --- | --- | --- | --- |
|  | DEV_Q9UKK9 ADP-sugar pyrophosphatase (NUDT5) | 0.3822774  24 | 0.001  53735  8 | 66 |
|  | DEV_Q9Y240 C-type lectin domain family 11 member A (CLEC11A) | 0.3723828  41 | 0.002  07693  1 | 66 |
|  | DEV_Q9Y279 V-set and immunoglobulin domain- containing protein 4 (VSIG4) | 0.2746894  9 | 0.025  60992  2 | 66 |
|  | INF_O00300 Osteoprotegerin (OPG) | 0.3429495  88 | 0.004  81828  9 | 66 |
|  | INF_O14625 C-X-C motif chemokine 11 (CXCL11) | 0.3885815  68 | 0.001  26301  3 | 66 |
|  | INF_O14788 TNF-related activation-induced cytokine (TRANCE) | - 0.3271683  54 | 0.007  33211  3 | 66 |
|  | INF_O15169 Axin-1 (AXIN1) | 0.3421563  51 | 0.004  92355  8 | 66 |
|  | INF_O43557 Tumor necrosis factor ligand superfamily member 14 (TNFSF14 ) | 0.3685836  55 | 0.002  32552  8 | 66 |
|  | INF_O95630 STAM-binding protein (STAMPB) | 0.2612044  67 | 0.034  13969  1 | 66 |
|  | INF_P00749 Urokinase-type plasminogen activator (uPA) | 0.3968479  28 | 0.000  97029  5 | 66 |
|  | INF_P00813 Adenosine Deaminase (ADA) | 0.2950631  46 | 0.016  16729  5 | 66 |
|  | INF_P01137 Latency-associated peptide transforming growth factor beta-1 (LAP TGF-beta- 1) | 0.3004070  56 | 0.014  25453  7 | 66 |
|  | INF_P01579 Interferon gamma (IFN-gamma) | 0.2746894  9 | 0.025  60992  2 | 66 |
|  | INF_P02778 C-X-C motif chemokine 10 (CXCL10 ) | 0.4523744  91 | 0.000  13708  1 | 66 |
|  | INF_P05231 Interleukin-6 (IL6) | 0.4243607  14 | 0.000  38368 | 66 |

|  |  |  | 5 |  |
| --- | --- | --- | --- | --- |
|  | INF_P09341 C-X-C motif chemokine 1 (CXCL1) | 0.4477403  19 | 0.000  16353  7 | 66 |
|  | INF_P09603 Macrophage colony-stimulating factor 1 (CSF-1) | 0.4290783  84 | 0.000  32462  2 | 66 |
|  | INF_P10145 Interleukin-8 (IL-8) | 0.4652750  23 | 8.274  99E-  05 | 66 |
|  | INF_P13232 Interleukin-7 (IL-7) | 0.4025675  82 | 0.000  80526  5 | 66 |
|  | INF_P13500 Monocyte chemotactic protein 1 (MCP-1) | 0.4422711  62 | 0.000  20075  3 | 66 |
|  | INF_P13725 Oncostatin-M (OSM) | 0.2915979  54 | 0.017  52195  7 | 66 |
|  | INF_P14210 Hepatocyte growth factor (HGF) | 0.3231604  22 | 0.008  12988  1 | 66 |
|  | INF_P15018 Leukemia inhibitory factor (LIF) | 0.2769857  01 | 0.024  35405  6 | 66 |
|  | INF_P21583 Stem cell factor (SCF) | - 0.2723932  78 | 0.026  92004  6 | 66 |
|  | INF_P22301 Interleukin-10 (IL10) | 0.4745851  16 | 5.675  98E-  05 | 66 |
|  | INF_P42830 C-X-C motif chemokine 5 (CXCL5 ) | 0.2477611  94 | 0.044  88244 | 66 |
|  | INF_P50225 Sulfotransferase 1A1 (ST1A1) | 0.2848763  18 | 0.020  42811  5 | 66 |
|  | INF_P55773 C-C motif chemokine 23 (CCL23) | 0.3524684  27 | 0.003  70163  5 | 66 |
|  | INF_P78556 C-C motif chemokine 20 (CCL20) | 0.3458720  38 | 0.004  44740  7 | 66 |
|  | INF_P80075 Monocyte chemotactic protein 2 (MCP-2) | 0.4418536  69 | 0.000  20389  1 | 66 |
|  | INF_P80098 Monocyte chemotactic protein 3 | 0.5497338 | 1.746 | 66 |

|  | (MCP-3) | 48 | 48E-  06 |  |
| --- | --- | --- | --- | --- |
|  | INF_P80162 C-X-C motif chemokine 6 (CXCL6) | 0.3173155  2 | 0.009  42898  5 | 66 |
|  | INF_P80511 Protein S100-A12 (EN-RAGE ) | 0.4312075  98 | 0.000  30079  1 | 66 |
|  | INF_Q13478 Interleukin-18 receptor 1 (IL-18R1) | 0.5343283  58 | 3.820  41E-  06 | 66 |
|  | INF_Q14790 Caspase-8 (CASP-8 ) | 0.3773927  56 | 0.001  78556  8 | 66 |
|  | INF_Q8IXJ6 SIR2-like protein 2 (SIRT2) | 0.2765264  59 | 0.024  60097  3 | 66 |
|  | INF_Q8NFT8 Delta and Notch-like epidermal growth factor-related receptor (DNER) | - 0.3893748  04 | 0.001  23182 | 66 |
|  | INF_Q99731 C-C motif chemokine 19 (CCL19) | 0.2776119  4 | 0.024  02073  3 | 66 |
|  | INF_Q9H5V8 CUB domain-containing protein 1 (CDCP1) | 0.2852938  11 | 0.020  23635  5 | 66 |
|  | INF_Q9NZQ7 Programmed cell death 1 ligand 1 (PD-L1) | 0.2843753  26 | 0.020  66026  7 | 66 |
|  | IRE_O00273 DNA fragmentation factor subunit alpha (DFFA) | 0.4216052  6 | 0.000  42256  1 | 66 |
|  | IRE_O14867 Transcription regulator protein BACH1 (BACH1) | 0.4749191  11 | 5.598  6E-05 | 66 |
|  | IRE_O43597 Protein sprouty homolog 2 (SPRY2) | 0.3412378  67 | 0.005  04798  1 | 66 |
|  | IRE_O75475 PC4 and SFRS1-interacting protein (PSIP1) | 0.4028180  77 | 0.000  79865  7 | 66 |
|  | IRE_O94992 Protein HEXIM1 (HEXIM1) | 0.4580106  46 | 0.000  11022  5 | 66 |
|  | IRE_O95786 Probable ATP-dependent RNA helicase DDX58 (DDX58) | 0.3910447  76 | 0.001  16840  9 | 66 |

|  | IRE_P05231 Interleukin-6 (IL6) | 0.4459868  49 | 0.000  17471  4 | 66 |
| --- | --- | --- | --- | --- |
|  | IRE_P08727 Keratin, type I cytoskeletal 19 (KRT19) | 0.3388164  07 | 0.005  38940  3 | 66 |
|  | IRE_P09038 Fibroblast growth factor 2 (FGF2) | 0.2881327  63 | 0.018  97261  3 | 66 |
|  | IRE_P14317 Hematopoietic lineage cell-specific protein (HCLS1) | 0.3967644  3 | 0.000  97291  5 | 66 |
|  | IRE_P16278 Beta-galactosidase (GLB1) | 0.4458198  52 | 0.000  17581  5 | 66 |
|  | IRE_P16455 Methylated-DNA--protein-cysteine methyltransferase (MGMT) | 0.3366036  95 | 0.005  71902  4 | 66 |
|  | IRE_P18627 Lymphocyte activation gene 3 protein (LAG3) | 0.3104686  36 | 0.011  17748  9 | 66 |
|  | IRE_P19474 E3 ubiquitin-protein ligase TRIM21 (TRIM21) | 0.4295793  76 | 0.000  31886  4 | 66 |
|  | IRE_P22301 Interleukin-10 (IL10) | 0.4966704  94 | 2.219  09E-  05 | 66 |
|  | IRE_P30044 Peroxiredoxin-5, mitochondrial (PRDX5) | 0.4067007  62 | 0.000  70231  6 | 66 |
|  | IRE_P30048 Thioredoxin-dependent peroxide reductase, mitochondrial (PRDX3) | 0.4634380  54 | 8.902  63E-  05 | 66 |
|  | IRE_P50135 Histamine N-methyltransferase (HNMT) | 0.2539818  39 | 0.039  60833  4 | 66 |
|  | IRE_P51617 Interleukin-1 receptor-associated kinase 1 (IRAK1) | 0.4253209  48 | 0.000  37092  3 | 66 |
|  | IRE_P78362 SRSF protein kinase 2 (SRPK2) | 0.4064920  15 | 0.000  70721  4 | 66 |
|  | IRE_P78410 Butyrophilin subfamily 3 member A2 (BTN3A2) | 0.4128796  58 | 0.000  57054  9 | 66 |
|  | IRE_Q00978 Interferon regulatory factor 9 (IRF9) | 0.4545037  05 | 0.000  12629 | 66 |

|  |  |  | 8 |  |
| --- | --- | --- | --- | --- |
|  | IRE_Q04637 Eukaryotic translation initiation factor 4 gamma 1 (EIF4G1) | 0.4130884  04 | 0.000  56651  9 | 66 |
|  | IRE_Q05084 Islet cell autoantigen 1 (ICA1) | 0.4230247  36 | 0.000  40210  7 | 66 |
|  | IRE_Q05516 Zinc finger and BTB domain- containing protein 16 (ZBTB16) | 0.3987266  46 | 0.000  91299  3 | 66 |
|  | IRE_Q06830 Peroxiredoxin-1 (PRDX1) | 0.3397766  41 | 0.005  25164  9 | 66 |
|  | IRE_Q07065 Cytoskeleton-associated protein 4 (CKAP4) | 0.3820686  78 | 0.001  54729  5 | 66 |
|  | IRE_Q12968 Nuclear factor of activated T-cells, cytoplasmic 3 (NFATC3) | 0.3018682  81 | 0.013  76675  5 | 66 |
|  | IRE_Q13490 Baculoviral IAP repeat-containing protein 2 (BIRC2) | 0.4211042  69 | 0.000  43000  4 | 66 |
|  | IRE_Q14203 Dynactin subunit 1 (DCTN1) | 0.3425738  44 | 0.004  86790  2 | 66 |
|  | IRE_Q8IU57 Interferon lambda receptor 1 (IFNLR1) | 0.3952614  55 | 0.001  02119  3 | 66 |
|  | IRE_Q8NHJ6 Leukocyte immunoglobulin-like receptor subfamily B member 4 (LILRB4) | 0.4183070  66 | 0.000  4738 | 66 |
|  | IRE_Q96DB9 FXYD domain-containing ion transport regulator 5 (FXYD5) | 0.3097171  49 | 0.011  38552  3 | 66 |
|  | IRE_Q96SB3 Neurabin-2 (PPP1R9B) | 0.3392756  5 | 0.005  32312  8 | 66 |
|  | IRE_Q9C035 Tripartite motif-containing protein 5 (TRIM5) | 0.5104060  12 | 1.196  59E-  05 | 66 |
|  | IRE_Q9GZT9 Egl nine homolog 1 (EGLN1) | 0.2525206  14 | 0.040  79862 | 66 |
|  | IRE_Q9HCM2 Plexin-A4 (PLXNA4) | 0.2741467  49 | 0.025  91462  5 | 66 |
|  | IRE_Q9NWZ3 Interleukin-1 receptor-associated kinase 4 (IRAK4) | 0.2681348  5 | 0.029  49943 | 66 |

|  |  |  | 5 |  |
| --- | --- | --- | --- | --- |
|  | IRE_Q9UKX5 Integrin alpha-11 (ITGA11) | - 0.2510593  88 | 0.042  01834  8 | 66 |
|  | IRE_Q9UQQ2 SH2B adapter protein 3 (SH2B3) | 0.2881745  12 | 0.018  95454  3 | 66 |
|  | IRE_Q9UQV4 Lysosome-associated membrane glycoprotein 3 (LAMP3) | 0.3338899  91 | 0.006  14741  3 | 66 |
|  | IRE_Q9Y2J8 Protein-arginine deiminase type-2 (PADI2) | 0.2754409  77 | 0.025  19302  4 | 66 |
|  | IRE_Q9Y3P8 Signaling threshold-regulating transmembrane adapter 1 (SIT1) | - 0.3640329  82 | 0.002  65808  1 | 66 |
|  | MET_O00161 Synaptosomal-associated protein 23 (SNAP23) | 0.2695125  77 | 0.028  64312  7 | 66 |
|  | MET_O15123 Angiopoietin-2 (ANGPT2) | 0.3991858  89 | 0.000  89946 | 66 |
|  | MET_O75356 Ectonucleoside triphosphate diphosphohydrolase 5 (ENTPD5) | 0.3833629  06 | 0.001  48660  4 | 66 |
|  | MET_O75791 GRB2-related adapter protein 2 (GRAP2) | 0.3695021  4 | 0.002  26310  6 | 66 |
|  | MET_O95544 NAD kinase (NADK) | 0.4907003  44 | 2.878  8E-05 | 66 |
|  | MET_O95841 Angiopoietin-related protein 1 (ANGPTL1) | 0.3447448  07 | 0.004  58735  8 | 66 |
|  | MET_P09104 Gamma-enolase (ENO2) | 0.2941029  12 | 0.016  53336  7 | 66 |
|  | MET_P09417 Dihydropteridine reductase (QDPR) | 0.4571756  6 | 0.000  11387  1 | 66 |
|  | MET_P09668 Pro-cathepsin H (CTSH) | 0.2745224  92 | 0.025  70335  3 | 66 |
|  | MET_P12724 Eosinophil cationic protein (RNASE3) | 0.4168040  91 | 0.000  49897  4 | 66 |
|  | MET_P16083 Ribosyldihydronicotinamide | 0.2589917 | 0.035  74371 | 66 |

|  | dehydrogenase [quinone] (NQO2) | 55 | 9 |  |
| --- | --- | --- | --- | --- |
|  | MET_P19022 Cadherin-2 (CDH2) | 0.3585220  75 | 0.003  11716  8 | 66 |
|  | MET_P19971 Thymidine phosphorylase (TYMP) | 0.4679195  8 | 8.482  05E-  05 | 65 |
|  | MET_P21964 Catechol O-methyltransferase (COMT) | 0.4450683  64 | 0.000  18084  5 | 66 |
|  | MET_P23526 Adenosylhomocysteinase (AHCY) | 0.2921824  44 | 0.017  28687  2 | 66 |
|  | MET_P27695 DNA-(apurinic or apyrimidinic site) lyase (APEX1) | 0.4377622  38 | 0.000  23711  2 | 66 |
|  | MET_P31431 Syndecan-4 (SDC4) | 0.2753992  28 | 0.025  21603  4 | 66 |
|  | MET_P35754 Glutaredoxin-1 (GLRX) | 0.3526771  74 | 0.003  67996  5 | 66 |
|  | MET_P41236 Protein phosphatase inhibitor 2 (PPP1R2) | 0.4550464  46 | 0.000  12367  7 | 66 |
|  | MET_P43234 Cathepsin O (CTSO) | 0.4273249  14 | 0.000  34552  8 | 66 |
|  | MET_P46109 Crk-like protein (CRKL) | 0.4275754  1 | 0.000  34246  8 | 66 |
|  | MET_P50452 Serpin B8 (SERPINB8) | 0.4089552  24 | 0.000  65133  9 | 66 |
|  | MET_P51693 Amyloid-like protein 1 (APLP1) | - 0.3261246  22 | 0.007  53296  6 | 66 |
|  | MET_P52888 Thimet oligopeptidase (THOP1) | 0.3225759  32 | 0.008  25234  2 | 66 |
|  | MET_P98082 Disabled homolog 2 (DAB2) | 0.4286191  42 | 0.000  32998  2 | 66 |
|  | MET_Q02790 Peptidyl-prolyl cis-trans isomerase FKBP4 (FKBP4) | 0.4178060  75 | 0.000  48206 | 66 |
|  | MET_Q13275 Semaphorin-3F (SEMA3F) | 0.3038304 | 0.013 | 66 |

|  |  | 98 | 13441  5 |  |
| --- | --- | --- | --- | --- |
|  | MET_Q15155 Nodal modulator 1 (NOMO1) | 0.3391171  33 | 0.005  72028  1 | 65 |
|  | MET_Q16773 Kynurenine--oxoglutarate transaminase 1 (KYAT1) | 0.3310510  39 | 0.006  62552  2 | 66 |
|  | MET_Q76M96 Coiled-coil domain-containing protein 80 (CCDC80) | 0.2481369  38 | 0.044  54825 | 66 |
|  | MET_Q8N1Q1 Carbonic anhydrase 13 (CA13) | 0.3539296  52 | 0.003  55229  6 | 66 |
|  | MET_Q8NI22 Multiple coagulation factor deficiency protein 2 (MCFD2) | 0.4347562  88 | 0.000  26460  1 | 66 |
|  | MET_Q8WVQ1 Soluble calcium-activated nucleotidase 1 (CANT1) | 0.2961903  77 | 0.015  74645  3 | 66 |
|  | MET_Q96JA1 Leucine-rich repeats and immunoglobulin-like domains protein 1 (LRIG1) | 0.4900741  05 | 2.957  65E-  05 | 66 |
|  | MET_Q9GZM7 Tubulointerstitial nephritis antigen-like (TINAGL1) | 0.3366454  44 | 0.005  71264  4 | 66 |
|  | MET_Q9NY25 C-type lectin domain family 5 member A (CLEC5A) | 0.2719340  36 | 0.027  18873  8 | 66 |
|  | ODA_O15357 Phosphatidylinositol 3,4,5- trisphosphate 5-phosphatase 2 (INPPL1) | 0.3953032  04 | 0.001  01982  3 | 66 |
|  | ODA_O60240 Perilipin-1 (PLIN1) | 0.2877152  7 | 0.019  15412  2 | 66 |
|  | ODA_O60934 Nibrin (NBN) | 0.3734683  23 | 0.002  01041  6 | 66 |
|  | ODA_O75354 Ectonucleoside triphosphate diphosphohydrolase 6 (ENTPD6) | 0.2885920  05 | 0.018  77465  1 | 66 |
|  | ODA_O95994 Anterior gradient protein 2 homolog (AGR2) | 0.2505166  48 | 0.042  47899  6 | 66 |
|  | ODA_P01258 Calcitonin (CALCA) | 0.3161882  89 | 0.009  69932  4 | 66 |

|  | ODA_P07947 Tyrosine-protein kinase Yes (YES1) | 0.3499634  69 | 0.003  97064  4 | 66 |
| --- | --- | --- | --- | --- |
|  | ODA_P09769 Tyrosine-protein kinase Fgr (FGR) | 0.3443273  14 | 0.004  64017  1 | 66 |
|  | ODA_P09960 Leukotriene A-4 hydrolase (LTA4H) | 0.3607765  37 | 0.002  92145  7 | 66 |
|  | ODA_P40121 Macrophage-capping protein (CAPG) | 0.2877570  19 | 0.019  13590  5 | 66 |
|  | ODA_P42658 Dipeptidyl aminopeptidase-like protein 6 (DPP6) | - 0.3841143  93 | 0.001  45235  4 | 66 |
|  | ODA_P49023 Paxillin (PXN) | 0.3769335  14 | 0.001  81065  9 | 66 |
|  | ODA_P53539 Protein fosB (FOSB) | 0.3918380  13 | 0.001  13933  4 | 66 |
|  | ODA_P55957 BH3-interacting domain death agonist (BID) | 0.2600772  36 | 0.034  94916  6 | 66 |
|  | ODA_P61244 Protein max (MAX) | 0.4012733  54 | 0.000  84020  5 | 66 |
|  | ODA_P80303 Nucleobindin-2 (NUCB2) | 0.3181087  57 | 0.009  24270  1 | 66 |
|  | ODA_P98073 Enteropeptidase (TMPRSS15) | - 0.2856725  36 | 0.020  06373 | 66 |
|  | ODA_Q02246 Contactin-2 (CNTN2) | - 0.3698778  83 | 0.002  23800  5 | 66 |
|  | ODA_Q02880 DNA topoisomerase 2-beta (TOP2B) | 0.4207285  25 | 0.000  43566  4 | 66 |
|  | ODA_Q07954 Prolow-density lipoprotein receptor- related protein 1 (LRP1) | 0.3545976  41 | 0.003  48582  7 | 66 |
|  | ODA_Q11201 CMP-N-acetylneuraminate-beta- galactosamide-alpha-2,3-sialyltransferase 1  (ST3GAL1) | 0.4737918  8 | 5.863  75E-  05 | 66 |

|  | ODA_Q12778 Forkhead box protein O1 (FOXO1) | 0.2569460  39 | 0.037  28204  2 | 66 |
| --- | --- | --- | --- | --- |
|  | ODA_Q12913 Receptor-type tyrosine-protein phosphatase eta (PTPRJ) | 0.4156351  11 | 0.000  51938  9 | 66 |
|  | ODA_Q15165 Serum paraoxonase/arylesterase 2 (PON2) | 0.3160630  41 | 0.009  72977  4 | 66 |
|  | ODA_Q15797 Mothers against decapentaplegic homolog 1 (SMAD1) | 0.3826531  68 | 0.001  51961  5 | 66 |
|  | ODA_Q7L5Y9 Macrophage erythroblast attacher (MAEA) | 0.2500574  05 | 0.042  87202  4 | 66 |
|  | ODA_Q7LG56 Ribonucleoside-diphosphate reductase subunit M2 B (RRM2B) | 0.4074522  49 | 0.000  68493  5 | 66 |
|  | ODA_Q86SJ6 Desmoglein-4 (DSG4) | - 0.3776850  02 | 0.001  76976  3 | 66 |
|  | ODA_Q86SR1 Polypeptide N- acetylgalactosaminyltransferase 10 (GALNT10) | 0.2474272 | 0.045  18122  8 | 66 |
|  | ODA_Q8N8S7 Protein enabled homolog (ENAH) | 0.3145600  67 | 0.010  10171  3 | 66 |
|  | ODA_Q8NDB2 B-cell scaffold protein with ankyrin repeats (BANK1) | 0.2724350  28 | 0.026  89573 | 66 |
|  | ODA_Q96RT1 Erbin (ERBIN) | 0.3195282  33 | 0.008  91734  4 | 66 |
|  | ODA_Q9GZY6 Linker for activation of T-cells family member 2 (LAT2) | 0.2490136  73 | 0.043  77643  9 | 66 |
|  | ODA_Q9NQ88 Fructose-2,6-bisphosphatase TIGAR (TIGAR) | 0.3310092  89 | 0.006  63279 | 66 |
|  | ODA_Q9NRA1 Platelet-derived growth factor C (PDGFC) | 0.2531051  04 | 0.040  31900  2 | 66 |
|  | ODA_Q9UKL0 REST corepressor 1 (RCOR1) | 0.3464982  78 | 0.004  3713 | 66 |
|  | ODA_Q9ULX7 Carbonic anhydrase 14 (CA14) | - 0.3134745  85 | 0.010  37796  5 | 66 |

|  | ODA_Q9UNK0 Syntaxin-8 (STX8) | 0.2518943  74 | 0.041  31772  5 | 66 |
| --- | --- | --- | --- | --- |
|  | ODA_Q9Y478 5'-AMP-activated protein kinase subunit beta-1 (PRKAB1) | 0.2494311  66 | 0.043  41280  3 | 66 |
|  | ODA_Q9Y4K4 Mitogen-activated protein kinase kinase kinase kinase 5 (MAP4K5) | 0.2964826  22 | 0.015  63889  4 | 66 |
|  | ODA_Q9Y5A7 NEDD8 ultimate buster 1 (NUB1) | 0.3107608  81 | 0.011  09748  2 | 66 |
| Positive mode plasma lipids | ChoE-18:1 | - 0.3371943  37 | 0.044  31549  6 | 36 |
|  | ChoE-18:2 | - 0.3534105  53 | 0.034  48137  9 | 36 |
|  | LPC-14:0 | - 0.4903474  9 | 0.002  39747  2 | 36 |
|  | LPC-15:0 | - 0.5801801  8 | 0.000  20808  6 | 36 |
|  | LPC-16:0 | - 0.4195624  2 | 0.010  85624  6 | 36 |
|  | LPC-17:0 | - 0.4944658  94 | 0.002  17371  5 | 36 |
|  | LPC-18:0 | - 0.3683397  68 | 0.027  07671  9 | 36 |
|  | LPC-18:1 | - 0.3500643  5 | 0.036  34928  6 | 36 |
|  | LPC-18:3 | - 0.3474903  47 | 0.037  84148  1 | 36 |
|  | LPC-19:3 | - 0.3294723  29 | 0.049  73404  9 | 36 |
|  | LPC-20:5 | - 0.3747747 | 0.024  31731 | 36 |

|  |  | 75 | 1 |  |
| --- | --- | --- | --- | --- |
|  | LPC-22:6 | - 0.3567567  57 | 0.032  69252  2 | 36 |
|  | LPC-O-16:0 | - 0.4337194  34 | 0.008  22522  2 | 36 |
|  | LPC-O-22:0 | - 0.3850707  85 | 0.020  38854  9 | 36 |
|  | SM-17:0 | - 0.3371943  37 | 0.044  31549  6 | 36 |
|  | SM-20:1 | - 0.3613899  61 | 0.030  34160  8 | 36 |
|  | SM-26:5 | - 0.3338481  34 | 0.046  60198  9 | 36 |
|  | SM-31:1;O2 17:1;O2/14:0 | - 0.4234234  23 | 0.010  07623  9 | 36 |
|  | SM-32:0;O2 | - 0.3891891  89 | 0.018  97264  5 | 36 |
|  | SM-33:2;O2 | - 0.3616473  62 | 0.030  21518 | 36 |
|  | SM-35:1;O2 18:1;O2/17:0 | - 0.3371943  37 | 0.044  31549  6 | 36 |
|  | SM-35:1;O2 21:1;O2/14:0 | - 0.3371943  37 | 0.044  31549  6 | 36 |
|  | SM-38:2;O2 18:2;O2/20:0 | - 0.3613899  61 | 0.030  34160  8 | 36 |
| Urine eicosanoids | 11-dehydro TxB2 | 0.4487394  96 | 0.006  85444  1 | 35 |
|  | iPF2a-III | 0.3353925  35 | 0.045  53517  8 | 36 |

Supplementary table 4 Positive mode targeted lipid list

| Index | exact.mass | ID | Duplicate p | Class |
| --- | --- | --- | --- | --- |
| 1 | 468.3079 | LPC-14:0 |  | LPC |
| 2 | 480.3087 | LPE-18:1 |  | LPE |
| 3 | 480.3436 | LPC-15:1 |  | LPC |
| 4 | 482.3245 | LPC-15:0 |  | LPC |
| 5 | 482.3606 | LPC-O-16:0 |  | LPC-O |
| 6 | 494.3243 | LPC-16:1 |  | LPC |
| 7 | 496.3399 | LPC-16:0 |  | LPC |
| 8 | 502.2926 | LPE-20:4 |  | LPE |
| 9 | 508.3758 | LPC-17:1 |  | LPC |
| 10 | 510.3558 | LPC-17:0 |  | LPC |
| 11 | 518.3246 | LPC-18:3 |  | LPC |
| 12 | 520.3394 | LPC-18:2 | 2 | LPC |
| 13 | 520.3397 | LPC-18:2 | 1 | LPC |
| 14 | 522.3538 | LPC-18:1 |  | LPC |
| 15 | 524.3726 | LPC-18:0 |  | LPC |
| 16 | 528.3084 | LPE-22:5 |  | LPE |
| 17 | 532.3395 | LPC-19:3 |  | LPC |
| 18 | 534.3914 | LPC-O-20:2 |  | LPC-O |
| 19 | 536.3705 | LPC-19:1 |  | LPC |
| 20 | 536.4075 | LPC-O-20:1 |  | LPC-O |
| 21 | 538.3873 | LPC-19:0 | 2 | LPC |
| 22 | 538.3885 | LPC-19:0 | 1 | LPC |
| 23 | 542.3225 | LPC-20:5 |  | LPC |
| 24 | 544.3421 | LPC-20:4 |  | LPC |
| 25 | 546.3555 | LPC-20:3 |  | LPC |
| 26 | 550.3869 | LPC-20:1 |  | LPC |
| 27 | 552.4025 | LPC-20:0 |  | LPC |
| 28 | 558.5094 | DG-30:0 |  | DG |
| 29 | 566.4539 | LPC-O-22:0 |  | LPC-O |
| 30 | 568.3428 | LPC-22:6 |  | LPC |
| 31 | 578.418 | LPC-22:1 |  | LPC |
| 32 | 580.4336 | LPC-22:0 |  | LPC |
| 33 | 582.5093 | DG-32:2 |  | DG |
| 34 | 584.5251 | DG-32:1 |  | DG |
| 35 | 586.5408 | DG-32:0 |  | DG |
| 36 | 594.5819 | Cer-38:1 |  | Cer |
| 37 | 606.4501 | LPC-24:1 |  | LPC |
| 38 | 606.5095 | DG-34:4 |  | DG |
| 39 | 608.5248 | DG-34:3 |  | DG |
| 40 | 610.5403 | DG-34:2 |  | DG |
| 41 | 612.5562 | DG-34:1 |  | DG |
| 42 | 614.5723 | DG-34:0 |  | DG |
| 43 | 620.5978 | Cer-40:2 |  | Cer |
| 44 | 622.6133 | Cer-40:1 | 3 | Cer |
| 45 | 632.5257 | DG-36:5 |  | DG |
| 46 | 634.5405 | DG-36:4 | 2 | DG |

| 47 | 634.5409 | DG-36:4 | 1 | DG |
| --- | --- | --- | --- | --- |
| 48 | 634.6133 | Cer-41:2 |  | Cer |
| 49 | 636.556 | DG-36:3 |  | DG |
| 50 | 636.6295 | Cer-41:1 |  | Cer |
| 51 | 638.5712 | DG-36:2 |  | DG |
| 52 | 638.645 | Cer-41:0 |  | Cer |
| 53 | 640.5872 | DG-36:1 |  | DG |
| 54 | 642.6035 | DG-36:0 |  | DG |
| 55 | 646.6137 | Cer-40:1 | 1 | Cer |
| 56 | 647.5124 | SM-30:1;O2 18:1;O2/1 | | SM |
| 57 | 648.6292 | Cer-40:1 | 2 | Cer |
| 58 | 648.6295 | Cer-42:2 |  | Cer |
| 59 | 650.6445 | Cer-42:1 |  | Cer |
| 60 | 656.5248 | DG-38:7 |  | DG |
| 61 | 658.5405 | DG-38:6 |  | DG |
| 62 | 660.5562 | DG-38:5 |  | DG |
| 63 | 661.5275 | SM-31:1;O2 17:1;O2/1 | | SM |
| 64 | 663.4521 | SM-14:6 |  | SM |
| 65 | 664.4935 | PC-28:7 |  | PC |
| 66 | 664.6057 | ChoE-18:3 |  | ChoE |
| 67 | 666.6193 | ChoE-18:2 |  | ChoE |
| 68 | 668.6337 | ChoE-18:1 |  | ChoE |
| 69 | 673.528 | SM-32:2;O2 18:2;O2/1 | | SM |
| 70 | 675.5436 | SM-32:1;O2 16:1;O2/1 | | SM |
| 71 | 675.544 | SM-14:0 |  | SM |
| 72 | 677.5595 | SM-32:0;O2 |  | SM |
| 73 | 687.5435 | SM-33:2;O2 |  | SM |
| 74 | 688.6016 | ChoE-20:5 |  | ChoE |
| 75 | 689.5591 | SM-33:1;O2 17:1;O2/1 | | SM |
| 76 | 689.5617 | SM-15:0 |  | SM |
| 77 | 690.6189 | ChoE-20:4 |  | ChoE |
| 78 | 692.559 | PC O-30:0 |  | PC-O |
| 79 | 701.5564 | SM-16:1 |  | SM |
| 80 | 701.5591 | SM-34:2;O2 18:2;O2/1 | | SM |
| 81 | 702.5056 | PC-30:2 |  | PC |
| 82 | 703.5747 | SM-34:1;O2 |  | SM |
| 83 | 703.5762 | SM-34:1;O2 18:1;O2/1 | | SM |
| 84 | 703.5767 | SM-16:0 |  | SM |
| 85 | 704.5582 | PE O-34:1 |  | PE-O |
| 86 | 705.5909 | SM-34:0;O2 18:0;O2/1 | | SM |
| 87 | 711.5465 | SM-17:3 |  | SM |
| 88 | 714.5433 | PE O-35:3 |  | PE-O |
| 89 | 714.6185 | ChoE-22:6 |  | ChoE |
| 90 | 715.5753 | SM-35:2;O2 21:2;O2/1 | | SM |
| 91 | 716.5593 | PC O-32:2 |  | PC-O |
| 92 | 717.5898 | SM-17:0 |  | SM |
| 93 | 717.5908 | SM-35:1;O2 18:1;O2/1 | | SM |

| 94 | 717.5911 | SM-35:1;O2 21:1;O2/1 | | SM |
| --- | --- | --- | --- | --- |
| 95 | 718.5394 | PC-31:1 |  | PC |
| 96 | 718.5748 | PC O-32:1 |  | PC-O |
| 97 | 720.5544 | PC-31:0 |  | PC |
| 98 | 720.5545 | PC-31:0 |  | PC |
| 99 | 723.5447 | SM-18:4 |  | SM |
| 100 | 725.56 | SM-18:3 |  | SM |
| 101 | 727.5752 | SM-36:3;O2 18:1;O2/1 | | SM |
| 102 | 728.5228 | PC-32:3 |  | PC |
| 103 | 729.5915 | SM-36:2;O2 18:2;O2/1 | | SM |
| 104 | 729.5917 | SM-18:1 |  | SM |
| 105 | 730.5387 | PC-32:2 |  | PC |
| 106 | 731.6069 | SM-36:1;O2 16:1;O2/2 | | SM |
| 107 | 731.6089 | SM-18:0 |  | SM |
| 108 | 732.5576 | PC-32:1 |  | PC |
| 109 | 733.6226 | SM-36:0;O2 |  | SM |
| 110 | 734.5718 | PC-32:0 |  | PC |
| 111 | 734.6068 | PC O-33:0 |  | PC-O |
| 112 | 739.5718 | SM-37:4;O2 |  | SM |
| 113 | 740.6752 | TG-42:0 |  | TG |
| 114 | 742.5386 | PC-33:3 |  | PC |
| 115 | 742.5741 | PC O-34:3 | 1 | PC-O |
| 116 | 742.575 | PC O-34:3 | 2 | PC-O |
| 117 | 745.6218 | SM-37:1;O2 18:1;O2/1 | | SM |
| 118 | 746.605 | PC O-34:1 |  | PC-O |
| 119 | 748.5283 | PE O-38:7 |  | PE-O |
| 120 | 748.5856 | PC-33:0 |  | PC |
| 121 | 748.6213 | PC O-34:0 |  | PC-O |
| 122 | 751.5771 | SM-20:4 |  | SM |
| 123 | 752.522 | PC-34:5 |  | PC |
| 124 | 753.5944 | SM-20:3 |  | SM |
| 125 | 754.5378 | PC-34:4 |  | PC |
| 126 | 754.5747 | PE O-38:4 |  | PE-O |
| 127 | 754.5748 | PC O-35:4 | 1 | PC-O |
| 128 | 754.5754 | PC O-35:4 | 2 | PC-O |
| 129 | 754.6968 | TG-43:0 |  | TG |
| 130 | 755.6072 | SM-38:3;O2 |  | SM |
| 131 | 756.5535 | PC-34:3 |  | PC |
| 132 | 756.5908 | PC O-35:3 | 1 | PC-O |
| 133 | 756.5909 | PC O-35:3 | 2 | PC-O |
| 134 | 757.6225 | SM-20:1 |  | SM |
| 135 | 757.6226 | SM-38:2;O2 18:2;O2/2 | | SM |
| 136 | 758.5718 | PC-34:2 |  | PC |
| 137 | 758.6063 | PC O-35:2 |  | PC-O |
| 138 | 759.6376 | SM-38:1;O2 18:1;O2/2 | | SM |
| 139 | 759.6407 | SM-20:0 |  | SM |
| 140 | 760.5883 | PC-34:1 |  | PC |

| 141 | 761.652 | SM-38:0;O2 |  | SM |
| --- | --- | --- | --- | --- |
| 142 | 762.6032 | PC-34:0 |  | PC |
| 143 | 764.5231 | PC-35:6 |  | PC |
| 144 | 764.6807 | TG-44:2 |  | TG |
| 145 | 766.538 | PC-35:5 |  | PC |
| 146 | 766.5385 | PC-35:5 |  | PC |
| 147 | 766.575 | PC O-36:5 | 2 | PC-O |
| 148 | 766.5752 | PC O-36:5 | 1 | PC-O |
| 149 | 766.6937 | TG-44:1 |  | TG |
| 150 | 768.5568 | PC-35:4 |  | PC |
| 151 | 768.5771 | PC O-36:4 |  | PC-O |
| 152 | 770.5698 | PC-35:3 |  | PC |
| 153 | 772.5274 | PE O-40:9 |  | PE-O |
| 154 | 772.5858 | PC-35:2 | 2 | PC |
| 155 | 772.5861 | PC-35:2 | 1 | PC |
| 156 | 772.6209 | PC O-36:2 | 1 | PC-O |
| 157 | 772.6215 | PC O-36:2 | 2 | PC-O |
| 158 | 773.6527 | SM-39:1;O2 16:1;O2/2 | | SM |
| 159 | 773.6563 | SM-21:0 |  | SM |
| 160 | 774.6008 | PC-35:1 |  | PC |
| 161 | 778.5362 | PC-36:6 | 1 | PC |
| 162 | 778.5386 | PC-36:6 | 2 | PC |
| 163 | 780.5514 | PC-36:5 | 1 | PC |
| 164 | 780.5523 | PC-36:5 | 2 | PC |
| 165 | 780.5905 | PC O-37:5 |  | PC-O |
| 166 | 782.5695 | PC-36:4 | 4 | PC |
| 167 | 782.5715 | PC-36:4 | 1 | PC |
| 168 | 782.5718 | PC-36:4 | 2 | PC |
| 169 | 782.5728 | PC-36:4 | 3 | PC |
| 170 | 782.593 | PC O-37:4 |  | PC-O |
| 171 | 782.7242 | TG-45:0 |  | TG |
| 172 | 783.6379 | SM-40:3;O2 |  | SM |
| 173 | 784.5768 | PC-36:3 | 2 | PC |
| 174 | 784.5831 | PC-36:3 | 1 | PC |
| 175 | 785.653 | SM-40:2;O2 18:2;O2/2 | | SM |
| 176 | 785.6532 | SM-40:2;O2 16:1;O2/2 | | SM |
| 177 | 785.6536 | SM-22:1 | 1 | SM |
| 178 | 785.657 | SM-22:1 | 2 | SM |
| 179 | 786.6012 | PC-36:2 |  | PC |
| 180 | 787.6685 | SM-40:1;O2 18:1;O2/2 | | SM |
| 181 | 787.6719 | SM-22:0 |  | SM |
| 182 | 788.6165 | PC-36:1 |  | PC |
| 183 | 789.684 | SM-40:0;O2 |  | SM |
| 184 | 790.5381 | PC-37:7 |  | PC |
| 185 | 790.5723 | PC O-38:7 | 1 | PC-O |
| 186 | 790.5743 | PC O-38:7 | 3 | PC-O |
| 187 | 790.5756 | PC O-38:7 | 2 | PC-O |

| 188 | 790.6325 | PC-36:0 |  | PC |
| --- | --- | --- | --- | --- |
| 189 | 790.6967 | TG-46:3 |  | TG |
| 190 | 792.5908 | PC O-38:6 |  | PC-O |
| 191 | 792.7114 | TG-46:2 |  | TG |
| 192 | 794.569 | PC-37:5 |  | PC |
| 193 | 794.6059 | PC O-38:5 | 1 | PC-O |
| 194 | 794.6062 | PC O-38:5 | 2 | PC-O |
| 195 | 794.722 | TG-46:1 |  | TG |
| 196 | 796.5855 | PC-37:4 |  | PC |
| 197 | 796.7368 | TG-46:0 |  | TG |
| 198 | 798.6011 | PC-37:3 |  | PC |
| 199 | 798.6368 | PC O-38:3 |  | PC-O |
| 200 | 799.6657 | SM-23:1 | 1 | SM |
| 201 | 799.6688 | SM-41:2;O2 17:1;O2/2 | | SM |
| 202 | 799.6701 | SM-23:1 | 2 | SM |
| 203 | 801.6845 | SM-41:1;O2 18:1;O2/2 | | SM |
| 204 | 801.6848 | SM-41:1;O2 |  | SM |
| 205 | 801.6851 | SM-23:0 | 2 | SM |
| 206 | 801.6882 | SM-23:0 | 1 | SM |
| 207 | 802.5403 | PC-38:8 |  | PC |
| 208 | 802.6321 | PC-37:1 |  | PC |
| 209 | 804.5527 | PC-37:0 |  | PC |
| 210 | 804.5535 | PC-38:7 |  | PC |
| 211 | 804.5905 | PE O-42:7 |  | PE-O |
| 212 | 804.7063 | TG-47:3 |  | TG |
| 213 | 806.5669 | PC-38:6 | 1 | PC |
| 214 | 806.5672 | PC-38:6 | 2 | PC |
| 215 | 806.7217 | TG-47:2 |  | TG |
| 216 | 807.6346 | SM-24:4 | 1 | SM |
| 217 | 807.6374 | SM-42:5;O2 |  | SM |
| 218 | 807.6377 | SM-24:4 | 2 | SM |
| 219 | 808.5831 | PC-38:5 |  | PC |
| 220 | 808.739 | TG-47:1 |  | TG |
| 221 | 809.6502 | SM-42:4;O2 18:1;O2/2 | | SM |
| 222 | 809.6506 | SM-24:3 |  | SM |
| 223 | 809.6533 | SM-42:4;O2 18:2;O2/2 | | SM |
| 224 | 810.5992 | PC-38:4 | 4 | PC |
| 225 | 810.6016 | PC-38:4 | 2 | PC |
| 226 | 810.6037 | PC-38:4 | 3 | PC |
| 227 | 810.6044 | PC-38:4 | 1 | PC |
| 228 | 811.6653 | SM-24:2 |  | SM |
| 229 | 811.6691 | SM-42:3;O2 18:2;O2/2 | | SM |
| 230 | 812.6152 | PC-38:3 |  | PC |
| 231 | 813.6838 | SM-24:1 | 1 | SM |
| 232 | 813.685 | SM-42:2;O2 18:1;O2/2 | | SM |
| 233 | 813.6871 | SM-24:1 | 2 | SM |
| 234 | 814.5734 | PC O-40:9 |  | PC-O |

| 235 | 814.6338 | PC-38:2 |  | PC |
| --- | --- | --- | --- | --- |
| 236 | 815.6998 | SM-42:1;O2 |  | SM |
| 237 | 815.7006 | SM-42:1;O2 18:1;O2/2 | | SM |
| 238 | 815.7028 | SM-24:0 |  | SM |
| 239 | 816.5884 | PC O-40:8 | 1 | PC-O |
| 240 | 816.5898 | PC O-40:8 | 2 | PC-O |
| 241 | 816.5906 | PC O-40:8 | 3 | PC-O |
| 242 | 816.6472 | PC-38:1 |  | PC |
| 243 | 818.5699 | PC-39:7 |  | PC |
| 244 | 818.6038 | PC O-40:7 | 3 | PC-O |
| 245 | 818.606 | PC O-40:7 | 2 | PC-O |
| 246 | 818.6063 | PC O-40:7 | 4 | PC-O |
| 247 | 818.6064 | PC O-40:7 | 1 | PC-O |
| 248 | 818.6649 | PC-38:0 |  | PC |
| 249 | 818.7239 | TG-48:3 | 2 | TG |
| 250 | 818.7251 | TG-48:3 | 1 | TG |
| 251 | 820.5859 | PC-39:6 |  | PC |
| 252 | 820.6201 | PC O-40:6 | 2 | PC-O |
| 253 | 820.6208 | PC O-40:6 | 1 | PC-O |
| 254 | 820.6224 | PC O-40:6 |  | PC-O |
| 255 | 820.7375 | TG-48:2 |  | TG |
| 256 | 822.6011 | PC-39:5 |  | PC |
| 257 | 822.6373 | PC O-40:5 | 2 | PC-O |
| 258 | 822.638 | PC O-40:5 | 1 | PC-O |
| 259 | 822.7565 | TG-48:1 |  | TG |
| 260 | 823.6712 | SM-25:3 |  | SM |
| 261 | 824.6165 | PC-39:4 |  | PC |
| 262 | 824.6531 | PC O-40:4 | 2 | PC-O |
| 263 | 824.6534 | PC O-40:4 | 1 | PC-O |
| 264 | 824.77 | TG-48:0 | 2 | TG |
| 265 | 824.7726 | TG-48:0 | 1 | TG |
| 266 | 826.5356 | PC-40:10 |  | PC |
| 267 | 826.6689 | PC O-40:3 | 1 | PC-O |
| 268 | 826.6738 | PC O-40:3 | 2 | PC-O |
| 269 | 827.7 | SM-43:2;O2 18:2;O2/2 | | SM |
| 270 | 827.7007 | SM-43:2;O | 1 | SM |
| 271 | 827.7011 | SM-43:2;O | 2 | SM |
| 272 | 827.7049 | SM-25:1 |  | SM |
| 273 | 828.5542 | PC-40:9 |  | PC |
| 274 | 829.7124 | SM-25:0 |  | SM |
| 275 | 829.7154 | SM-43:1;O2 |  | SM |
| 276 | 829.7158 | SM-43:1;O2 18:1;O2/2 | | SM |
| 277 | 832.5808 | PC-39:0 |  | PC |
| 278 | 832.583 | PC-40:7 |  | PC |
| 279 | 832.5853 | PC-40:7 |  | PC |
| 280 | 832.744 | TG-49:3 |  | TG |
| 281 | 833.6518 | SM-26:5 |  | SM |

| 282 | 834.5983 | PC-40:6 |  | PC |
| --- | --- | --- | --- | --- |
| 283 | 834.6 | PC-40:6 |  | PC |
| 284 | 834.7592 | TG-49:2 |  | TG |
| 285 | 835.6672 | SM-26:4 | 2 | SM |
| 286 | 835.6685 | SM-26:4 | 1 | SM |
| 287 | 836.6119 | PC-40:5 |  | PC |
| 288 | 837.6869 | SM-26:3 |  | SM |
| 289 | 839.7038 | SM-26:2 |  | SM |
| 290 | 840.6478 | PC-40:3 | 2 | PC |
| 291 | 840.6486 | PC-40:3 | 1 | PC |
| 292 | 841.7189 | SM-26:1 |  | SM |
| 293 | 842.6638 | PC-40:2 |  | PC |
| 294 | 842.7266 | TG-50:5 |  | TG |
| 295 | 844.6195 | PC O-42:8 | 1 | PC-O |
| 296 | 844.6223 | PC O-42:8 | 2 | PC-O |
| 297 | 844.7397 | TG-50:4 |  | TG |
| 298 | 846.6364 | PC O-42:7 | 1 | PC-O |
| 299 | 846.6367 | PC O-42:7 | 3 | PC-O |
| 300 | 846.6382 | PC O-42:7 | 2 | PC-O |
| 301 | 846.7554 | TG-50:3 | 2 | TG |
| 302 | 846.7567 | TG-50:3 | 1 | TG |
| 303 | 848.6528 | PC O-42:6 | 1 | PC-O |
| 304 | 848.653 | PC O-42:6 | 2 | PC-O |
| 305 | 848.7715 | TG-50:2 |  | TG |
| 306 | 850.6685 | PC O-42:5 | 1 | PC-O |
| 307 | 850.6687 | PC O-42:5 | 2 | PC-O |
| 308 | 850.7871 | TG-50:1 |  | TG |
| 309 | 852.6838 | PC O-42:4 | 1 | PC-O |
| 310 | 852.6847 | PC O-42:4 | 2 | PC-O |
| 311 | 854.7004 | PC O-42:3 |  | PC-O |
| 312 | 855.7324 | SM-27:1 |  | SM |
| 313 | 856.585 | PC-42:9 |  | PC |
| 314 | 856.5852 | PC-42:9 |  | PC |
| 315 | 856.7147 | PC O-42:2 |  | PC-O |
| 316 | 856.7151 | PC O-42:2 |  | PC-O |
| 317 | 858.6001 | PC-42:8 |  | PC |
| 318 | 858.7546 | TG-51:4 |  | TG |
| 319 | 860.6141 | PC-42:7 |  | PC |
| 320 | 860.6174 | PC-42:7 |  | PC |
| 321 | 860.7722 | TG-51:3 |  | TG |
| 322 | 862.6323 | PC-42:6 | 1 | PC |
| 323 | 862.6334 | PC-42:6 | 2 | PC |
| 324 | 862.7913 | TG-51:2 |  | TG |
| 325 | 864.6472 | PC-42:5 |  | PC |
| 326 | 864.8056 | TG-51:1 | 1 | TG |
| 327 | 864.8061 | TG-51:1 | 2 | TG |
| 328 | 866.6629 | PC-42:4 |  | PC |

| 329 | 866.6647 | PC-42:4 |  | PC |
| --- | --- | --- | --- | --- |
| 330 | 866.8201 | TG-51:0 |  | TG |
| 331 | 868.74 | TG-52:6 |  | TG |
| 332 | 870.6345 | PC O-44:9 |  | PC-O |
| 333 | 870.6355 | PC O-44:9 |  | PC-O |
| 334 | 870.6952 | PC-42:2 |  | PC |
| 335 | 870.7558 | TG-52:5 |  | TG |
| 336 | 872.6536 | PC O-44:8 |  | PC-O |
| 337 | 872.772 | TG-52:4 |  | TG |
| 338 | 874.6682 | PC O-44:7 |  | PC-O |
| 339 | 874.6685 | PC O-44:7 |  | PC-O |
| 340 | 874.7885 | TG-52:3 |  | TG |
| 341 | 876.8063 | TG-52:2 |  | TG |
| 342 | 878.6985 | PC O-44:5 |  | PC-O |
| 343 | 878.8224 | TG-52:1 |  | TG |
| 344 | 880.715 | PC O-44:4 | 1 | PC-O |
| 345 | 880.7159 | PC O-44:4 | 2 | PC-O |
| 346 | 880.8375 | TG-52:0 |  | TG |
| 347 | 885.7817 | SM-29:0 |  | SM |
| 348 | 890.8201 | TG-53:2 |  | TG |
| 349 | 892.8337 | TG-53:1 |  | TG |
| 350 | 893.751 | SM-30:3 |  | SM |
| 351 | 894.6971 | PC-44:4 |  | PC |
| 352 | 894.7515 | TG-54:7 |  | TG |
| 353 | 896.6501 | PC O-46:10 |  | PC-O |
| 354 | 896.7118 | PC-44:3 |  | PC |
| 355 | 896.772 | TG-54:6 | 2 | TG |
| 356 | 896.774 | TG-54:6 | 1 | TG |
| 357 | 898.7225 | PC-44:2 |  | PC |
| 358 | 898.7861 | TG-54:5 |  | TG |
| 359 | 900.6853 | PC O-46:8 |  | PC-O |
| 360 | 900.7373 | PC-44:1 |  | PC |
| 361 | 900.8053 | TG-54:4 |  | TG |
| 362 | 902.8207 | TG-54:3 |  | TG |
| 363 | 904.7165 | PC O-46:6 |  | PC-O |
| 364 | 904.8358 | TG-54:2 |  | TG |
| 365 | 906.8511 | TG-54:1 |  | TG |
| 366 | 908.8682 | TG-54:0 |  | TG |
| 367 | 916.8366 | TG-55:3 |  | TG |
| 368 | 918.8494 | TG-55:2 |  | TG |
| 369 | 920.7708 | TG-56:8 |  | TG |
| 370 | 920.869 | TG-55:1 |  | TG |
| 371 | 922.7908 | TG-56:7 |  | TG |
| 372 | 924.7462 | PC-46:3 |  | PC |
| 373 | 924.8026 | TG-56:6 |  | TG |
| 374 | 926.8182 | TG-56:5 |  | TG |
| 375 | 928.769 | PC-46:1 |  | PC |

| 376 | 928.8317 | TG-56:4 | TG |
| --- | --- | --- | --- |
| 377 | 930.8494 | TG-56:3 | TG |
| 378 | 932.86 | TG-56:2 | TG |
| 379 | 934.8801 | TG-56:1 | TG |
| 380 | 944.7735 | TG-58:10 | TG |
| 381 | 946.7879 | TG-58:9 | TG |
| 382 | 946.8836 | TG-57:2 | TG |
| 383 | 948.9012 | TG-57:1 | TG |
| 384 | 952.8344 | TG-58:6 | TG |
| 385 | 956.8646 | TG-58:4 | TG |
| 386 | 958.8865 | TG-58:3 | TG |
| 387 | 960.8916 | TG-58:2 | TG |
| 388 | 976.9298 | TG-59:1 | TG |
| 389 | 978.9465 | TG-59:0 | TG |
| 390 | 988.9258 | TG-60:2 | TG |
| 391 | 990.9436 | TG-60:1 | TG |
| 392 | 1014.942 | TG-62:3 | TG |
| 393 | 1016.962 | TG-62:2 | TG |

Negative mode targeted lipid list

| Index | exact.mass | ID | peak | Class |
| --- | --- | --- | --- | --- |
| 1 | 526.3521 | LPC O-16:1 |  | LPC-O |
| 2 | 554.3834 | LPC O-18:0 |  | LPC-O |
| 3 | 552.3669 | LPC O-18:1 | 1 | LPC-O |
| 4 | 552.3671 | LPC O-18:1 | 2 | LPC-O |
| 5 | 582.4139 | LPC O-20:0 |  | LPC-O |
| 6 | 636.4618 | LPC O-24:1 |  | LPC-O |
| 7 | 634.4457 | LPC O-24:2 |  | LPC-O |
| 8 | 436.2837 | LPE O-16:1 |  | LPE-O |
| 9 | 464.3146 | LPE O-18:1 |  | LPE-O |
| 10 | 462.2985 | LPE O-18:2 |  | LPE-O |
| 11 | 492.3449 | LPE O-20:1 |  | LPE-O |
| 12 | 764.5797 | PC O-32:0 |  | PC-O |
| 13 | 762.5646 | PC O-32:1 |  | PC-O |
| 14 | 790.5949 | PC O-34:1 |  | PC-O |
| 15 | 788.5808 | PC O-34:2 | 1 | PC-O |
| 16 | 788.5813 | PC O-34:2 | 2 | PC-O |
| 17 | 786.5646 | PC O-34:3 |  | PC-O |
| 18 | 816.6114 | PC O-36:2 |  | PC-O |
| 19 | 814.5961 | PC O-36:3 | 1 | PC-O |
| 20 | 814.5947 | PC O-36:3 | 2 | PC-O |
| 21 | 814.5949 | PC O-36:3 | 3 | PC-O |
| 22 | 812.5806 | PC O-36:4 |  | PC-O |
| 23 | 810.5651 | PC O-36:5 |  | PC-O |
| 24 | 840.611 | PC O-38:4 | 1 | PC-O |
| 25 | 840.6113 | PC O-38:4 | 2 | PC-O |
| 26 | 838.5955 | PC O-38:5 | 1 | PC-O |
| 27 | 838.5972 | PC O-38:5 | 2 | PC-O |
| 28 | 836.5804 | PC O-38:6 |  | PC-O |
| 29 | 834.5637 | PC O-38:7 |  | PC-O |
| 30 | 868.6436 | PC O-40:4 |  | PC-O |
| 31 | 866.6277 | PC O-40:5 |  | PC-O |
| 32 | 864.6101 | PC O-40:6 |  | PC-O |
| 33 | 894.6594 | PC O-42:5 |  | PC-O |
| 34 | 922.6912 | PC O-44:5 |  | PC-O |
| 35 | 920.6752 | PC O-44:6 |  | PC-O |
| 36 | 700.5289 | PE O-34:2 |  | PE-O |
| 37 | 698.5128 | PE O-34:3 |  | PE-O |
| 38 | 728.5596 | PE O-36:2 |  | PE-O |
| 39 | 726.5438 | PE O-36:3 |  | PE-O |
| 40 | 724.5276 | PE O-36:4 |  | PE-O |
| 41 | 722.5123 | PE O-36:5 |  | PE-O |
| 42 | 720.4961 | PE O-36:6 |  | PE-O |
| 43 | 736.527 | PE O-37:5 |  | PE-O |
| 44 | 754.5761 | PE O-38:3 |  | PE-O |
| 45 | 752.559 | PE O-38:4 |  | PE-O |
| 46 | 750.5428 | PE O-38:5 | 1 | PE-O |

| 47 | 750.5429 | PE O-38:5 | 3 | PE-O |
| --- | --- | --- | --- | --- |
| 48 | 750.5438 | PE O-38:5 | 2 | PE-O |
| 49 | 748.5265 | PE O-38:6 | 1 | PE-O |
| 50 | 748.527 | PE O-38:6 | 2 | PE-O |
| 51 | 746.5131 | PE O-38:7 |  | PE-O |
| 52 | 778.5747 | PE O-40:5 |  | PE-O |
| 53 | 776.5593 | PE O-40:6 |  | PE-O |
| 54 | 774.5435 | PE O-40:7 |  | PE-O |
| 55 | 772.5282 | PE O-40:8 |  | PE-O |
| 56 | 227.2018 | FA 14:0 |  | FA |
| 57 | 255.2332 | FA 16:0 |  | FA |
| 58 | 253.2174 | FA 16:1 |  | FA |
| 59 | 269.2488 | FA 17:0 |  | FA |
| 60 | 283.2643 | FA 18:0 |  | FA |
| 61 | 281.2494 | FA 18:1 |  | FA |
| 62 | 279.2334 | FA 18:2 |  | FA |
| 63 | 277.2172 | FA 18:3 | 2 | FA |
| 64 | 277.2174 | FA 18:3 | 1 | FA |
| 65 | 311.2958 | FA 20:0 |  | FA |
| 66 | 309.2804 | FA 20:1 |  | FA |
| 67 | 303.2331 | FA 20:4 |  | FA |
| 68 | 301.2177 | FA 20:5 |  | FA |
| 69 | 339.3271 | FA 22:0 |  | FA |
| 70 | 331.2643 | FA 22:4 |  | FA |
| 71 | 329.2486 | FA 22:5 | 1 | FA |
| 72 | 329.2485 | FA 22:5 | 2 | FA |
| 73 | 327.233 | FA 22:6 |  | FA |
| 74 | 365.3425 | FA 24:1 |  | FA |
| 75 | 744.5613 | HexCer 34:1-O2 |  | HexCer |
| 76 | 800.6254 | HexCer 38:1-O2 |  | HexCer |
| 77 | 828.6566 | HexCer 40:1-O2 |  | HexCer |
| 78 | 409.2351 | LPA 16:0 |  | LPA |
| 79 | 437.2681 | LPA 18:0 |  | LPA |
| 80 | 435.252 | LPA 18:1 |  | LPA |
| 81 | 433.2363 | LPA 18:2 |  | LPA |
| 82 | 459.2506 | LPA 20:3 |  | LPA |
| 83 | 457.2363 | LPA 20:4 |  | LPA |
| 84 | 481.2359 | LPA 22:6 |  | LPA |
| 85 | 512.2994 | LPC 14:0 |  | LPC |
| 86 | 526.3149 | LPC 15:0 |  | LPC |
| 87 | 540.3308 | LPC 16:0 | 2 | LPC |
| 88 | 540.3309 | LPC 16:0 | 1 | LPC |
| 89 | 538.3154 | LPC 16:1 |  | LPC |
| 90 | 554.3463 | LPC 17:0 |  | LPC |
| 91 | 552.3314 | LPC 17:1 |  | LPC |
| 92 | 568.3622 | LPC 18:0 |  | LPC |
| 93 | 568.3621 | LPC 18:0 |  | LPC |

| 94 | 566.3463 | LPC 18:1 |  | LPC |
| --- | --- | --- | --- | --- |
| 95 | 566.3466 | LPC 18:1 |  | LPC |
| 96 | 564.3309 | LPC 18:2 |  | LPC |
| 97 | 564.3304 | LPC 18:2 |  | LPC |
| 98 | 582.376 | LPC 19:0 |  | LPC |
| 99 | 582.3782 | LPC 19:0 |  | LPC |
| 100 | 596.3932 | LPC 20:0 |  | LPC |
| 101 | 594.3771 | LPC 20:1 |  | LPC |
| 102 | 592.3622 | LPC 20:2 |  | LPC |
| 103 | 590.3467 | LPC 20:3 |  | LPC |
| 104 | 588.331 | LPC 20:4 | 2 | LPC |
| 105 | 588.33 | LPC 20:4 | 1 | LPC |
| 106 | 616.3627 | LPC 22:4 |  | LPC |
| 107 | 614.346 | LPC 22:5 | 1 | LPC |
| 108 | 614.3459 | LPC 22:5 | 2 | LPC |
| 109 | 612.331 | LPC 22:6 |  | LPC |
| 110 | 652.4555 | LPC 24:0 |  | LPC |
| 111 | 452.2782 | LPE 16:0 | 2 | LPE |
| 112 | 452.2779 | LPE 16:0 | 1 | LPE |
| 113 | 480.3091 | LPE 18:0 | 2 | LPE |
| 114 | 480.3095 | LPE 18:0 | 1 | LPE |
| 115 | 478.2938 | LPE 18:1 |  | LPE |
| 116 | 476.2788 | LPE 18:2 | 2 | LPE |
| 117 | 476.2785 | LPE 18:2 | 1 | LPE |
| 118 | 502.2933 | LPE 20:3 |  | LPE |
| 119 | 500.2786 | LPE 20:4 |  | LPE |
| 120 | 528.3096 | LPE 22:4 |  | LPE |
| 121 | 526.2947 | LPE 22:5 |  | LPE |
| 122 | 509.2889 | LPG 18:1 |  | LPG |
| 123 | 507.272 | LPG 18:2 |  | LPG |
| 124 | 571.2899 | LPI 16:0 |  | LPI |
| 125 | 599.3203 | LPI 18:0 |  | LPI |
| 126 | 597.304 | LPI 18:1 |  | LPI |
| 127 | 524.2999 | LPS 18:0 |  | LPS |
| 128 | 522.2827 | LPS 18:1 |  | LPS |
| 129 | 750.5283 | PC 30:0 |  | PC |
| 130 | 778.5603 | PC 32:0 |  | PC |
| 131 | 776.5436 | PC 32:1 |  | PC |
| 132 | 774.5281 | PC 32:2 |  | PC |
| 133 | 788.5441 | PC 33:2 |  | PC |
| 134 | 806.5911 | PC 34:0 |  | PC |
| 135 | 804.5755 | PC 34:1 |  | PC |
| 136 | 802.5609 | PC 34:2 |  | PC |
| 137 | 800.5433 | PC 34:3 |  | PC |
| 138 | 800.5443 | PC 34:3 |  | PC |
| 139 | 798.5273 | PC 34:4 |  | PC |
| 140 | 816.5751 | PC 35:2 | 2 | PC |

| 141 | 816.5756 | PC 35:2 | 1 | PC |
| --- | --- | --- | --- | --- |
| 142 | 812.5436 | PC 35:4 |  | PC |
| 143 | 832.6069 | PC 36:1 |  | PC |
| 144 | 830.5914 | PC 36:2 |  | PC |
| 145 | 828.5754 | PC 36:3 | 1 | PC |
| 146 | 828.5762 | PC 36:3 | 2 | PC |
| 147 | 826.5604 | PC 36:4 | 1 | PC |
| 148 | 826.5587 | PC 36:4 | 2 | PC |
| 149 | 824.5441 | PC 36:5 | 1 | PC |
| 150 | 824.5447 | PC 36:5 | 2 | PC |
| 151 | 824.5436 | PC 36:5 | 3 | PC |
| 152 | 822.5289 | PC 36:6 |  | PC |
| 153 | 844.6072 | PC 37:2 |  | PC |
| 154 | 842.5912 | PC 37:3 |  | PC |
| 155 | 836.5436 | PC 37:6 |  | PC |
| 156 | 858.622 | PC 38:2 |  | PC |
| 157 | 856.6069 | PC 38:3 |  | PC |
| 158 | 854.5911 | PC 38:4 | 1 | PC |
| 159 | 854.5914 | PC 38:4 | 2 | PC |
| 160 | 854.5904 | PC 38:4 | 3 | PC |
| 161 | 852.5763 | PC 38:5 | 1 | PC |
| 162 | 852.5759 | PC 38:5 | 2 | PC |
| 163 | 850.5598 | PC 38:6 | 1 | PC |
| 164 | 850.5593 | PC 38:6 | 2 | PC |
| 165 | 848.5442 | PC 38:7 | 1 | PC |
| 166 | 848.5437 | PC 38:7 | 2 | PC |
| 167 | 864.575 | PC 39:6 |  | PC |
| 168 | 882.6227 | PC 40:4 | 1 | PC |
| 169 | 882.623 | PC 40:4 | 2 | PC |
| 170 | 880.6063 | PC 40:5 | 1 | PC |
| 171 | 880.607 | PC 40:5 | 2 | PC |
| 172 | 878.5912 | PC 40:6 | 1 | PC |
| 173 | 878.5897 | PC 40:6 | 2 | PC |
| 174 | 876.5743 | PC 40:7 |  | PC |
| 175 | 874.5613 | PC 40:8 |  | PC |
| 176 | 898.5605 | PC 42:10 |  | PC |
| 177 | 716.5233 | PE 34:1 |  | PE |
| 178 | 714.5075 | PE 34:2 |  | PE |
| 179 | 744.5549 | PE 36:1 |  | PE |
| 180 | 742.5383 | PE 36:2 |  | PE |
| 181 | 740.5228 | PE 36:3 |  | PE |
| 182 | 738.5077 | PE 36:4 | 1 | PE |
| 183 | 738.5073 | PE 36:4 | 2 | PE |
| 184 | 768.5548 | PE 38:3 |  | PE |
| 185 | 766.5382 | PE 38:4 |  | PE |
| 186 | 764.5226 | PE 38:5 | 1 | PE |
| 187 | 764.5237 | PE 38:5 | 2 | PE |

| 188 | 762.507 | PE 38:6 | PE |
| --- | --- | --- | --- |
| 189 | 794.5702 | PE 40:4 | PE |
| 190 | 792.5533 | PE 40:5 | PE |
| 191 | 790.5383 | PE 40:6 | PE |
| 192 | 807.5031 | PI 32:1 | PI |
| 193 | 835.5353 | PI 34:1 | PI |
| 194 | 833.5187 | PI 34:2 | PI |
| 195 | 863.5644 | PI 36:1 | PI |
| 196 | 861.5504 | PI 36:2 | PI |
| 197 | 859.5335 | PI 36:3 | PI |
| 198 | 857.5181 | PI 36:4 | PI |
| 199 | 887.5651 | PI 38:3 | PI |
| 200 | 885.5505 | PI 38:4 | PI |
| 201 | 883.5335 | PI 38:5 | PI |
| 202 | 881.5185 | PI 38:6 | PI |
| 203 | 909.5477 | PI 40:6 | PI |
| 204 | 788.5445 | PS 36:1 | PS |
| 205 | 810.5275 | PS 38:4 | PS |

**Supplementary Table 5:** Features (Clinical and demograhic, immunotypes and proteins) assoiated with LPC-O-16:0, PC-O-30:0 and ChoE-18:3 (Spearman’s rho < -0.4 or > 0.4, p < 0.05)

| Dataset | Correlated feature | Spearm an's rho | p | n | Moderate vs severe covid q value |
| --- | --- | --- | --- | --- | --- |
| LPC O -16:0 |  |  |  |  |  |
| Clinical and | ards_ever | -0.4 | 0.00 | 67 | 7.56E-24 |
| demographic |  |  | 087 |  |  |
|  | daysaliveventfree_in28 | 0.51 | 7.77 | 55 | 2.56E-18 |
|  |  |  | E- |  |  |
|  |  |  | 05 |  |  |
|  | hsCRP | -0.58 | 0.01 | 18 | 1.15E-05 |
|  |  |  | 108 |  |  |
|  |  |  | 4 |  |  |
|  | intubated_yn | -0.43 | 0.00 | 63 | 4.12E-22 |
|  |  |  | 046 |  |  |
|  |  |  | 8 |  |  |
|  | ordinal_d7 | -0.4 | 0.00 | 67 | 4.82E-26 |
|  |  |  | 088 |  |  |
|  |  |  | 7 |  |  |
|  | ordinal_enrollment | -0.4 | 0.00 | 67 | 2.58E-46 |
|  |  |  | 080 |  |  |
|  |  |  | 3 |  |  |
|  | pct | -0.44 | 0.00 | 40 | 1.10E-05 |
|  |  |  | 487 |  |  |
|  |  |  | 4 |  |  |
| Immunotype | percOf_CD4_CD4EMRA | -0.47 | 0.00 | 40 | 0.751329993 |
|  |  |  | 246 |  |  |
|  |  |  | 7 |  |  |
|  | percOf_CD4_CD4NNKI67pos | -0.46 | 0.00 | 40 | 0.046842496 |
|  |  |  | 283 |  |  |
|  |  |  | 1 |  |  |
|  | percOf_CD4_KI67pos | -0.47 | 0.00 | 40 | 0.08582355 |
|  |  |  | 237 |  |  |
|  |  |  | 3 |  |  |
|  | percOf_CD4_TBETpos | -0.45 | 0.00  401 | 40 | 0.548651911 |
|  | percOf_CD4CM_KI67pos | -0.43 | 0.00 | 40 | 0.012293841 |
|  |  |  | 563 |  |  |
|  |  |  | 8 |  |  |
|  | percOf_CD4cTfh_CD4acTfh | -0.44 | 0.00 | 40 | 0.025195859 |
|  |  |  | 416 |  |  |
|  |  |  | 8 |  |  |
|  | percOf_CD4cTfh_HLADRposCD38pos | -0.52 | 0.00 | 40 | 0.012241891 |

|  |  |  | 062  1 |  |  |
| --- | --- | --- | --- | --- | --- |
|  | percOf_CD4cTfh_KI67pos | -0.53 | 0.00 | 40 | 0.301400751 |
|  |  |  | 037 |  |  |
|  |  |  | 9 |  |  |
|  | percOf_CD4EM1_HLADRposCD38pos | -0.43 | 0.00 | 40 | 0.000783423 |
|  |  |  | 552 |  |  |
|  |  |  | 3 |  |  |
|  | percOf_CD4EM1_KI67pos | -0.58 | 7.40 | 40 | 0.026755803 |
|  |  |  | E- |  |  |
|  |  |  | 05 |  |  |
|  | percOf_CD4nonNaive_KI67pos | -0.42 | 0.00 | 40 | 0.031992847 |
|  |  |  | 667 |  |  |
|  |  |  | 2 |  |  |
|  | percOf_CD4nonNaive_TBETpos | -0.47 | 0.00 | 40 | 0.544146679 |
|  |  |  | 238 |  |  |
|  |  |  | 8 |  |  |
|  | percOf_CD8_CD8EM1 | 0.4 | 0.01 | 40 | 0.087303203 |
|  |  |  | 122 |  |  |
|  |  |  | 5 |  |  |
|  | percOf_CD8_CD8EMRA | -0.48 | 0.00 | 40 | 0.384334434 |
|  |  |  | 165 |  |  |
|  |  |  | 3 |  |  |
|  | percOf_CD8_TBETpos | -0.48 | 0.00 | 40 | 0.621801498 |
|  |  |  | 186 |  |  |
|  |  |  | 2 |  |  |
|  | percOf_CD8nonNaive_TBETpos | -0.47 | 0.00 | 40 | 0.535187498 |
|  |  |  | 247 |  |  |
|  |  |  | 2 |  |  |
|  | percOf_Live_CD4EMRA | -0.41 | 0.00 | 40 | 0.532803297 |
|  |  |  | 863 |  |  |
|  |  |  | 2 |  |  |
|  | percOf_Live_CD8EMRA | -0.43 | 0.00 | 40 | 0.906272113 |
|  |  |  | 547 |  |  |
|  |  |  | 8 |  |  |
|  | umap_component2 | -0.4 | 0.01  127 | 39 | 0.513113445 |
| Proteins | CAM_P01033 Metalloproteinase inhibitor 1 (TIMP1) | -0.4 | 0.00 | 67 | 0.000118445 |
|  |  |  | 069 |  |  |
|  |  |  | 7 |  |  |
|  | CVD2_P05231 Interleukin-6 (IL6) | -0.44 | 0.00 | 67 | 0.00810288 |
|  |  |  | 017 |  |  |
|  |  |  | 2 |  |  |
|  | CVD2_P07711 Cathepsin L1 (CTSL1) | -0.45 | 0.00 | 67 | 8.22E-07 |
|  |  |  | 014 |  |  |
|  |  |  | 5 |  |  |
|  | CVD2_P09237 Matrix metalloproteinase-7 | -0.44 | 0.00 | 67 | 0.032766248 |

|  | (MMP-7) |  | 023  4 |  |  |
| --- | --- | --- | --- | --- | --- |
|  | CVD3_O00300 Osteoprotegerin (OPG) | -0.41 | 0.00 | 67 | 0.000215128 |
|  |  |  | 049 |  |  |
|  |  |  | 9 |  |  |
|  | CVD3_P20160 Azurocidin (AZU1 | -0.42 | 0.00 | 67 | 7.12E-06 |
|  |  |  | 047 |  |  |
|  |  |  | 6 |  |  |
|  | DEV_Q96QR1 Secretoglobin family 3A member 1 (SCGB3A1) | 0.4 | 0.00 | 67 | 0.000262582 |
|  |  |  | 067 |  |  |
|  |  |  | 9 |  |  |
|  | INF_O00300 Osteoprotegerin (OPG) | -0.43 | 0.00 | 67 | 0.000558337 |
|  |  |  | 028 |  |  |
|  |  |  | 4 |  |  |
|  | INF_P05231 Interleukin-6 (IL6) | -0.44 | 0.00 | 67 | 0.007025703 |
|  |  |  | 020 |  |  |
|  |  |  | 4 |  |  |
|  | INF_P10145 Interleukin-8 (IL-8) | -0.42 | 0.00 | 67 | 0.00036114 |
|  |  |  | 037 |  |  |
|  |  |  | 2 |  |  |
|  | INF_P14210 Hepatocyte growth factor (HGF) | -0.42 | 0.00 | 67 | 7.29E-07 |
|  |  |  | 042 |  |  |
|  |  |  | 1 |  |  |
|  | INF_P15018 Leukemia inhibitory factor (LIF) | -0.57 | 3.74 | 67 | 0.000706887 |
|  |  |  | E- |  |  |
|  |  |  | 07 |  |  |
|  | INF_P55773 C-C motif chemokine 23 (CCL23) | -0.49 | 2.58 | 67 | 7.12E-06 |
|  |  |  | E- |  |  |
|  |  |  | 05 |  |  |
|  | INF_P78556 C-C motif chemokine 20 (CCL20) | -0.51 | 9.28 | 67 | 0.000218196 |
|  |  |  | E- |  |  |
|  |  |  | 06 |  |  |
|  | INF_Q13007 Interleukin-24 (IL-24) | -0.42 | 0.00 | 67 | 7.42E-05 |
|  |  |  | 034 |  |  |
|  |  |  | 5 |  |  |
|  | IRE_P05231 Interleukin-6 (IL6) | -0.43 | 0.00 | 67 | 0.00956383 |
|  |  |  | 030 |  |  |
|  |  |  | 1 |  |  |
|  | ODA_P01258 Calcitonin (CALCA) | -0.41 | 0.00 | 67 | 7.10E-06 |
|  |  |  | 049 |  |  |
|  |  |  | 1 |  |  |
| sPLA2 | P14555 sPLA2 | -0.43 | 0.00 | 36 | 0.133848614 |
|  |  |  | 822 |  |  |
|  |  |  | 5 |  |  |
|  |  |  |  |  |  |
| PC O-30:0 |  |  |  |  |  |

| Immunotype | percOf_Bcell_EOMESpos | -0.4 | 0.01  035 | 40 | 0.050295441 |
| --- | --- | --- | --- | --- | --- |
|  | percOf_CD4cTfh_CD4acTfh | -0.48 | 0.00 | 40 | 0.025195859 |
|  |  |  | 156 |  |  |
|  |  |  | 7 |  |  |
|  | percOf_CD4EM1_HLADRposCD38pos | -0.45 | 0.00 | 40 | 0.000783423 |
|  |  |  | 339 |  |  |
|  |  |  | 9 |  |  |
|  | percOf_CD4EM1_KI67pos | -0.42 | 0.00  758 | 40 | 0.026755803 |
|  | percOf_CD8EM1_HLADRposCD38pos | -0.43 | 0.00 | 40 | 0.012293841 |
|  |  |  | 536 |  |  |
|  |  |  | 6 |  |  |
|  | percOf_CD8EM1_KI67pos | -0.43 | 0.00 | 40 | 0.114591817 |
|  |  |  | 508 |  |  |
|  |  |  | 4 |  |  |
|  |  | -0.42 | 0.00 | 40 | 0.025674422 |
|  | percOf_CD8RAposCD27posR7neg_KI67pos |  | 680 |  |  |
|  |  |  | 8 |  |  |
|  |  | -0.46 | 0.00 | 40 | 0.037329622 |
|  | percOf_CD8RAposCD27posR7posCD95pos |  | 283 |  |  |
|  | _HLADRposCD38pos |  | 3 |  |  |
|  |  | -0.56 | 0.00 | 40 | 0.031992847 |
|  | percOf_CD8RAposCD27posR7posCD95pos |  | 016 |  |  |
|  | _KI67pos |  | 6 |  |  |
|  | percOf_Live_CD4EM2 | 0.41 | 0.00 | 40 | 0.041352462 |
|  |  |  | 861 |  |  |
|  |  |  | 2 |  |  |
|  | percOf_Live_CD4nonNaive | 0.4 | 0.01 | 40 | 1.48E-07 |
|  |  |  | 038 |  |  |
|  |  |  | 9 |  |  |
|  | umap_component1 | -0.41 | 0.00 | 39 | 0.000317432 |
|  |  |  | 982 |  |  |
|  |  |  | 8 |  |  |
| Protein | CAM_Q9BXR6 Complement factor H- related protein 5 (CFHR5) | -0.45 | 0.00 | 67 | 0.549657525 |
|  |  |  | 011 |  |  |
|  |  |  | 9 |  |  |
|  | CVD3_P01130 Low-density lipoprotein receptor (LDL receptor) | -0.42 | 0.00 | 67 | 0.000462016 |
|  |  |  | 046 |  |  |
|  |  |  | 1 |  |  |
|  | CVD3_P20160 Azurocidin (AZU1 | -0.4 | 0.00 | 67 | 7.12E-06 |
|  |  |  | 074 |  |  |
|  |  |  | 4 |  |  |
|  | INF_P13232 Interleukin-7 (IL-7) | -0.48 | 4.24 | 67 | 7.46E-05 |
|  |  |  | E- |  |  |
|  |  |  | 05 |  |  |

|  | INF_P14210 Hepatocyte growth factor (HGF) | -0.42 | 0.00  040  4 | 67 | 7.29E-07 |
| --- | --- | --- | --- | --- | --- |
|  | INF_P80511 Protein S100-A12 (EN-RAGE ) | -0.48 | 3.55  E- 05 | 67 | 1.21E-08 |
|  | IRE_P78362 SRSF protein kinase 2 (SRPK2) | -0.4 | 0.00  083  2 | 67 | 0.000194136 |
|  | IRE_Q05516 Zinc finger and BTB domain- containing protein 16 (ZBTB16) | -0.4 | 0.00  073  4 | 67 | 2.36E-05 |
|  | IRE_Q9GZT9 Egl nine homolog 1 (EGLN1) | -0.41 | 0.00  059 | 67 | 0.031265046 |
|  | MET_P27695 DNA-(apurinic or apyrimidinic site) lyase (APEX1) | -0.46 | 8.20  E- 05 | 67 | 0.000162327 |
|  | MET_Q02790 Peptidyl-prolyl cis-trans isomerase FKBP4 (FKBP4) | -0.41 | 0.00  051  1 | 67 | 0.001680452 |
|  | ODA_O60934 Nibrin (NBN) | -0.42 | 0.00  041 | 67 | 0.001934367 |
|  | ODA_P09769 Tyrosine-protein kinase Fgr (FGR) | -0.47 | 5.65  E-  05 | 67 | 2.10E-06 |
|  | ODA_Q02880 DNA topoisomerase 2-beta (TOP2B) | -0.41 | 0.00  060  3 | 67 | 0.000215128 |
|  | ODA_Q9HAW4 Claspin (CLSPN) | -0.45 | 0.00  015  5 | 67 | 0.001265811 |
|  |  |  |  |  |  |
| ChoE-18:3 |  |  |  |  |  |
| Immunotype | percOf_Bcell_CD138pos | -0.45 | 0.00  342  9 | 40 | 0.062190734 |
|  | percOf_Bcell_CD39pos | -0.46 | 0.00  283  3 | 40 | 0.114591817 |
|  | percOf_Bcell_HLADRpos | 0.45 | 0.00  390  8 | 40 | 0.009618759 |
|  | percOf_Bcell_KI67pos | -0.43 | 0.00  507  4 | 40 | 0.105547477 |
|  | percOf_BcellnotPB_CD138pos | -0.41 | 0.00 | 40 | 0.982727613 |

|  |  |  | 824  7 |  |  |
| --- | --- | --- | --- | --- | --- |
| Proteins | CVD2_O00182 Galectin-9 (Gal-9) | -0.4 | 0.00  092  5 | 67 | 0.000460426 |
|  | CVD2_Q9NQ25 SLAM family member 7 (SLAMF7) | -0.44 | 0.00  017 | 67 | 0.945889856 |
|  | CVD3_P35247 Pulmonary surfactant- associated protein D (PSP-D) | -0.41 | 0.00  048  8 | 67 | 7.60E-05 |
|  | DEV_Q96RD9 Fc receptor-like protein 5 (FCRL5) | -0.44 | 0.00  022  7 | 67 | 0.211300783 |
|  | DEV_Q9NZV1 Cysteine-rich motor neuron 1 protein (CRIM1) | -0.41 | 0.00  064  7 | 67 | 0.040625923 |
|  | DEV_Q9Y279 V-set and immunoglobulin domain-containing protein 4 (VSIG4) | -0.4 | 0.00  068  6 | 67 | 2.47E-06 |
|  | INF_Q5T4W7 Artemin (ARTN) | -0.42 | 0.00  041  2 | 67 | 0.042728343 |
|  | INF_Q9NZQ7 Programmed cell death 1 ligand 1 (PD-L1) | -0.43 | 0.00  033  1 | 67 | 0.001304588 |
|  | IRE_P27540 Aryl hydrocarbon receptor nuclear translocator (ARNT) | -0.43 | 0.00  028  7 | 67 | 0.598459965 |
|  | IRE_Q8IU57 Interferon lambda receptor 1 (IFNLR1) | -0.44 | 0.00  022  2 | 67 | 0.050771359 |
|  | N/A_Q9UNK4 PLA2G2D | -0.56 | 0.04  872  9 | 13 | 0.917641149 |

**Supplementary Table 6:** Functional enrichment analysis of biological processes from proteins significantly associated (Spearman’s rho < -0.4 or > 0.4, p < 0.05) with LPC-O-16:0, PC-O-30:0 and ChoE-18:3

| Goter m | term description | obser ved gene count | backg round gene count | stre ngt h | false disc over y rate | Observed proteins |
| --- | --- | --- | --- | --- | --- | --- |
| LPC- O- 16:0 |  |  |  |  |  |  |
| GO:0 | Cell | 7 | 204 | 1.68 | 6.32 | HGF,AZU1,CXCL8,CALCA,CCL20,IL6,CCL23 |
| 06032 | chemotaxis |  |  |  | E-07 |  |
| 6 |  |  |  |  |  |  |
| GO:0 | Cytokine- | 9 | 678 | 1.27 | 7.71 | TIMP1,HGF,LIF,TNFRSF11B,CXCL8,CCL20,IL24,I |
| 01922 | mediated |  |  |  | E-07 | L6,CCL23 |
| 1 | signaling |  |  |  |  |  |
|  | pathway |  |  |  |  |  |
| GO:0 | Cellular | 10 | 1013 | 1.14 | 7.71 | TIMP1,HGF,LIF,TNFRSF11B,CXCL8,CALCA,CCL |
| 07134 | response to |  |  |  | E-07 | 20,IL24,IL6,CCL23 |
| 5 | cytokine |  |  |  |  |  |
|  | stimulus |  |  |  |  |  |
| GO:0 | Myeloid | 6 | 123 | 1.83 | 7.71 | AZU1,CXCL8,CALCA,CCL20,IL6,CCL23 |
| 09752 | leukocyte |  |  |  | E-07 |  |
| 9 | migration |  |  |  |  |  |
| GO:0 | Leukocyte | 6 | 142 | 1.77 | 1.03 | AZU1,CXCL8,CALCA,CCL20,IL6,CCL23 |
| 03059 | chemotaxis |  |  |  | E-06 |  |
| 5 |  |  |  |  |  |  |
| GO:0 | Cellular | 12 | 2919 | 0.76 | 1.54 | TIMP1,HGF,AZU1,LIF,TNFRSF11B,CXCL8,CALC |
| 07088 | response to |  |  |  | E-05 | A,CTSL,CCL20,IL24,IL6,CCL23 |
| 7 | chemical |  |  |  |  |  |
|  | stimulus |  |  |  |  |  |
| GO:0 | Cellular | 11 | 2369 | 0.81 | 3.45 | TIMP1,HGF,LIF,TNFRSF11B,CXCL8,CALCA,CTSL |
| 07131 | response to |  |  |  | E-05 | ,CCL20,IL24,IL6,CCL23 |
| 0 | organic |  |  |  |  |  |
|  | substance |  |  |  |  |  |
| GO:0 | Monocyte | 4 | 43 | 2.11 | 3.83 | CALCA,CCL20,IL6,CCL23 |
| 00254 | chemotaxis |  |  |  | E-05 |  |
| 8 |  |  |  |  |  |  |
| GO:0 | Inflammato | 7 | 515 | 1.28 | 3.83 | TIMP1,AZU1,CXCL8,CALCA,CCL20,IL6,CCL23 |
| 00695 | ry response |  |  |  | E-05 |  |
| 4 |  |  |  |  |  |  |

| GO:0 00193  4 | Positive regulation of protein phosphoryl ation | 8 | 1019 | 1.04 | 0.00  011 | HGF,AZU1,LIF,CALCA,CCL20,IL24,IL6,CCL23 |
| --- | --- | --- | --- | --- | --- | --- |
| GO:0 00716  5 | Signal transductio n | 13 | 4876 | 0.57 | 0.00  013 | TIMP1,HGF,AZU1,LIF,SCGB3A1,TNFRSF11B,CXC L8,CALCA,CTSL,CCL20,IL24,IL6,CCL23 |
| GO:0 04577  9 | Negative regulation of bone resorption | 3 | 14 | 2.48 | 0.00  015 | TNFRSF11B,CALCA,IL6 |
| GO:0 05171  6 | Cellular response to stimulus | 14 | 6489 | 0.48 | 0.00  015 | TIMP1,HGF,AZU1,LIF,MMP7,SCGB3A1,TNFRSF1 1B,CXCL8,CALCA,CTSL,CCL20,IL24,IL6,CCL23 |
| GO:0 00960  5 | Response to external stimulus | 10 | 2310 | 0.78 | 0.00  02 | HGF,AZU1,MMP7,TNFRSF11B,CXCL8,CALCA,C CL20,IL24,IL6,CCL23 |
| GO:0 07135  6 | Cellular response to tumor necrosis factor | 5 | 245 | 1.46 | 0.00  028 | TNFRSF11B,CXCL8,CALCA,CCL20,CCL23 |
| GO:0 00695  9 | Humoral immune response | 5 | 275 | 1.41 | 0.00  042 | AZU1,CXCL8,CALCA,CCL20,IL6 |
| GO:0 04001  2 | Regulation of locomotion | 7 | 969 | 1 | 0.00  071 | TIMP1,HGF,AZU1,CXCL8,CCL20,IL24,IL6 |
| GO:0 00961  7 | Response to bacterium | 6 | 634 | 1.12 | 0.00  097 | AZU1,CXCL8,CALCA,CCL20,IL24,IL6 |
| GO:0 06500  9 | Regulation of molecular function | 12 | 4913 | 0.53 | 0.00  11 | TIMP1,HGF,AZU1,LIF,SCGB3A1,TNFRSF11B,CXC L8,CALCA,CCL20,IL24,IL6,CCL23 |
| GO:0 00695  5 | Immune response | 8 | 1588 | 0.85 | 0.00  12 | AZU1,LIF,CXCL8,CALCA,CTSL,CCL20,IL6,CCL23 |
| GO:0 01973  0 | Antimicrob ial humoral response | 4 | 160 | 1.54 | 0.00  13 | AZU1,CXCL8,CALCA,CCL20 |
| GO:0 | Myeloid | 2 | 5 | 2.75 | 0.00 | CTSL,IL6 |

| 03302  8 | cell apoptotic process |  |  |  | 26 |  |
| --- | --- | --- | --- | --- | --- | --- |
| GO:0 05073  1 | Positive regulation of peptidyl- tyrosine  phosphoryl ation | 4 | 196 | 1.46 | 0.00  26 | HGF,LIF,IL24,IL6 |
| GO:0 05170  7 | Response to other organism | 7 | 1256 | 0.89 | 0.00  26 | AZU1,CXCL8,CALCA,CCL20,IL24,IL6,CCL23 |
| GO:0 00692  5 | Inflammato ry cell  apoptotic process | 2 | 6 | 2.67 | 0.00  29 | CTSL,IL6 |
| GO:0 02261  7 | Extracellul ar matrix disassembl y | 3 | 66 | 1.8 | 0.00  32 | TIMP1,MMP7,CTSL |
| GO:0 03033  4 | Regulation of cell  migration | 6 | 865 | 0.99 | 0.00  34 | TIMP1,HGF,CXCL8,CCL20,IL24,IL6 |
| GO:0 04253  1 | Positive regulation of tyrosine phosphoryl ation of stat protein | 3 | 68 | 1.79 | 0.00  34 | LIF,IL24,IL6 |
| GO:0 05079  3 | Regulation of developme ntal process | 9 | 2648 | 0.68 | 0.00  34 | TIMP1,HGF,LIF,SCGB3A1,TNFRSF11B,CXCL8,CA LCA,CTSL,IL6 |
| GO:0 03226  8 | Regulation of cellular protein metabolic process | 9 | 2693 | 0.67 | 0.00  37 | TIMP1,HGF,AZU1,LIF,CALCA,CCL20,IL24,IL6,C CL23 |
| GO:0 03059  3 | Neutrophil chemotaxis | 3 | 74 | 1.75 | 0.00  41 | CXCL8,CCL20,CCL23 |
| GO:0 | Regulation | 9 | 2740 | 0.66 | 0.00 | TIMP1,HGF,AZU1,LIF,CXCL8,CALCA,CCL20,IL2 |

| 03287  9 | of localization |  |  |  | 42 | 4,IL6 |
| --- | --- | --- | --- | --- | --- | --- |
| GO:0 07009  8 | Chemokine  -mediated signaling pathway | 3 | 80 | 1.72 | 0.00  47 | CXCL8,CCL20,CCL23 |
| GO:0 04341  0 | Positive regulation of mapk cascade | 5 | 543 | 1.11 | 0.00  49 | HGF,LIF,CCL20,IL6,CCL23 |
| GO:2 00002  6 | Regulation of multicellul ar organismal developme nt | 8 | 2096 | 0.73 | 0.00  49 | TIMP1,HGF,LIF,TNFRSF11B,CXCL8,CALCA,CTSL  ,IL6 |
| GO:0 04001  7 | Positive regulation of locomotion | 5 | 562 | 1.09 | 0.00  53 | HGF,AZU1,CXCL8,CCL20,IL6 |
| GO:0 03164  0 | Killing of  cells of other organism | 3 | 91 | 1.66 | 0.00  58 | AZU1,CXCL8,CCL20 |
| GO:0 04274  2 | Defense response to bacterium | 4 | 277 | 1.3 | 0.00  58 | AZU1,CALCA,CCL20,IL6 |
| GO:0 05082  9 | Defense response to gram- negative bacterium | 3 | 98 | 1.63 | 0.00  69 | AZU1,CALCA,IL6 |
| GO:0 04858  4 | Positive regulation of response to stimulus | 8 | 2257 | 0.69 | 0.00  73 | HGF,AZU1,LIF,CXCL8,CCL20,IL24,IL6,CCL23 |
| GO:0 00996  6 | Regulation of signal transductio n | 9 | 3107 | 0.61 | 0.00  86 | TIMP1,HGF,LIF,CXCL8,CALCA,CCL20,IL24,IL6,C CL23 |
| GO:0 04212  7 | Regulation of cell  population | 7 | 1642 | 0.78 | 0.00  86 | TIMP1,LIF,SCGB3A1,CXCL8,IL24,IL6,CCL23 |

|  | proliferatio n |  |  |  |  |  |
| --- | --- | --- | --- | --- | --- | --- |
| GO:0 02260  3 | Regulation of anatomical structure morphogen esis | 6 | 1095 | 0.88 | 0.00  89 | HGF,LIF,SCGB3A1,TNFRSF11B,CXCL8,IL6 |
| GO:0 04565  1 | Positive regulation of macrophag e differentiat ion | 2 | 15 | 2.27 | 0.00  9 | LIF,CALCA |
| GO:0 05093  0 | Induction of positive chemotaxis | 2 | 15 | 2.27 | 0.00  9 | AZU1,CXCL8 |
| GO:0 06184  4 | Antimicrob ial humoral immune response mediated by antimicrobi al peptide | 3 | 113 | 1.57 | 0.00  96 | CXCL8,CALCA,CCL20 |
| GO:0 04858  3 | Regulation of response to stimulus | 10 | 4114 | 0.53 | 0.00  99 | TIMP1,HGF,AZU1,LIF,CXCL8,CALCA,CCL20,IL2 4,IL6,CCL23 |
| GO:0 03019  8 | Extracellul ar matrix organizatio n | 4 | 338 | 1.22 | 0.01  07 | TIMP1,MMP7,TNFRSF11B,CTSL |
| GO:0 05123  9 | Regulation of multicellul ar organismal process | 9 | 3227 | 0.59 | 0.01  07 | TIMP1,HGF,AZU1,LIF,TNFRSF11B,CXCL8,CALC A,CTSL,IL6 |
| GO:0 00828  5 | Negative regulation of cell  population proliferatio | 5 | 696 | 1 | 0.01  15 | LIF,CXCL8,IL24,IL6,CCL23 |

|  | n |  |  |  |  |  |
| --- | --- | --- | --- | --- | --- | --- |
| GO:0 01993  2 | Second- messenger- mediated signaling | 4 | 354 | 1.2 | 0.01  22 | AZU1,CXCL8,CALCA,CCL20 |
| GO:0 04851  9 | Negative regulation of biological process | 11 | 5389 | 0.46 | 0.01  33 | TIMP1,HGF,AZU1,LIF,SCGB3A1,TNFRSF11B,CXC L8,CALCA,IL24,IL6,CCL23 |
| GO:0 00268  7 | Positive regulation of leukocyte migration | 3 | 144 | 1.46 | 0.01  69 | CXCL8,CCL20,IL6 |
| GO:0 03313  5 | Regulation of peptidyl- serine  phosphoryl ation | 3 | 146 | 1.46 | 0.01  74 | HGF,LIF,IL6 |
| GO:0 03558  4 | Calcium- mediated signaling using intracellula r calcium source | 2 | 24 | 2.07 | 0.01  75 | AZU1,CCL20 |
| GO:0 04852  2 | Positive regulation of cellular process | 11 | 5579 | 0.44 | 0.01  75 | TIMP1,HGF,AZU1,LIF,SCGB3A1,CXCL8,CALCA, CCL20,IL24,IL6,CCL23 |
| GO:0 05092  1 | Positive regulation of chemotaxis | 3 | 147 | 1.46 | 0.01  75 | AZU1,CXCL8,IL6 |
| GO:0 07188  7 | Leukocyte apoptotic process | 2 | 26 | 2.03 | 0.01  98 | CTSL,IL6 |
| GO:0 04871  0 | Regulation of astrocyte differentiat ion | 2 | 27 | 2.02 | 0.02  08 | LIF,IL6 |
| GO:0 | Calcium- | 3 | 165 | 1.41 | 0.02 | AZU1,CXCL8,CCL20 |

| 01972  2 | mediated signaling |  |  |  | 26 |  |
| --- | --- | --- | --- | --- | --- | --- |
| GO:0 05109  4 | Positive regulation of developme ntal process | 6 | 1389 | 0.78 | 0.02  51 | HGF,LIF,SCGB3A1,CXCL8,CALCA,IL6 |
| GO:0 07134  7 | Cellular response to interleukin  -1 | 3 | 174 | 1.38 | 0.02  59 | CXCL8,CCL20,CCL23 |
| GO:0 00181  9 | Positive regulation of cytokine production | 4 | 461 | 1.08 | 0.02  72 | HGF,AZU1,CALCA,IL6 |
| GO:0 00756  5 | Female pregnancy | 3 | 183 | 1.36 | 0.02  95 | LIF,MMP7,CALCA |
| GO:0 00971  9 | Response to endogenou s stimulus | 6 | 1447 | 0.76 | 0.03  02 | TIMP1,TNFRSF11B,CXCL8,CALCA,CTSL,IL6 |
| GO:0 07122  2 | Cellular response to lipopolysac charide | 3 | 185 | 1.36 | 0.03  02 | CXCL8,IL24,IL6 |
| GO:0 09854  2 | Defense response to other organism | 5 | 900 | 0.89 | 0.03  02 | AZU1,CALCA,CCL20,IL6,CCL23 |
| GO:0 04852  3 | Negative regulation of cellular process | 10 | 4874 | 0.46 | 0.03  35 | TIMP1,HGF,AZU1,LIF,SCGB3A1,CXCL8,CALCA,I L24,IL6,CCL23 |
| GO:0 03027  8 | Regulation of ossification | 3 | 197 | 1.33 | 0.03  48 | HGF,CALCA,IL6 |
| GO:0 00268  4 | Positive regulation of immune system process | 5 | 949 | 0.87 | 0.03  63 | LIF,CXCL8,CALCA,CCL20,IL6 |

| GO:0 00756  6 | Embryo implantatio n | 2 | 41 | 1.83 | 0.03  99 | LIF,CALCA |
| --- | --- | --- | --- | --- | --- | --- |
| GO:0 03033  5 | Positive regulation of cell  migration | 4 | 522 | 1.03 | 0.03  99 | HGF,CXCL8,CCL20,IL6 |
| GO:0 03057  4 | Collagen catabolic process | 2 | 43 | 1.81 | 0.04  3 | MMP7,CTSL |
| GO:0 04559  7 | Positive regulation of cell  differentiat ion | 5 | 993 | 0.85 | 0.04  3 | HGF,LIF,SCGB3A1,CALCA,IL6 |
| GO:0 04574  4 | Negative regulation of g  protein- coupled receptor signaling pathway | 2 | 45 | 1.79 | 0.04  55 | CXCL8,CALCA |
|  |  |  |  |  |  |  |
| PC-  O- 30:0 |  |  |  |  |  |  |
|  |  |  |  |  |  |  |
| GO:0 01060  4 | Positive regulation of macromole cule metabolic process | 12 | 3600 | 0.64 | 0.00  51 | APEX1,HGF,AZU1,IL7,NBN,CLSPN,ZBTB16,EGL N1,S100A12,FGR,SRPK2,LDLR |
| GO:0 04851  8 | Positive regulation of biological process | 14 | 6112 | 0.48 | 0.00  58 | APEX1,HGF,AZU1,CFHR5,IL7,NBN,CLSPN,ZBTB 16,EGLN1,S100A12,FGR,SRPK2,TOP2B,LDLR |
| GO:0 04851  9 | Negative regulation  of biological | 13 | 5389 | 0.5 | 0.00  99 | FKBP4,APEX1,HGF,AZU1,IL7,NBN,CLSPN,ZBTB 16,EGLN1,FGR,SRPK2,TOP2B,LDLR |

|  | process |  |  |  |  |  |
| --- | --- | --- | --- | --- | --- | --- |
| GO:0 03367  4 | Positive regulation of kinase activity | 6 | 624 | 1.1 | 0.01  09 | HGF,AZU1,NBN,CLSPN,S100A12,FGR |
| GO:0 04317  0 | Macromole cule metabolic process | 13 | 6137 | 0.44 | 0.02  78 | FKBP4,APEX1,HGF,AZU1,NBN,CLSPN,ZBTB16,E GLN1,S100A12,FGR,SRPK2,TOP2B,LDLR |
| GO:0 01922  0 | Regulation of phosphate metabolic process | 8 | 1816 | 0.76 | 0.03  17 | HGF,AZU1,IL7,NBN,CLSPN,S100A12,FGR,LDLR |
| GO:0 03132  5 | Positive regulation of cellular metabolic process | 10 | 3413 | 0.58 | 0.04  3 | APEX1,HGF,AZU1,NBN,CLSPN,ZBTB16,EGLN1,S 100A12,FGR,LDLR |
| GO:0 00193  2 | Regulation of protein phosphoryl ation | 7 | 1459 | 0.8 | 0.04  4 | HGF,AZU1,IL7,NBN,CLSPN,S100A12,FGR |
| GO:0 00695  0 | Response to stress | 10 | 3485 | 0.57 | 0.04  4 | APEX1,AZU1,CFHR5,NBN,CLSPN,EGLN1,S100A 12,FGR,SRPK2,LDLR |
| GO:0 00695  9 | Humoral immune response | 4 | 275 | 1.28 | 0.04  4 | AZU1,CFHR5,IL7,S100A12 |
| GO:0 03164  0 | Killing of  cells of other organism | 3 | 91 | 1.63 | 0.04  4 | AZU1,CFHR5,S100A12 |
| GO:0 04852  2 | Positive regulation of cellular process | 12 | 5579 | 0.45 | 0.04  4 | APEX1,HGF,AZU1,IL7,NBN,CLSPN,ZBTB16,EGL N1,S100A12,FGR,SRPK2,LDLR |
| GO:0 04873  1 | System developme nt | 11 | 4426 | 0.51 | 0.04  4 | FKBP4,HGF,AZU1,IL7,NBN,ZBTB16,EGLN1,FGR, SRPK2,TOP2B,LDLR |
| GO:0 05124  6 | Regulation of protein metabolic | 9 | 2828 | 0.62 | 0.04  4 | HGF,AZU1,IL7,NBN,CLSPN,EGLN1,S100A12,FGR  ,LDLR |

|  | process |  |  |  |  |  |
| --- | --- | --- | --- | --- | --- | --- |
| GO:0 00965  3 | Anatomical structure morphogen esis | 8 | 2165 | 0.68 | 0.04  81 | HGF,IL7,ZBTB16,EGLN1,FGR,SRPK2,TOP2B,LDL R |
| GO:0 04477  4 | Mitotic DNA  integrity checkpoint | 3 | 109 | 1.56 | 0.04  82 | NBN,CLSPN,TOP2B |
| GO:0 00695  5 | Immune response | 7 | 1588 | 0.76 | 0.04  91 | AZU1,CFHR5,IL7,NBN,S100A12,FGR,SRPK2 |
| GO:0 03250  2 | Developme ntal process | 12 | 5841 | 0.43 | 0.04  91 | FKBP4,APEX1,HGF,AZU1,IL7,NBN,ZBTB16,EGL N1,FGR,SRPK2,TOP2B,LDLR |
| GO:0 05117  1 | Regulation of nitrogen compound metabolic process | 12 | 5836 | 0.43 | 0.04  91 | APEX1,HGF,AZU1,IL7,NBN,CLSPN,ZBTB16,EGL N1,S100A12,FGR,SRPK2,LDLR |
|  |  |  |  |  |  |  |
| ChoE  -18:3 |  |  |  |  |  |  |
|  |  |  |  |  |  |  |
| GO:0 04213  0 | Negative regulation of T cell proliferatio n | 4 | 66 | 2.07 | 0.00  04 | SFTPD,VSIG4,CD274,LGALS9 |
| GO:0 00268  2 | Regulation of immune system process | 7 | 1514 | 0.96 | 0.00  22 | IFNLR1,ARNT,SLAMF7,SFTPD,VSIG4,CD274,LG ALS9 |
| GO:0 00181  8 | Negative regulation of cytokine production | 4 | 280 | 1.45 | 0.00  68 | SFTPD,VSIG4,CD274,LGALS9 |
| GO:2 00056  2 | Negative regulation of CD4- positive, alpha-beta T cell  proliferatio | 2 | 8 | 2.69 | 0.00  75 | CD274,LGALS9 |

|  | n |  |  |  |  |  |
| --- | --- | --- | --- | --- | --- | --- |
| GO:0 00828  5 | Negative regulation of cell  population proliferatio n | 5 | 696 | 1.15 | 0.00  8 | IFNLR1,SFTPD,VSIG4,CD274,LGALS9 |
| GO:0 04508  7 | Innate immune response | 5 | 703 | 1.14 | 0.00  8 | IFNLR1,SLAMF7,SFTPD,VSIG4,LGALS9 |
| GO:0 05170  7 | Response to other organism | 6 | 1256 | 0.97 | 0.00  8 | IFNLR1,SLAMF7,SFTPD,VSIG4,CD274,LGALS9 |
| GO:0 00181  7 | Regulation of cytokine production | 5 | 742 | 1.12 | 0.00  82 | ARNT,SFTPD,VSIG4,CD274,LGALS9 |
| GO:0 04600  7 | Negative regulation of activated T cell  proliferatio n | 2 | 13 | 2.48 | 0.01  09 | CD274,LGALS9 |
| GO:0 00960  5 | Response to external stimulus | 7 | 2310 | 0.77 | 0.01  2 | IFNLR1,SLAMF7,SFTPD,VSIG4,CD274,LGALS9,A RTN |
| GO:0 07023  4 | Positive regulation of T cell apoptotic process | 2 | 15 | 2.42 | 0.01  32 | CD274,LGALS9 |
| GO:0 00283  1 | Regulation of response to biotic stimulus | 4 | 406 | 1.29 | 0.01  45 | IFNLR1,VSIG4,CD274,LGALS9 |
| GO:0 00237  6 | Immune system process | 7 | 2481 | 0.74 | 0.01  65 | IFNLR1,SLAMF7,SFTPD,VSIG4,CD274,LGALS9,A RTN |
| GO:0 00695  5 | Immune response | 6 | 1588 | 0.87 | 0.01  65 | IFNLR1,SLAMF7,SFTPD,VSIG4,CD274,LGALS9 |
| GO:0 05077  6 | Regulation of immune response | 5 | 896 | 1.04 | 0.01  65 | SLAMF7,SFTPD,VSIG4,CD274,LGALS9 |

| GO:0 04212  7 | Regulation of cell  population proliferatio n | 6 | 1642 | 0.85 | 0.01  72 | IFNLR1,ARNT,SFTPD,VSIG4,CD274,LGALS9 |
| --- | --- | --- | --- | --- | --- | --- |
| GO:0 03270  3 | Negative regulation of interleukin  -2  production | 2 | 23 | 2.23 | 0.02  14 | SFTPD,VSIG4 |
| GO:0 04858  3 | Regulation of response to stimulus | 8 | 4114 | 0.58 | 0.03  34 | IFNLR1,ARNT,SLAMF7,SFTPD,VSIG4,CD274,LG ALS9,ARTN |
| GO:0 05089  6 | Response to stimulus | 10 | 8046 | 0.39 | 0.03  57 | CRIM1,IFNLR1,ARNT,FCRL5,SLAMF7,SFTPD,VSI G4,CD274,LGALS9,ARTN |
| GO:0 03268  9 | Negative regulation of interferon- gamma production | 2 | 35 | 2.05 | 0.03  9 | CD274,LGALS9 |
| GO:0 03273  3 | Positive regulation of interleukin  -10  production | 2 | 39 | 2 | 0.04  62 | CD274,LGALS9 |

**Supplementary table 7**: Tissue/cell type enrichment analysis of proteins associated to LPC-O-16:0 (refer to supplementary table S5

| #term ID | term descripti on | obse rved gene coun t | backg round gene count | stre ngt h | false disco very rate | matching proteins in your network (IDs | |
| --- | --- | --- | --- | --- | --- | --- | --- |
| BTO:0 000519 | Gingiva | 2 | 5 | 2.75 | 0.013  5 | 9606.ENSP00000306512,9606.ENSP000  00385675 | CXCL8,IL6 |
| BTO:0 | THP-1 | 2 | 4 | 2.84 | 0.013 | 9606.ENSP00000306512,9606.ENSP000 | CXCL8,IL6 |
| 001370 | cell |  |  |  | 5 | 00385675 |  |
| BTO:0 | Inflamm | 2 | 7 | 2.6 | 0.013 | 9606.ENSP00000306512,9606.ENSP000 | CXCL8,IL6 |
| 003861 | atory cell |  |  |  | 5 | 00385675 |  |
| BTO:0 | Nonpare | 2 | 9 | 2.49 | 0.013 | 9606.ENSP00000218388,9606.ENSP000 | TIMP1,IL6 |
| 005265 | nchymal |  |  |  | 5 | 00385675 |  |
|  | liver cell |  |  |  |  |  |  |
| BTO:0 | Fetal | 2 | 14 | 2.3 | 0.015 | 9606.ENSP00000306512,9606.ENSP000 | CXCL8,IL6 |
| 000473 | membra |  |  |  | 7 | 00385675 |  |
|  | ne |  |  |  |  |  |  |
| BTO:0 | Parenchy | 2 | 14 | 2.3 | 0.015 | 9606.ENSP00000218388,9606.ENSP000 | TIMP1,IL6 |
| 001539 | ma |  |  |  | 7 | 00385675 |  |
| BTO:0 | Mononuc | 3 | 117 | 1.55 | 0.016 | 9606.ENSP00000306512,9606.ENSP000 | CXCL8,IL6, |
| 000878 | lear cell |  |  |  | 5 | 00385675,9606.ENSP00000481357 | CCL23 |
| BTO:0 | Phagocyt | 3 | 117 | 1.55 | 0.016 | 9606.ENSP00000233997,9606.ENSP000 | AZU1,CXC |
| 001044 | e |  |  |  | 5 | 00306512,9606.ENSP00000481357 | L8,CCL23 |
| BTO:0 | Neutrop | 2 | 34 | 1.91 | 0.046 | 9606.ENSP00000233997,9606.ENSP000 | AZU1,CXC |
| 000130 | hil |  |  |  | 8 | 00306512 | L8 |
